# A Randomized Controlled Trial to Evaluate the Impact of a Novel Probiotic and Nutraceutical Supplement on Pruritic Dermatitis and the Gut Microbiota in Privately Owned Dogs

**DOI:** 10.3390/ani14030453

**Published:** 2024-01-30

**Authors:** Devon E. Tate, Jirayu Tanprasertsuk, Roshonda B. Jones, Heather Maughan, Anirikh Chakrabarti, Ehsan Khafipour, Sharon A. Norton, Justin Shmalberg, Ryan W. Honaker

**Affiliations:** 1 NomNomNow Inc., Nashville, TN 37207, USA; devon.e.tate+nnn@gmail.com (D.E.T.); jirayu.tanp+nomnomnow@gmail.com (J.T.); rbarnerjones@gmail.com (R.B.J.); 2 Ronin Institute, Montclair, NJ 07043, USA; heathermaughan@gmail.com; 3 Cargill Inc., Wayzata, MN 55391, USA; anirikh_chakrabarti@cargill.com (A.C.); ehsan_khafipour@cargill.com (E.K.); sharon_norton@cargill.com (S.A.N.); 4 Department of Comparative, Diagnostic, and Population Medicine, College of Veterinary Medicine, University of Florida, Gainesville, FL 32611, USA

**Keywords:** pruritic dermatitis, fecal microbiota, skin allergies, pruritus, dog

## Abstract

**Simple Summary:**

Given that skin allergies and pruritic dermatitis are highly prevalent in dogs, with a lack of reliable treatment methods, a dietary supplement containing a blend of probiotics, prebiotics, vitamins, nutrients and a yeast-derived postbiotic was developed with the potential to promote symptom reduction through dermatological, immune, and gastrointestinal support. To assess the impact of this supplement on clinical signs of allergy and the gut microbiome, which may influence such symptoms, in companion dogs with pruritic dermatitis, a 10-week trial was conducted. The supplement supported faster improvements and resolution of pruritus, with differences seen compared to a placebo group after 2 weeks. Simultaneously, at the end of the trial, the gut microbiome in treated dogs was enriched with three of the supplemented probiotic species, and unhealthy species were reduced. The enrolled client-owned dogs represented a variety of breeds, ages, and sizes with diverse pruritus severity, which may make the results of this trial more applicable to a larger population of dogs with pruritic dermatitis. Future trials should expand upon the use of dietary supplements with multimodal capabilities for the relief of pruritic dermatitis under stricter cohort definitions.

**Abstract:**

Pruritic dermatitis (PD) is a common presentation of canine allergic skin diseases, with diversity in severity and treatment response due to complex etiopathogenesis. Evidence suggests the gut microbiota (GM) may contribute to the development of canine allergies. A 10-week double-blind randomized controlled trial evaluated a novel probiotic and nutraceutical blend (PNB) on clinical signs of skin allergy, health measures, and the GM of privately owned self-reported pruritic dogs. A total of 105 dogs were enrolled, with 62 included in pruritus and health analysis and 50 in microbiome analysis. The PNB supported greater improvement of owner-assessed clinical signs of PD at week 2 than the placebo (PBO). More dogs that received the PNB shifted to normal pruritus (digital PVAS10-N: <2) by week 4, compared to week 7 for the PBO. While a placebo effect was identified, clinical differences were supported by changes in the GM. The PNB enriched three probiotic bacteria and reduced abundances of species associated with negative effects. The PBO group demonstrated increased abundances of pathogenic species and reduced abundances of several beneficial species. This trial supports the potential of the PNB as a supplemental intervention in the treatment of PD; however, further investigation is warranted, with stricter diagnostic criteria, disease biomarkers and direct veterinary examination.

## 1. Introduction

Pruritic dermatitis (PD) in dogs can present as a result of a variety of triggers including allergic skin diseases, infections, and parasites [1,2]. Canine atopic dermatitis (CAD) is one of the most significant causes of PD, characterized by a chronic inflammatory pruritic skin disease associated with environmental allergens (seasonal and nonseasonal) [1]. CAD is the most common presentation of atopic disease [3], estimated to impact 10–15% of dogs [4,5] with rising prevalence [3,6], and manifests as PD especially in areas with increased allergen contact and permeability [3,7,8]. Additionally, CAD characteristically results in elevated levels of IgE antibodies [1,9,10]. While CAD has been genetically linked, predisposing certain breeds [11], increasing evidence reveals environmental factors [12,13,14], secondary infections [15], oxidative stress [16], immune system disorders [17,18], changes in the gut microbiome (GM) [19,20], and skin barrier defects can also have a role in disease pathogenesis and exacerbation [3,21,22,23,24]. In particular, epidermal disturbances augment epicutaneous penetration by allergens, heightening susceptibility to allergic sensitization [3,23], or worsening of existing symptoms and inflammation [25,26]. The multiformity of disease pathways results in heterogeneity of clinical presentations and treatment responses among allergic dogs [1,3,5] and, subsequently, diverse treatment options with multimodal actions need to be explored.

While evidence supports certain dietary interventions such as polyunsaturated fatty acids for canine skin health and dermatitis [26,27,28], nutritional support for canine dermatological conditions is an ongoing area of research. The skin’s intricate antioxidant system plays an integral role in protecting the skin barrier, and lowered concentrations of key antioxidants have been associated with poor skin condition [29], making antioxidants a possible defense mechanism [16]. Emerging evidence supports an abundance of nutritional components, vitamins, and minerals for protection against skin barrier abnormalities in dogs [25,30,31,32]. B vitamins and vitamin E, both critical to canine skin health [25,33,34], have shown promise as an intervention for canine pruritic skin diseases [21,29]. Novel nutritional interventions for PD extend beyond solely cutaneous considerations. Lutein and other carotenoids, which are efficient antioxidants, have been shown to reduce oxidative stress and inflammatory response in animal studies [35,36], and in dogs have been demonstrated to dose-dependently enhance immune response [37,38,39,40]. A yeast-derived postbiotic (from *Saccharomyces cerevisiae*) has been demonstrated to benefit seasonal allergies and immune conditions in humans and in preliminary animal studies [41,42], as well as to improve serum IgA levels in dogs [43] (low IgA levels are commonly associated with atopy [44]). Additionally, an *S. cerevisiae* fermentation product provided to research dogs indicated potentially beneficial effects on immune parameters, inflammation, and microbial populations [45,46], all of which may improve clinical signs of allergy.

An imbalance of the microbiota (dysbiosis) in companion animals is suspected to be associated with allergic diseases [19,20,47]. Given the immunomodulatory properties of the intestinal microbiome, the emerging relationship between the gut and skin, and its arising role in the pathogenesis of pruritic dermatitis [19,20,47], it can be hypothesized that ingredients that alter colonic microbiota in turn could improve clinical signs [19,30,48,49]. Furthermore, in humans, differences in distinctive fecal microbiome profiles have been identified between those with atopic dermatitis and healthy controls [50,51] and recently this has been similarly shown with atopic dogs [20,52], highlighting an opportunity for intervention. Probiotics and prebiotics have both proven to be successful ingredients for shaping the canine microbiome [53], which may be helpful for the prevention and treatment of allergic symptoms [48,54], as evidenced by an increasing number of human and animal studies [55,56,57,58,59]. In dogs, preliminary evidence highlights the potential of several probiotic strains for support of PD [49,60,61,62,63,64,65], while prebiotics have shown immunomodulatory properties, which may reduce clinical signs of allergy given their relationship to immune disorders [66,67,68,69].

The aim of this study was to examine the impact of a probiotic and nutraceutical blend (PNB) on the severity of PD in privately owned dogs over 10 weeks of supplementation. Digital evaluations, including a validated scale and an owner-friendly adaptation of a traditional scoring system, were employed to measure changes in clinical signs of pruritus and allergy. The formulation of the PNB supplement was guided by limited but available evidence on selected ingredients, as described above, and this marks the first time their collective action has been examined in household owner-indicated pruritic dogs. The association between pruritus improvement and the GM was also explored. While many conventional PD treatments exist [5], given the multifaceted nature of PD it is paramount that supplemental therapies with diverse actions are investigated.

## 2. Materials and Methods

### 2.1. Animals

**Figure 1:**
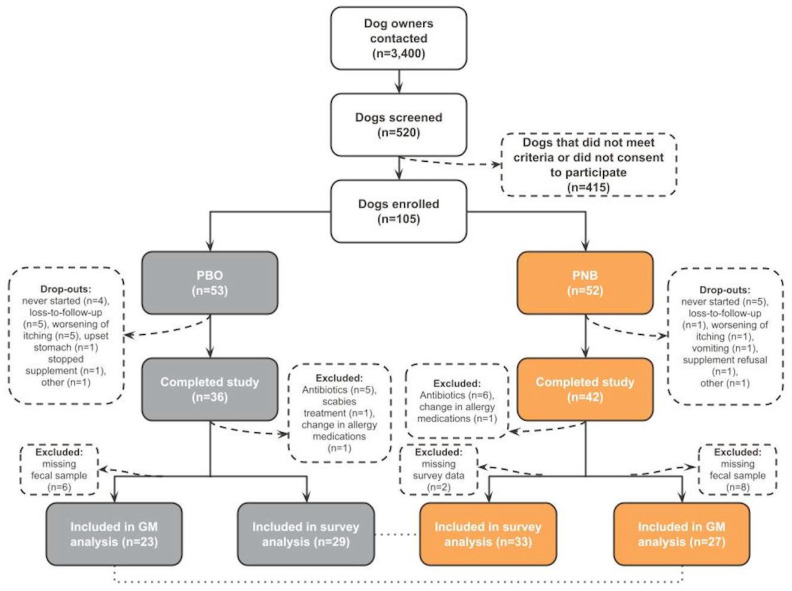
Trial flowchart. PBO—placebo group; PNB—probiotic and nutraceutical blend group; GM—gut microbiota.

A total of 3400 dog owners were contacted for possible enrollment. All dogs were privately owned and customers of NomNomNow Inc. (Nashville, TN, USA). A total of 520 dogs were screened for eligibility, and 105 eligible dogs were enrolled in the study (Figure 1). Prior to enrollment, owners of the eligible dogs consented electronically to the use of their dog’s deidentified information for publication purposes, as well as all study guidelines and parameters.

Eligibility was based on owner-reported screening responses (Figure S1) as follows: aged 1–12 years; body condition score (BCS) of 4–6 (ideal to overweight but not obese) on the 9-point scale BCS system [70]; 22.7 kg or less; and with presence of seasonal or nonseasonal pruritic dermatitis (PD). The enrolled dogs were absent of any concurrent systemic disease, not pregnant or lactating, and having had no surgery within the previous 3 months. Use of allergy medications was permitted during the study only when dogs were using them for at least one month prior to the trial, had persistent clinical signs of skin allergy, and owners agreed to adhere to their medication regime (dose and frequency) throughout the study period. However, use of oral antibiotics, antifungals, or antiparasitics (with the exception of regular flea preventatives), as well as dietary supplements with overlapping or similar ingredients to those given in this trial, were not permitted within one month of beginning the trial or at any point throughout. All enrolled dogs were consuming any combination of four commercially available fresh canine diets (NomNomNow Inc., Nashville, TN, USA) formulated to meet AAFCO requirements for all life stages. Dogs maintained this diet for at least one month prior to and throughout the trial period. Ingredients and guaranteed nutrient analysis of the fresh diets have been previously described [71,72]. No restrictions on treat intake, breed, sex, or spay/neuter status were made. Specific details on all eligibility criteria can be found in Table S1.

### 2.2. Supplement Interventions and Study Design

The enrolled dogs were randomized to receive either the full probiotic and nutraceutical blend (PNB; *n* = 52) or a placebo (PBO; *n* = 53) for a total of 10 weeks. Supplements were not commercially available and owners were blinded to their treatment group and had no awareness of intended supplement appearance. All supplements used in this study were fine powders and administered using a 2 g measuring scoop (Figure S2). Dog owners were instructed to mix their provided supplement once daily into the participating dog’s first meal of the day following these dosing guidelines by dog weight: ≤4.5 kg—half of the provided scoop (1 g); 4.6–11.3 kg—level scoop (2 g); 11.4–22.7 kg—two level scoops (4 g). The probiotic and nutraceutical blend (PNB) consisted of a combination of the following probiotic bacterial species: *Lactobacillus rhamnosus*, *Bifidobacterium bifidum*, *Bifidobacterium infantis*, *Bifidobacterium animalis*, *Lactobacillus acidophilus*, and *Lactobacillus casei*. The PNB also contained vitamin E (as D-alpha tocopheryl acetate), vitamin B_3_ (as niacinamide), vitamin B_6_ (as pyridoxine HCI), vitamin B_5_ (as D-calcium pantothenate), inositol (as myo-inositol), choline (as choline L-bitartrate), lutein (as lutein esters extracted from marigold petals (*Tagetes erecta*)), and prebiotic fibers mannanoligosaccharides and fructooligosaccharides. Finally, a proprietary *Saccharomyces cerevisiae* based postbiotic (EpiCor^®^ postbiotic, Cargill Inc., Ankeny, IA, USA), was used in the PNB, which has been proven to be safe for dogs [43]. The placebo (PBO) consisted purely of maltodextrin, which was also used as a filler in the PNB to optimize flow properties during manufacturing.

To assess changes in PD clinical signs and health outcomes, online surveys were completed by owners (Section 2.3). Additionally, stool samples were collected by dog owners for gut microbiome analysis (Section 2.4). Owners were provided with the opportunity to report any adverse events and could willingly withdraw their dog at any point. Participants were also asked to complete an online health assessment (consisting of five questionnaires) during baseline stool sample registration which provided additional subject information and has been previously described in other research studies [71,73].

### 2.3. Health Survey

Owner-assessed levels of pruritus, condition of five individual body sites, quality of life (QOL), skin and coat condition, and general wellness [32] were determined at baseline (day 0) and the ends of weeks 2, 4, 7 and 10, via a series of online surveys (Qualtrics CoreXM). Surveys were provided via email on the appropriate dates, and also contained reminders about study adherence. With the exception of the baseline survey, treatment acceptance and compliance were assessed in all surveys. All survey questions can be viewed in Figure S3.

A digital version of the canine pruritus severity scale, a validated 10-point pruritus visual analog scale (digital PVAS10) for owner-assessed severity of pruritus [74,75], was used in the survey assessments. The scale combines favorable aspects of alternative pruritus assessment scales and has been shown to be a convenient and reliable pruritus evaluation tool for dog owners [74]. The digital PVAS10 included severity and behavioral descriptors along a visual analogue scale without discrete markings. Based on the six anchored descriptors, owners indicate where on the slider their dog’s pruritus severity lies, which is then translated into a continuous score between 0 and 10 [74,75]. Pruritus severity thresholds can be classified as follows: normal to very mild (digital PVAS10-N: <2), mild (digital PVAS10: 2–3.5), moderate (digital PVAS10: 3.6–5.5), and severe (digital PVAS10: ≥5.6) [75,76].

A scoring system, referred to herein as the Owner Assessed-Skin Allergy Severity Index (OA-SASI), was generated for the assessment of clinical skin lesions on five body sites on dogs. This scale was derived from the Canine Atopic Dermatitis Extent and Severity Index (CADESI)-4, a robust scoring system developed for the assessment of 20 body sites by a veterinarian or clinician [77]. Significant adaptations were made to the CADESI-4 for ease of owner assessment and compliance. The OA-SASI simplifies the traditional scale to include broader, more generalized body sites (face, ears, paws, limbs, and underside), but maintains the types of lesions examined (erythema, lichenification, and alopecia/excoriation) and severity grades (0 = None; 1 = Mild; 2 = Moderate; 3 = Severe) [77], however, descriptions have been adapted to layman’s terms. The maximum total score that can be achieved in the OA-SASI is 45, indicating all five body sites experienced all three lesion types with the highest severity. The validated CADESI-4 scale and the OA-SASI should be considered separate severity assessments and should not be directly compared.

### 2.4. Fecal Collection and DNA Sequencing

To assess the fecal microbiota, all dog owners were provided with two identical Nom Nom Plus Microbiome Testing Kits (Nashville, TN, USA), and instructed to use one kit to provide a stool sample at baseline (day 0) and the second kit to provide a stool sample at the end of the study (week 10). These collection kits have been previously utilized in other research studies [53,71,73]. Stool samples from both baseline and week 10 were received and processed in a single batch by Diversigen Inc. (New Brighton, MN, USA). DNA extraction and library construction protocols were performed as previously described [53,78], with the exception that DNA was extracted using the Zymogen Quick-DNA Fecal/Soil Microbe 96 Mag Bead kit (Zymo Research, Irvine, CA, USA) using Powerbead Pro (Qiagen, Redwood City, CA, USA) plates with 0.5 and 0.1 mm ceramic beads. Extraction controls included a no template control (water) and a characterized homogenized stool. All samples were quantified with Quant-iT Picogreen dsDNA Assay (Invitrogen, Carlsbad, CA, USA). Subsequently, DNA amplification and library construction were performed with the Nextera XT DNA Library Preparation Kit (Illumina Inc., Foster City, CA, USA).

### 2.5. Gut Microbiome Shotgun Metagenomic Sequencing and Taxonomy and Functional Annotation

Stool sample shotgun metagenomic sequencing was performed using the BoosterShot^®^ shallow metagenomic sequencing service, as previously described [78]. For quality control, single-end shotgun reads were trimmed and processed using Shi7 [79]. The sequences were then aligned to a curated database containing all representative genomes in RefSeq with additional manually curated strains. Alignments were made at 97% identity against all reference genomes. Each input sequence was compared to every reference sequence in the Diversigen Venti database using fully gapped alignment with BURST using default settings [80]. Ties were broken by minimizing the overall number of unique operational taxonomic units (OTUs). For taxonomy assignment, each input sequence was assigned the lowest common ancestor that was consistent across at least 80% of all reference sequences tied for best hit using the Genome Taxonomy Database toolkit (GTDB-Tk) [81].

### 2.6. Statistical Analysis

All analyses were performed using R versions 4.0.3 (survey data) and 4.1.0 (GM data), with statistical significance set at α = 0.05. Continuous variables are expressed as mean ± SD, and categorical variables are expressed as count (%). GM relative abundances are expressed as median [IQR].

#### 2.6.1. Analysis of Pruritus and Health Outcomes

The Wilcoxon rank-sum test (Mann–Whitney U Test) was performed on continuous variables to test for statistically significant differences in all measured outcomes between the supplement groups. For categorical data, such as improvement rates or differences in occurrence, Fisher’s exact test was performed. The Wilcoxon matched-pairs signed-rank test was used when comparing continuous variables within supplement groups. Statistical interactions between time and supplement group were determined by two-way repeated measures ANOVAs, and the effect of time was assessed using Wilcoxon signed-rank tests, comparing the value at each timepoint to baseline within each group. Heterogeneity of treatment effect (HTE) analysis was also conducted on primary clinical outcomes. HTE seeks to evaluate the nonrandom variability in treatment impact [82]. HTE was performed by examining targeted subgroups to identify key characteristics in the study population that were associated with improved treatment response. Normality was examined for continuous data with the Shapiro–Wilk test, and nonparametric statistics are primarily reported due to skewed distributions. In the case where a parametric test is utilized, log-transformed results may be reported only if skewness was improved. No adjustment of *p* values was performed for multiple comparisons.

#### 2.6.2. Gut Microbiome Analysis

Correlation analysis (Spearman’s ρ) was performed to investigate the relationship between two continuous variables. For α-diversity, a filtering step was performed to remove taxa (at the OTU level) with fewer than or equal to 2 reads in >5% of the samples. After filtering, the depth per sample ranged from 856,696 to 8,696,886 reads, and a total of 2622 taxa were used for the analysis. Similar to the approach previously described [73], the α-diversity metrics including richness, Pielou’s evenness, and Shannon diversity index (H) were computed by rarefying the samples to various depths. One hundred iterations were performed at each depth and mean values were used as the estimate of these measures in each sample. Repeated measures ANOVAs and Wilcoxon signed-rank tests were used to compare changes of the α-diversity metrics from baseline to week 10.

For β-diversity and differential abundance analysis, the taxonomy table was aggregated at the species level. A filtering step was performed to remove species with fewer than or equal to 5 reads in >10% of the samples. After filtering, a total of 682 species were used for the analysis (Figure S4). The average sequencing depth was 3,912,007 ± 1,603,957 per sample, and the depth per sample ranged from 856,009 to 8,692,915 reads. Species-level taxonomy tables were natural log (x + 1)-transformed and principal coordinate analysis (PCoA) was performed using Bray–Curtis dissimilarity calculated with the vegan package in R [53,83]. Using the vegan package, permutational multivariate analysis of variance (PERMANOVA) was performed using Bray–Curtis dissimilarity with 10,000 randomizations by including groups and time points to assess the differences in community composition [83]. The Wilcoxon signed-rank test was used to assess the changes along the PCoA axes from baseline to week 10 in each intervention group.

For functional annotation, sequence reads were matched directly, using alignment at 97% identity, to multiple Kyoto Encyclopedia of Genes and Genomes (KEGG) databases, including KEGG Orthology groups (KO), KEGG ENZYME, KEGG MODULE, and KEGG PATHWAY (level 3) [84,85]. KEGG terms with fewer than or equal to 5 reads in >10% of the samples were removed as part of the quality filtering process, and 3867 KO terms, 2252 enzymes, 296 modules, and 185 pathways were used in subsequent analyses. PCoA and PERMANOVA were repeated using the same steps as described above for the functional table with KO terms.

Differential abundances of bacterial species and KEGG terms were assessed using a negative binomial generalized linear model (GLM), using the differential expression analysis for sequence count data version 2 package (DESeq2) [73,86]. Species and KEGG terms with |log2(fold change (FC)| > 2 and adjusted *p*-values < 0.05 were considered statistically significant. Adjustment of *p* values was performed to control false discovery rate (FDR) with the Benjamini–Hochberg method [87].

## 3. Results

### 3.1. Cohort Description

Of the 105 dogs enrolled in the study, 62 were included in survey analysis, and 50 were included in microbiome analysis. A total of 27 dogs did not complete the study, an additional 14 dogs were excluded from all analyses for compliance issues, and due to missing data two more dogs were excluded from survey analysis and 14 from microbiome analysis (Figure 1). There were no significant differences between groups for the number of dogs excluded from both the survey and gut microbiome (GM) analysis. A total of 13 enrolled dogs reported adverse events, eight in the placebo (PBO) group (increased pruritus, *n* = 7; gastrointestinal issue, *n* = 1) and five in the probiotic and nutraceutical blend (PNB) group (increased pruritus, *n* = 4; vomiting, *n* = 1). The number of adverse events was not significantly different between groups (15.1% (8/53) in PBO, 9.6% (5/52) in PNB, *p* = 0.555, Fisher’s exact test) and, overall, both supplements were reported to be well accepted throughout the course of the study.

Of the 62 dogs included for survey analysis, baseline characteristics (owner-reported) were well balanced among groups, with the exception of hours spent outside (Table 1). Dogs were on average 7.55 ± 3.4 years old, 51.6% female (*n* = 32, 16 PBO, 16 PNB), 98.4% spayed/neutered (*n* = 62, 32 PBO, 29 PNB), and weighed 9.41 ± 6.0 kg (illustrating the breed diversity). While the majority of dogs reported the use of allergy-specific medications (62.9% total, *n* = 39; 18 PBO, 29 PNB), which were sustained throughout the study, visible skin allergy symptoms were still reported: 24.2% oclacitinib (*n* = 15; seven PBO, eight PNB); 22.6% medicated and hypoallergenic shampoos and wipes (*n* = 14; six PBO, eight PNB); 20.9% antihistamines (*n* = 13; eight PBO, five PNB); 11.3% allergen immunotherapy (*n* = seven; two PBO, five PNB); 11.3% other (*n* = seven; two PBO, five PNB); 6.5% lokivetmab (*n* = four; one PBO, three PNB); 4.8% steroids (*n* = three; two PBO, one PNB); and 3.2% topicals (*n* = two; one PBO, one PNB). Additional information on the individual characteristics of the dogs, including breed and allergy-specific medications, is available in Table S2.

### 3.2. Health Outcomes

#### 3.2.1. Canine Pruritus Severity Scale (Digital PVAS10)

**Figure 2:**
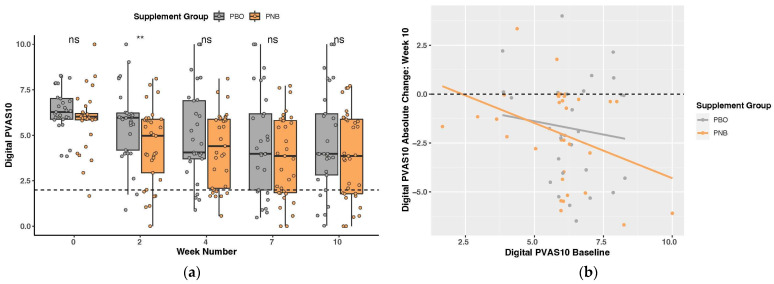
Canine pruritus severity score (digital PVAS10) plots: (**a**) Boxplot for all weeks. Dots represent the digital PVAS10 scores of individual dogs; dashed line—normal severity (digital PVAS10-N = <2); ns *p* > 0.05, ** *p* < 0.01; and (**b**) absolute change in digital PVAS10 score at week 10 vs. baseline score.

Digital PVAS10 scores (0–10; pruritus severity) at week 2 were significantly different between groups (*p* = 0.009, Wilcoxon rank-sum test), with dogs taking the PNB (4.41 ± 2.08) having significantly lower scores than the PBO group (5.67 ± 2.02) (Figure 2a). The percentage of dogs in each digital PVAS10 severity threshold for each week of the trial can be found in Table 2. Dogs administered the PNB had the highest percentage in the normal pruritus range for all weeks of the study; however, the differences between groups were not significant. Compared to baseline, the dogs administered the PNB had more subjects with normal to very mild pruritus (digital PVAS10-N: <2) by week 4 (*p* = 0.054, Fisher’s exact test). Considering only dogs with an initial severity in the moderate-to-severe range (digital PVAS10 ≥ 3.6; PBO = 29, PNB = 31), this became statistically significant (*p* = 0.024), while dogs administered the PBO did not reach a significant difference until week 7. Further, for dogs with moderate-to-severe baseline pruritus, the normal-to-mild range (digital PVAS10-N2M <3.6) is an appropriate outcome threshold [76]. Compared to baseline, those receiving the PNB had more subjects in the normal-to-mild range as early as week 2 (*p* = 0.011, Fisher’s exact test), as opposed to week 4 for dogs receiving the PBO.

A two-way repeated measures ANOVA showed a significant time effect on digital PVAS10 scores (*p* = 4.55 × 10^−9^), with the PNB having larger decreases from baseline for all weeks. To visualize the treatment impact [75], the individual digital PVAS10 score for each dog before and after 10 weeks can be seen in Figure S5, as well as the median scores in Table S3. Absolute and relative change from baseline, as well as score decrease per week from baseline and the week prior, were explored; however, this yielded no significant differences between the PBO and the PNB groups.

#### 3.2.2. Owner Assessed-Skin Allergy Severity Index (OA-SASI)

**Figure 3:**
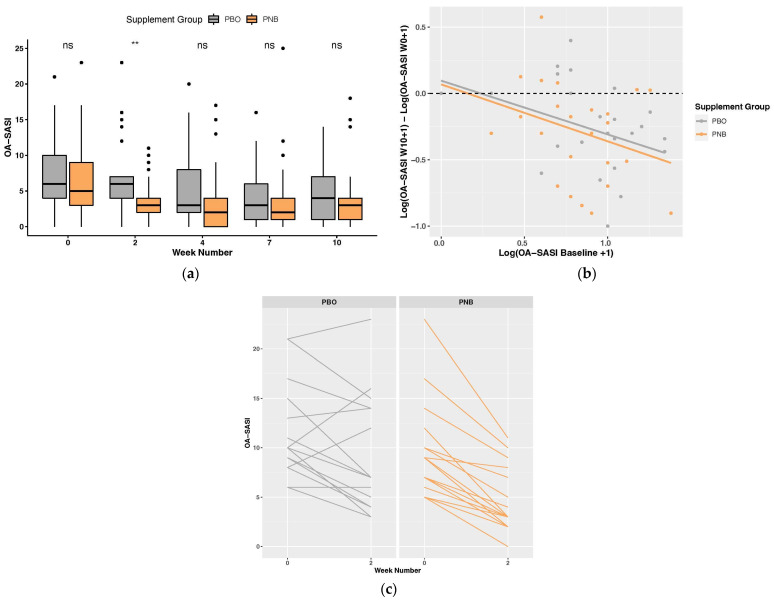
Owner Assessed Skin Allergy Severity Index (OA-SASI) score plots: (**a**) Boxplot for all weeks. ns *p* > 0.05, ** *p* < 0.01; (**b**) absolute change in OA-SASI score at week 10 (log-transformed) vs. baseline score (log-transformed); and (**c**) individual OA-SASI change baseline to week 2 in subgroup with high OA-SASI at baseline (≥median score within group; PBO = 16, PNB = 18).

OA-SASI scores (0–45; modified severity index) were significantly different between groups at week 2 (*p* = 0.002, Wilcoxon rank-sum test). Dogs administered the PNB (3.61 ± 3.22) had significantly lower scores than those administered with PBO (7.10 ± 5.46) (Figure 3a). A two-way repeated measures ANOVA showed a significant time effect on log-transformed OA-SASI score (*p* = 4.88 × 10^−11^), with the PNB group significantly decreasing compared to baseline for all weeks. The individual response of each dog from week 0 to week 10 can be seen in Figure S6. Percentage change from baseline was converted into a categorical scale (score 0 = increased score; score 1 = <25% reduction; score 2 = 25–49% reduction; score 3 = 50–74% reduction; score 4 = ≥75% reduction), as is recommended with the traditional CADESI-4 [77]. After applying this conversion to the OA-SASI score no significant differences were found between the groups at any time point (Table S4). Absolute and relative change from baseline, as well as score decrease per week from baseline and the week prior, were assessed; however, no significant differences were found.

Examining the components of OA-SASI at week 2 (Table S5), there were significant differences among all three lesions examined (alopecia/excoriation, erythema, lichenification), with the PNB group having lower scores than PBO. The individual body sites (face, ears, paws, limbs, and underside) were further examined for each OA-SASI component. The paws were found to be the major body site contributing to differences in alopecia and erythema at week 2 between the groups. Differences in lichenification were similarly driven by the paws, but the ears and underside were also found to be contributing body sites.

#### 3.2.3. Heterogeneity of Treatment Effect and Exploratory Subgroup Analyses

To explore heterogeneity of treatment effect (HTE), we examined the relationship between the health outcomes and baseline risk. The absolute change in digital PVAS10 score at week 10 was significantly correlated to the baseline score in the PNB group (ρ = −0.377, *p* = 0.030), but not in the PBO group (ρ = −0.051, *p* = 0.794), with a larger change corresponding to a higher baseline score (Figure 2b). For the OA-SASI score (log-transformed), a correlation between absolute change at week 10 and the baseline score for the PNB group (*r* = −0.42, *p* = 0.016) was observed, but not in the PBO group (*r* = −0.35, *p* = 0.062) (Figure 3b). Looking into only the subjects with high OA-SASI scores at baseline (≥median score within group; PNB = 18, PBO = 16), there was a significant difference in the percentage of dogs that showed an improvement at week 2 (*p* = 0.016, Fisher’s exact test), with 100% of dogs administered the PNB improving compared to only 68.75% of PBO dogs (Figure 3c). Similarly, when looking into only subjects with high digital PVAS10 at baseline (≥median score within group; PNB = 17, PBO = 15), the proportion of dogs showing digital PVAS10 improvement was higher in PNB dogs than PBO dogs at all time points, despite the difference not reaching statistical significance. The PBO group also had significantly higher digital PVAS10 than the PNB group at baseline (*p* = 0.026).

Additional targeted subgroups based on subject characteristics (sex, age, weight, BCS, spay/neuter status, and coat type) and medication usage (allergy-specific, flea, and shampoo) were evaluated for both the digital PVAS10 and OA-SASI outcomes, but were not strongly associated with the magnitude of digital PVAS10 or OA-SASI changes. Subgroup analysis was exploratory, with the goal of understanding any differential impacts of treatment and further evaluating efficacy.

For the OA-SASI scores under stricter diagnostic criteria (for atopic dermatitis) as described previously [88] (age of onset < 3 years; PNB = 20, PBO = 15), there was a significant difference between groups at week 2 (*p* = 5.70 × 10^−5^, Wilcoxon rank-sum test) and week 4 (*p* = 0.017). At both time points, dogs administered the PNB had significantly lower scores than dogs administered the PBO (Figure S7). It should be noted, however, that baseline OA-SASI scores were also significantly lower in the PNB group in this subpopulation (*p* = 0.026, Wilcoxon rank-sum test). To account for baseline differences, relative changes in OA-SASI scores from baseline were examined; the difference at week 2, but not week 4, approached statistical significance (week 2: *p* = 0.054, week 4: 0.491, Wilcoxon rank-sum test). Nevertheless, a two-way repeated measures ANOVA (log-transformed) showed a significant time (*p* = 2.16 × 10^−5^) and group (*p* = 4.00 × 10^−3^) effect on OA-SASI score when age of onset was restricted, with the PNB group having significantly greater OA-SASI reduction than the PBO group. When digital PVAS10 scores were examined in this subgroup, the findings remained similar to the analysis in all subjects—the PNB group had a lower score than the PBO group at week 2 (*p* = 0.016, Wilcoxon rank-sum test), and only the time effect was significant (*p* = 6.35 × 10^−5^, two-way repeated measures ANOVA). There were no significant differences between groups for digital PVAS10 or OA-SASI at any time point in the subpopulation with age of onset > 3 years (PNB = 13, PBO = 14).

Subgroups based on seasonality of clinical signs were also explored, given that the changing of seasons could also have affected the severity of symptoms. In those subjects with nonseasonal clinical signs of PD (PNB = 26, PBO = 18), PNB subjects had lower OA-SASI than PBO subjects at week 2 (*p* = 0.002, Wilcoxon rank-sum test), week 4 (*p* = 0.019), and week 10 (*p* = 0.031) (Figure S8). A two-way repeated measures ANOVA (log-transformed) subsequently revealed significant group (*p* = 0.011) and time factors (*p* = 1.23 × 10^−6^), but the group*time interaction term was not significant. Likewise, when digital PVAS10 score was examined in this subgroup, group (*p* = 0.026, two-way repeated measures ANOVA) and time factors (*p* = 4.44 × 10^−6^) were significant, but not the group*time interaction. However, digital PVAS10 differences between groups were also seen at baseline (*p* = 0.010, Wilcoxon rank-sum test) (Figure S9). The seasonal subgroup was not significantly different at any point for digital PVAS10 or OA-SASI, although the sample sizes were small (PNB = 7, PBO = 11).

#### 3.2.4. Additional Health Outcomes

**Figure 4:**
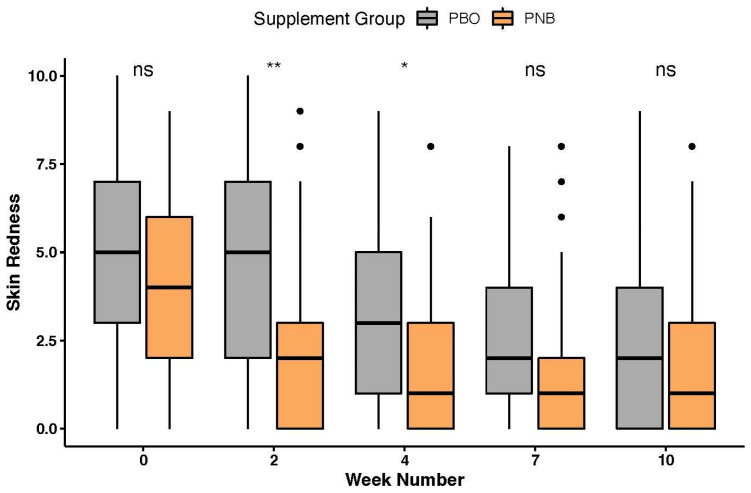
Boxplot of skin redness [from 0 (not red at all) to 10 (extremely red)] for all weeks. ns *p* > 0.05, * *p* < 0.05, ** *p* < 0.01.

Skin redness, scored by owners from 0 (“not red at all”) to 10 (“extremely red”), was significantly different at weeks 2 (*p* = 0.003, Wilcoxon rank-sum test) and 4 (*p* = 0.018, Figure 4), with dogs administered the PNB having lower skin redness scores than dogs administered the PBO. A two-way repeated measures ANOVA (log-transformed) revealed both a significant time (*p* = 7.29 × 10^−12^) and group effect (*p* = 0.012), but not a significant group*time interaction on redness, with the PNB group having significantly lower erythematous than the PBO group.

Additional general health outcomes differed between intervention groups at specific time points. Dogs administered the PNB had higher quality of life (QOL) ratings at week 2 (*p* = 0.025, Wilcoxon rank-sum test) and week 4 (*p* = 0.046, Figure S10) compared to PBO. Additionally, week 2 QOL ratings were significantly lower than the start of the trial (*p* = 0.031, Wilcoxon signed-rank test) within the PBO group, while no significant changes from baseline were found within the PNB group. The owner’s ratings for how disruptive their dog’s skin condition was to their household were also significantly lower in the PNB group than the PBO group at week 2 (*p* = 0.017, Wilcoxon rank-sum test). Similar to baseline (Table 1), dogs administered the PNB spent less time outside (≤1 h per day) compared to dogs taking the PBO at week 2 (*p* = 0.021, Fisher’s exact test), week 4 (*p* = 0.010), and week 7 (*p* = 0.044); however, hours spent outside were not associated with pruritus severity at any time point in either group. Additional health outcomes were found to be unbalanced between the two intervention groups at baseline; dogs administered the PBO reported lower overall health than dogs administered the PNB (*p* = 0.047, Wilcoxon rank-sum test); scratching amount was lower in the PNB group than PBO at baseline (*p* = 0.047, Wilcoxon rank-sum test) and week 2 (*p* = 0.039). However, when the scratching amount was expressed as % change from baseline, the difference between groups at week 2 was no longer significant (*p* = 0.627, Wilcoxon rank-sum test). Additional health, skin and coat, behavioral, and general wellness outcomes were not statistically different between groups by the end of the trial at week 10 (Table S6).

### 3.3. Gut Microbiome

#### 3.3.1. Gut Microbiome Diversity

**Figure 5:**
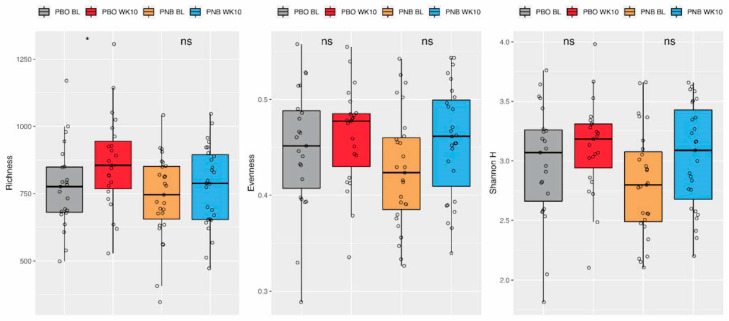
α-diversity metrics (richness, Pielou’s evenness, and Shannon H) from baseline to week 10. ns *p* > 0.05, * *p* < 0.05.

From the 50 subjects included in microbiome analysis (PBO = 23, PNB = 27), a total of 100 samples were collected from both time points (baseline and week 10). A rarefaction curve was generated for sequencing depths ranging from 50,000 to 850,000 reads. Since the depth at 850,000 reads represented the highest OTU count that included all available samples, it was used to assess the α-diversity. There was a significant increase in richness from baseline to week 10 in the PBO but not in the PNB group (Figure 5, *p* = 0.048, Wilcoxon’s signed-rank test for paired samples). On the other hand, the increase of Pielou’s evenness (*p* = 0.058) and Shannon H (*p* = 0.062) from baseline to week 10 were borderline significant in the PNB group. No significant interaction between the group and the time point was observed in the repeated measures ANOVA model for any of the α-diversity metrics.

**Figure 6:**
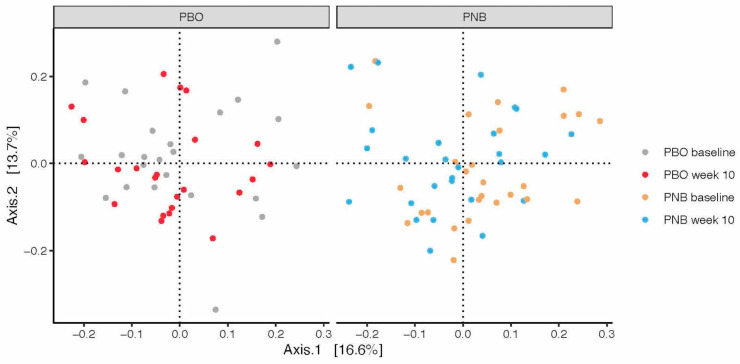
Principal coordinate analysis (PCoA) plot. PCoA axes 1 and 2, respectively, explained 16.6% and 13.7% of the variance of the abundance of gut microbiome at the species level.

**Figure 7:**
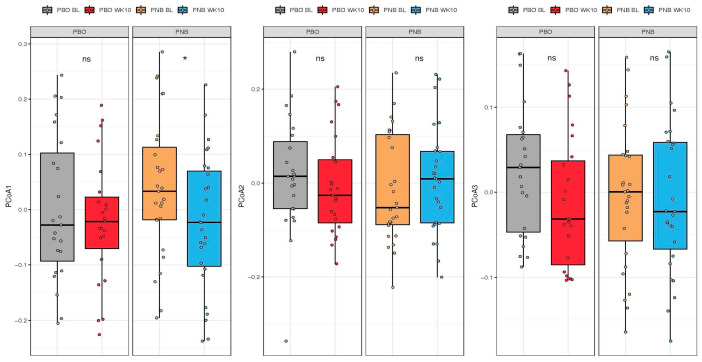
Scores of the first 3 principal coordinate analysis (PCoA) axes in subjects receiving the probiotic and nutraceutical blend (PNB; *n* = 27) or the placebo (PBO; *n* = 23) at baseline and week 10. ns *p* > 0.05, * *p* < 0.05.

Changes in β-diversity were examined with principal coordinate analysis (PCoA). At the species level, the first two axes (PCoA1 and PCoA2) accounted for 16.6% and 13.7% of the variation, respectively (Figure 6). The eigenvalues of the first 25 PCoA axes are shown in Figure S11, with each of the first three axes explaining >6% of the variance. Spatial separation along the first two PCoA axes was observed between the two time points (*p* = 0.008 for the time point term, PERMANOVA, using the Bray–Curtis dissimilarity matrix). When each of the first three axes were examined separately (Figure 7), a significant shift from baseline to week 10 along the PCoA1 axis, but not other axes, was observed only in the PNB group (*p* = 0.030, Wilcoxon’s signed-rank test for paired samples, Figure S12). No significant interaction between the group and the time point was observed in the repeated measures ANOVA model for any of the first three PCoA axes.

#### 3.3.2. Gut Microbiome Abundance

The phyla Proteobacteria (37.11% [15.64–67.64%]), Firmicutes (14.12% [5.71–36.26%]), Firmicutes_A (11.65% [5.22–23.31%]), and Bacteroidota (3.74% [0.37–15.73%]) constituted the majority of the GM in the 100 samples collected in this study (Figure S13). These four phyla dominated the GM of samples at both time points in both treatment groups.

**Figure 8:**
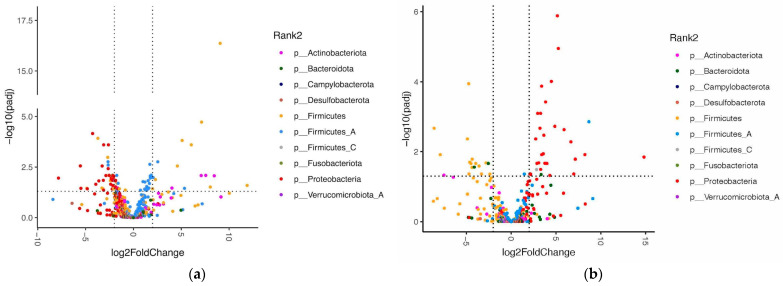
Volcano plots demonstrating the fold-change (FC) in the differential abundance analysis of gut bacteria at the species level at week 10 compared to week 0. Vertical dashed lines show log2FC at 1 and −1 (i.e., FC at 2 and −2). Horizontal dashed line shows −log10 (adjusted *p*) = 2 (i.e., adjusted *p* = 0.01). Each point represents a distinct species and points are colored by phylum. (**a**) The probiotic and nutraceutical blend (PNB) group (*n* = 27); and (**b**) the placebo (PBO) group (*n* = 23).

Differential abundance analysis demonstrated significant changes in the abundances of 53 species in the PNB group and 48 species in the PBO group from baseline to week 10 (Figure 8a,b, Table 3 and Table 4). The abundances of three of the six probiotic species included in the PNB formulation (*L. rhamnosus*, *B. animalis*, and *L. acidophilus*) significantly increased at week 10 in the PNB group. On the other hand, *B. animalis* was the only probiotic species whose abundance significantly changed (decreased) in the PBO group between baseline and week 10. There was a clear trend of an increase in all six supplemented probiotics in the PNB group but not the PBO, even though not all of them reached statistical significance with differential abundance analysis (Figure S14).

Species in the phyla Proteobacteria and Firmicutes showed strikingly different patterns of change in their abundances between the PNB and PBO groups. Twenty-eight species in the Proteobacteria phylum significantly increased and none decreased in the PBO group. In comparison, 26 species in the Proteobacteria phylum significantly decreased in the PNB group, including known canine pathogens *Proteus mirabilis*, *Citrobacter freundii*, and *Klebsiella pneumoniae*, and no Proteobacteria increased. In the PBO group, 14 species in the Firmicutes phylum significantly decreased and none increased. In comparison, eight species of Firmicutes significantly increased (including the supplemented probiotics *L. rhamnosus* and *L. acidophilus*) and nine species of Firmicutes significantly decreased in the PNB group. To explore functional annotation, further analysis was done on KEGG terms (Figures S15 and S16, Tables S7 and S8).

While the majority of the subjects in the PNB group demonstrated a consistent shift towards lower scores along the PCoA1 axis, the magnitude of responses varied among individual dogs. Greater shift (greater reduction) in PcoA1 score from baseline to week 10 was correlated with higher PcoA1 score at baseline (ρ = −0.59, *p* = 1.16 × 10^−5^, Figure S17), as well as with lower α-diversity at baseline (richness: ρ = 0.37, *p* = 8.44 × 10^−3^; evenness: ρ = 0.42, *p* = 2.44 × 10^−3^; Shannon H: ρ = 0.42, *p* = 2.93 × 10^−3^, Figure S18). These correlations remained statistically significant after adjusting for the group variable.

It has been previously reported that GM responses to an intervention are associated with baseline GM [53,71]. Subjects in the PNB group were divided into tertiles based on the magnitude of shift along the PCoA1 axis and baseline GMs between subjects in the first and third tertiles were compared. Species in the phyla Proteobacteria, Firmicutes_A, Actinobacteriota, and Bacteroidota had strikingly different abundances before supplementation with the PNB (Figure S19, Table S9). Species in the Proteobacteria phylum (24 species) were found to be more abundant in the third tertile (largest PCoA1 shift) than the first tertile (smallest PCoA1 shift in the same direction as all subjects in the PNB group, or PCoA1 shift in the direction opposite from the shift observed in all subjects in PNB), and never more abundant in the first tertile than the third tertile at baseline. On the other hand, species in the Firmicutes_A (24 species), Actinobacteriota (nine species), and Bacteroidota (five species) phyla were more abundant in the first tertile than the third tertile.

#### 3.3.3. Gut Microbiome Changes and Pruritus Improvement

Digital PVAS10 score (pruritus severity) was not significantly correlated with GM PCoA scores along the first three axes in either group at either time point (*p* > 0.05, Spearman’s correlation). To further examine the association between pruritus improvement and the change in the GM abundance at the species level, subjects in each intervention group were further ranked and divided into tertiles based on their magnitude of pruritus score improvement at week 10 relative to baseline. The pruritus response groups were defined as high responder (HR, most % PVAS10 improved, PBO: 91% to 41% decrease, PNB: 92% to 55% decrease); midresponder (MR, PBO: 38% to 5% decrease, PNB: 41% to 2% decrease); and low responder (LR, least % PVAS10 improved or worsened, PBO: 1% decrease to 67% increase, PNB: 2% decrease to 77% increase).

Even without any biologically meaningful intervention, subjects in the PBO group still showed a wide range of changes in pruritus scores at week 10 (Figure S5). LRs (*n* = 8) showed an increase of 21 species, 12 of which were in the Proteobacteria phylum (Figure S20, Table S10). On the other hand, HRs (*n* = 8) showed an increase in 13 species, eight of which were in the Firmicutes_A phylum, as well as a decrease in 20 species, 17 of which were in the Firmicutes phylum (Figure S21, Table S11). Interestingly, two of the six probiotics species (included in the PNB formulation) were among the species that showed a decrease in their abundances (*B. animalis* and *L. rhamnosus*) in HRs.

In the PNB group, the patterns of abundance change were different between HRs (*n* = 9) and LRs (*n* = 9). While the abundances of 14 species in the Firmicutes phylum decreased at week 10 in HRs (Figure S22, Table S12), the abundances of 12 species in the Proteobacteria phylum decreased at week 10 in LRs (Figure S23, Table S13). Additionally, the abundances of 40 species increased at week 10 in LRs, 19 of which were in the Firmicutes_A phylum and 14 of which were in the Firmicutes phylum. Examining the six probiotics species in the PNB supplement, increases in four species in LRs were observed (*B. animalis*, *L. casei*, *L. rhamnosus*, and *L. acidophilus*), while only one species increased in HRs (*L. rhamnosus*).

To examine GM differences in subjects whose pruritus severity most improved in the PNB group, the GM abundance at baseline was compared between HRs and LRs (Figure S24, Table S14). HRs had lower abundances of 29 species than LRs at baseline, 16 of which were in the Proteobacteria phylum. HRs also had 59 species with higher abundances than LRs at baseline, 22 of which were in the Bacteroidota phylum and 21 of which were in the Firmicutes_A phylum. Interestingly, two of the six probiotics species included in the PNB formulation were found to have higher abundances in HRs at baseline (*B. animalis* and *B. infantis*). None of the species in the Proteobacteria phylum had higher abundance in HRs than LRs, and none of the species in the Bacteroidota phylum had higher abundance in LRs than HRs. Evenness and Shannon H, but not richness, were significantly different among HRs, MRs, and LRs at baseline in the PNB group (Figure S25). Post hoc comparisons demonstrated that the difference was between HRs and LRs (evenness: adjusted *p* = 0.041; Shannon H: adjusted *p* = 0.057).

The same analysis was done in the PBO group (Figure S26, Table S15). HRs had abundances of 38 species higher than LRs at baseline, 20 of which belonged to the Firmicutes phylum. Fifty-five species were also found to be lower in HRs than LRs at baseline in the PBO group, 21 of which were in the Proteobacteria phylum. The α-diversity metrics were not significantly different among HRs, MRs, and LRs at baseline in the PBO group (Figure S25).

## 4. Discussion

The goal of this randomized controlled trial was to evaluate the effect of a probiotic and nutraceutical blend (PNB) on pruritic dermatitis (PD) and fecal microbiota in dogs on nutritionally complete diets. The PNB was found to offer some support for clinical signs of pruritus and skin allergy, as measured by digital PVAS10, OA-SASI, and skin redness score, earlier than the placebo (PBO). While not statistically significant, the majority of additional health, skin and coat, and general wellness measures were more favorable for the PNB group at the end of the trial. Additionally, more dogs in the PNB group shifted to the very mild-to-normal digital PVAS10 range (digital PVAS10-N: <2) by week 4, as opposed to week 7 for the PBO. The differences in the clinical outcomes are further supported by the gut microbiome (GM) findings which were characterized by shotgun metagenomic sequencing. At the end of the trial, three supplemented probiotics were enriched in the PNB group while pathogenic species had reduced abundances. Meanwhile, dogs in the PBO group saw an increase in the abundance of Proteobacteria and a reduction in several beneficial species including one probiotic in the PNB formulation [60,62,63]. Collectively, the evidence demonstrates that the PNB may serve as an additive therapy for PD via multimodal actions, which is important given the diversity in triggers that can lead to differences in clinical severity and treatment response [3,22]. Traditional therapies for pruritic skin diseases are often associated with side effects [5,11], not to mention varied or incomplete responses [3,89]. Additional synergistic therapies may reduce the dose or frequency of medication to help minimize side effects [5,63,90,91] or offer a proactive or alternative therapy to those with poor or no response [30].

Several ingredients utilized in this trial (especially vitamin E and B vitamins) and probiotic strains (particularly *Lactobacillus* strains) have been examined alone or in fortified dermatological diets for their ability to impact canine skin allergies, from which the doses used in this study were extrapolated [21,29,32,60,61,62,63,65,92]. However, the majority of ingredients utilized in this trial were selected for their ability to benefit a spectrum of relevant factors, including barrier function [25,30], immune parameters [37,45,66,93], and markers of oxidative stress [35,36]. To our knowledge this is the first time this combination of ingredients has been examined together. One other study explored nutraceutical supplementation, which included tyndallized *Lactobacillus reuteri*, on skin allergy symptoms and the gut microbiota in household atopic dogs; however, the ingredients of the supplement in this 120-day pre–post study did not overlap with those examined herein and degree of dysbiosis was the sole microbiomic outcome [49]. Nevertheless, both trials complemented each other in showcasing that a probiotic and nutraceutical-containing supplement given as an additive therapy may have beneficial impacts on PD and the GM [49].

The canine pruritus severity scale (digital PVAS10), chosen as a primary outcome, was developed for owner-assessment and has been extensively verified [74,75]. At each assessment the owner’s previous digital PVAS10 evaluation was prepopulated, as this has been recently demonstrated to lead to better agreement between owners’ perceptions and changes in pruritus scores and is believed to improve scoring reliability and effectiveness [94]. While other studies regularly employ use of this scale [49,63,64,95,96,97,98,99,100], to our knowledge none have shown the score from the previous assessment, and, more commonly, less reliable versions of visual analogue scales or numerical scales are employed [75,101]. Given that dogs representing mild pruritus (digital PVAS10 < 3.6) were enrolled, an appropriate outcome threshold is normal to very mild pruritus (digital PVAS10-N: <2) [76]. At the end of the trial 30.30% of dogs on the PNB were in this normal pruritus range, as compared to 20.69% on the PBO. While not significantly different, according to Rybnícek et al. the number of dogs that end in this range is an important indicator of clinical improvement regardless of statistical significance [75].

As an additional readout, this trial developed the Owner Assessed-Skin Allergy Severity Index (OA-SASI), a skin lesion severity assessment. Similar to other pruritus outcome measures, the OA-SASI scale showed a difference between groups at week 2 but also offered further precision insights into the body sites and lesion types driving this difference. As described earlier, this scale was derived from the Canine Atopic Dermatitis Extent and Severity Index (CADESI)-4. Significant modifications to the traditional scale simplified it for owner-assessment while preserving principal components. Other studies have modified earlier CADESI versions and demonstrated clear improvements in privately owned atopic dogs [32,102,103,104,105]. We intentionally transformed the fourth version of the CADESI for owner use because it was designed to be more straightforward and efficient than preceding versions [77]. However, we recognize that the results cannot be compared to studies utilizing the traditional scale, and by using owners instead of a single clinician reliability of scoring may have been reduced [106].

While much is still unknown about the skin–gut axis, especially in canines, the relationship is slowly being elucidated [19,20,30,107], as well as the role of the GM in the pathogenesis of pruritic skin diseases [19,20,47,49]. It has been speculated that allergic skin diseases may present as a result of gut dysbiosis and inflammation [19]. Newer research suggests that fecal microbiota transplantation, provided as an early intervention to at-risk dogs, may reduce development of canine atopic dermatitis (CAD) by 18 months of age [108], and further fecal microbiota transplantation capsules have been shown to improve pruritus, barrier function, and gut microbiome diversity in preliminary research in adult atopic dogs [109]. Additionally, healthy dogs have also been shown to have distinctive fecal microbiome profiles as compared to diseased populations [110]. This has been recently demonstrated specifically with atopic dogs as well, with CAD being associated with gut dysbiosis [20,52] and lower diversity than healthy controls [20], which confirms the existing research in humans [50,51,111,112]. In previous studies in healthy dogs on the same diets used in this study we reported that Firmicutes, Proteobacteria, and Bacteroidota were the predominant phyla [53,71]. However, compared to the healthy dogs in those studies, dogs in this present trial had lower abundances of both Actinobacteria and Firmicutes. These dissimilarities could be attributed to the impact of PD, but more research is needed to confirm this observation.

PNB supplementation led to significant increases in three supplemented probiotic species; however, there was an overall trend of an increase in all six probiotics included in the formulation. Of these, *L. rhamnosus* has been found to decrease allergen-specific IgE in puppies predisposed to atopy [60] and appeared to have lasting effects on immunological indicators [61]. Other *Lactobacillus* species supported some reduction in skin allergy symptoms in 8-week and 12-week double-blind randomized controlled trials [62,63] as well as a 90-day pre–post study [65]. *Lactobacillus* and *Bifidobacterium* species have also demonstrated immunomodulatory properties in allergy mouse models [113,114].

Over the course of the study, dogs on the PNB saw a stark decrease in the abundance of species in the Proteobacteria phylum, some of which are known pathogens in both dogs and humans, including *P. mirabilis* [115], *C. freundii* [116], and *K. pneumoniae* [117,118], as well as a decrease in other presumed canine pathogens such as *Enterococcus avium* [119]. On the contrary, increased abundances of possibly pathogenic Proteobacteria species [117] were observed in the PBO group. A larger increase in beneficial species in the Firmicutes phylum was observed in the PNB group, while several species from this phylum decreased in the PBO group. More importantly, however, in the PNB group an increase in short-chain fatty acid (SCFA) producing bacteria in the *Lachnospiraceae* family was observed, which may improve intestinal barrier integrity [120], and atopic dogs have been found to have lower abundances compared to healthy counterparts [20]. Dogs with PNB supplementation were also found to have increased fecal abundances of *Weissella cibaria* at week 10, particularly in the high-responder group (HR, most % improved pruritus response), which also saw increased abundances of *W. confusa*, while dogs in the PBO group had decreased abundances of *W. cibaria*. Emerging research highlights the potentially beneficial role of *Weissella* species in reducing and treating skin conditions and allergic diseases [121,122]. However, the clinical significance of these shifts remains unknown. While Proteobacteria levels presented in this study may appear elevated [123], they are more comparable to the levels from previous research in pruritic dogs [20], especially household populations [124,125]. However, this study employs shotgun metagenomic sequencing which offers a more comprehensive analysis of the microbiome than the more widely used 16S amplicon sequencing [123]. Additionally, there are a number of potential sources of bias in microbiome studies, a known limitation of the field, which can make comparing across studies complex.

Differences between the baseline GM profiles of HRs and LRs (based on relative pruritus score improvement) were observed via subgroup analysis. In the PNB group, the LR subgroup had higher abundances of several possibly pathogenic species in the Proteobacteria phylum, as well as other suspected pathogens including *Corynebacterium mustelae* [126], *Streptococcus pasteurianus* [127], *Buchananella hordeovulneris* [128], and *Emergencia timonensis* [129]. However, at baseline the HR subgroup in the PNB group had higher abundances of two supplemented probiotic species, as well as species in the Bacteroidales order and Lachnospirales order, both of which are major bacterial inhabitants of the canine GM community [120,130], and lower abundances of species in both orders have been associated with atopic dermatitis in humans [131,132,133]. In the PNB group, the HRs also had higher evenness and Shannon diversity than LRs at baseline, which has been demonstrated to be associated with better health across a variety of indications [120,134,135,136]. At baseline in the PBO group, HRs had higher abundances of several species in the Firmicutes phylum and lower abundances of possibly pathogenic species in the Proteobacteria phylum compared to LRs. Taken together, these observations potentially indicate that the less ideal baseline GM seen in LRs may have contributed to their lower pruritus response, and HRs may have already been on a trajectory of improvement. Additionally, the magnitude of PCoA1 shift was found to be associated with baseline PCoA1 score and α-diversity, and there were distinct differences in the baseline GMs between subjects in the first and third tertiles (based on the β-diversity shift along the PCoA1 axis) in the PNB group. This further indicates that changes seen in the GM may be based on the baseline profiles and can potentially be utilized as a predictive measure of response [53,71,137]. Curiously, at the end of the trial, an increase in four of the six probiotic species included in the formulation was observed in LRs in the PNB group, while an increase in only one probiotic species was observed in HRs. In the PBO group, decreases in two of the supplemented probiotic species were observed in HRs and no changes in the levels of the probiotic species were observed in LRs at week 10. These findings indicate that an increase in probiotic species was not an indication of better pruritus improvement within the 10-week period, which may have been driven by the other ingredients included in the PNB supplement or other biological pathways unrelated to GM. Interestingly, a decrease in species in the Firmicutes phylum at week 10 may have been associated with improved pruritus, as this was seen in HRs in both the PNB and PBO groups, and was not present to the same extent in LRs.

Dogs in the PBO group were found to have significant improvements in clinical signs of pruritus and changes in their GM profile. In canine trials there is a limited recognition of the placebo effect [138,139]. According to Muñana et al. the “perceived placebo effect” describes when clinical improvements in the placebo group lead to the perception that improved condition is associated with the placebo supplement [139]. Factors that likely contributed to the “perceived placebo effect” seen in this trial include improved monitoring and care by the owner, the passing of time, and the seasonality of allergy symptoms [139,140,141]. The presence of the placebo effect may mask some of the actual improvements seen with the PNB, while simultaneously diminishing the true differences between the two intervention groups. Inclusion of the PBO group also provided the opportunity to examine the temporal change of the GM in pruritic dogs without any biologically meaningful intervention. Based on the changes in the GM composition detailed for both intervention groups, changes in the PNB group further support the pruritus and clinical improvements observed, while the pruritus changes in the PBO group may be attributed to a placebo effect or reflect the underlying dynamic nature of the GM in this population. Simultaneously, we also recognize the possibility that maltodextrin is not truly inert and could affect the GM [142,143].

While the dogs included in this trial were found to have PD, given the applied screening criteria, we believe the majority of dogs were likely to have CAD; however, no formal clinical diagnosis was made. The definitive diagnosis of CAD is difficult considering its similarities to other pruritic skin diseases and its range of clinical presentation and severity [1,3]. Given the disease’s complexity, it is gaining recognition as a clinical syndrome [144]. The International Committee for Allergic Diseases in Animals (ICADA) has outlined three ways in which a CAD diagnosis can be made by trained practitioners: (1) elimination of similar skin conditions through formal examination and work-up; (2) interpretation of clinical history and features based on diagnostic criteria sets; and (3) allergy testing for confirmation and allergen identification [1]. Multiple diagnostic criteria sets exist to aid in the interpretation of clinical features associated with CAD; however, Brément et al. compared Willemse [145], Prélaud [146], and Favrot (both set 1 and set 2) [88] and demonstrated that when used in isolation all criteria sets were unreliable [147]. Thus, it is recommended that all three diagnostic approaches outlined by ICADA are used in combination for a more accurate assessment, which was not possible in the present investigation [1,11,147].

In this trial, eligibility was based on an owner-reported online screening survey (Figure S1). While we acknowledge that a definitive diagnosis of CAD cannot be made using our criteria, and thus have determined enrolled dogs to have PD, dogs with features consistent with a CAD diagnosis were targeted. Elimination of similar pruritic diseases to CAD was achieved through focused questions, owner reports of medical history, or indication of a CAD diagnosis by a veterinarian. While the questionnaire was not validated, another trial previously demonstrated that owner-reported survey information could serve as a useful diagnostic tool for CAD [148]. To assess the clinical features and history of CAD, we did not strictly adhere to any particular criteria set, but rather relied on owner indication of compatible features including presence of widespread or localized pruritus, licking, biting, chewing, hair loss, skin irritation, dry or oily skin, or a dull coat. Given our multiple diagnostic approaches employed, baseline clinical signs of allergy being comparable to another trial with owner-reported clinical signs of CAD [148], and CAD being one of the major causes of PD [1], we feel that a large proportion of the dogs included in this trial were atopic, and thus designed the PNB and primary outcomes based on this assumption.

However, we also intentionally deviated from stricter criteria in a few instances and this may have excluded some dogs from receiving a formal CAD diagnosis under direct veterinary evaluation. Firstly, we did not exclude dogs that reported the onset of allergic symptoms post three years of age, as it is still prevalent [5,148], despite Favrot’s criteria suggesting the age of onset of CAD as <3 years old [1,88]. We did, however, perform a subgroup analysis of subjects with symptom onset between the ages of 1 and 3 years, which demonstrated more pronounced improvements in the PNB group (Figure S7), suggesting possibly greater benefits of the intervention under more defined criteria. Secondly, we enrolled dogs with seasonal symptoms (29% included in the survey analysis); however, current recommendations suggest enrolling dogs with only nonseasonal symptoms [76]. Indeed, a subgroup analysis of dogs with nonseasonal PD showed more pronounced improvements associated with PNB supplementation (Figure S8). Lastly, at the start of the trial, the enrolled dogs had a wide range of pruritus severity, with digital PVAS10 scores ranging from very mild to severe [76]; however, it is typically recommended to recruit dogs in the moderate-to-severe range (digital PVAS10 ≥ 3.6) [76]. While the vast majority of dogs in this trial did fall in that range (*n* = 60), we included two dogs representing mild pruritus (digital PVAS10 < 3.6) because mild scores are still representative of PD [75] and the owners indicated that the dogs had visibly pruritic and irritated skin requiring treatment. Owner perception and quality of life are important considerations that cannot be overlooked in trials utilizing client-owned dogs [76,149,150]. Further, by including dogs that would not necessarily meet stricter diagnostic criteria, the results may be more generalizable to the population of dogs with chronic pruritic dermatitis regardless of cause. That being said, given that the absolute change at week 10 in digital PVAS10 score and OA-SASI score (log-transformed) were both significantly correlated with the baseline score in the PNB group only (Figure 2b and Figure 3b), with a larger change corresponding to a higher baseline score, this indicates that PNB may be more effective in dogs with more severe cases of PD.

Beyond diagnostic considerations and the use of the owner-reported data discussed above, additional limitations are worth considering. The present study did not contain any follow-up, so it is not known whether the observed benefits were sustained, and to what degree, after the supplementation period. Our previous research has demonstrated that changes to the GM (abundance and β-diversity) in healthy dogs returned to baseline two weeks after stopping synbiotic supplementation [53]. Likewise, without mid-trial GM samples it is unclear how rapidly any changes in the GM occurred and whether they were associated with clinically relevant changes. Future studies would benefit from biological and functional markers and the assessment of barrier function to provide more comprehensive measures of impact. Ideally, they should be conducted in a more controlled environment with assessment by a single clinician. Skin microbiome samples would also be of value, as maintaining the biodiversity of the skin is of key importance because as pruritic diseases are associated with dysbiosis and lower species richness and diversity [151,152,153,154].

While direct-to-consumer enrollment served to easily standardize diet type, treat intake and dietary variability among the four fresh canine diet recipes consumed may still have impacted the response to the supplementation. Notably polyunsaturated fatty acid content is not the same among all recipes, however, all diets share similar digestibility and exceed AAFCO requirements for crude protein and fat [72]. That being said, due to our randomized design we can assume a similar distribution of the four recipes between groups. Seasonality could have also affected the severity of clinical signs. Another trial in pruritic client-owned dogs reported that seasonal symptoms were most severe during the summer months [148]. In our trial, 73% of dogs included in the survey analysis completed the study from June through August, and participating dogs were from different regions of the continental US (Table 1), which could have led to variability in environmental triggers [6,155,156]. Future trials could consider seasonality by implementing a set start date, extending the length of the trial to encompass all seasons, or only including dogs with nonseasonal symptoms. Finally, although we observed differences at baseline among overall health, hours spent outside, and scratching amount, these differences were not correlated with pruritus severity and are likely attributable to the sizable number of variables examined within the same population, which was not specifically adjusted for these more generic secondary health outcomes. Given the variety and complexity of pruritic diseases, current treatment recommendations advocate for bespoke and dynamic treatment plans based on current clinical assessments [5,24,157]. Therefore, it is possible that even with the significant improvements seen with PNB supplementation, some dogs may have been less responsive to the treatment given their individualized needs.

## 5. Conclusions

In conclusion, this 10-week randomized double-blind placebo-controlled trial highlighted interesting clinical and microbiomic changes in client-owned dogs with pruritic dermatitis (PD) receiving a probiotic and nutraceutical blend (PNB). Dogs within the trial had varying degrees of clinical severity, as well as representing a span of breeds and ages, expanding the applicability of these results to the general pet population. PNB administration supported faster improvements and resolution in PD severity and erythema, while simultaneously enriching the gut microbiome (GM) with three of six supplemented probiotics, while reducing species commonly associated with an unhealthy GM. Results of this trial suggest that improvements in clinical signs of skin allergy may be seen after two weeks of supplementation and sustained through 10 weeks, with changes in the GM observed at week 10. This study is among the first to examine a multistrain probiotic and nutraceutical supplement in pruritic dogs.

## Figures and Tables

**Table 1:** Subject characteristics.

	PBO (*n* = 29)	PNB (*n* = 33)	*p* Value *
Age (years)	7.60 ± 3.5	7.51 ± 3.3	0.882
Female	16 (55%)	16 (48%)	0.621
Spayed or neutered	29 (100%)	32 (97%)	1.000
BCS ^a^			1.000
4–5	25 (86%)	28 (85%)	
6	4 (14%)	5 (15%)	
Current body weight (kg)	10.0 ± 6.5	8.9 ± 5.5	0.507
Hours Outside (per day)			**0.038**
≤1 h	13 (45%)	24 (73%)	
>1 h	16 (55%)	9 (27%)	
Fecal Score ^b^	3.0 ± 0.8	2.9 ± 1.0	0.622
Coat Style			0.667
Short-coated	11 (38%)	12 (36%)	
Long-coated	3 (10%)	4 (12%)	
Curly-coated	4 (14%)	9 (27%)	
Medium-coated	10 (34%)	7 (21%)	
Wire-coated	1 (4%)	1 (4%)	
Current US Region ^c^			0.646
North	4 (14%)	8 (24%)	
South	11 (38%)	12 (36%)	
West	10 (34%)	11 (33%)	
Midwest	4 (14%)	2 (7%)	
Uses Allergy specific medication ^d^	18 (62%)	21 (64%)	1.000
Uses Flea medication	16 (55%)	17 (52%)	0.804
Allergy Description			
Associated with diarrhea	1 (4%)	0 (0%)	0.468
Associated with food	2 (7%)	3 (9%)	1.000
Mostly involves back	2 (7%)	5 (15%)	0.433
Mostly involves belly/armpits	11 (38%)	12 (36%)	1.000
Mostly involves face	8 (28%)	7 (21%)	0.767
Mostly involves ears	11 (38%)	8 (24%)	0.280
Mostly involves feet	18 (62%)	20 (61%)	1.000
Occurs all over body	4 (14%)	2 (6%)	0.405
Seasonal symptoms	11 (38%)	7 (21%)	0.171

Data are expressed as mean ± SD or *n* (%). * Wilcoxon rank-sum test for continuous variables and Fisher’s exact test for categorical variables. ^a^ 9-point scale; Body Condition Score (BCS): 4–5 (ideal weight), 6 (slightly overweight). ^b^ Bristol Stool Form Scale; regular scores 2–5 (ideal 3–4). ^c^ Regions (Continental US): North—ME, NH, VT, MA, RI, CT, NY, NJ, PA, and DE; South—MD, WV, VA, KY, NC, SC, TN, GA, FL, AL, MS, LA, AS, OK, and TX; Midwest—MO, KS, NE, SD, ND, MN, IA, WI, IL, IN, MI, and OH; West—NM, CO, WY, MT, ID, UT, AZ, NV, WA, OR, and CA. ^d^ Including steroids, antihistamines, immunosuppressants, oclacitinib, lokivetmab and allergen immunotherapy, sublingual immunotherapy, topicals, and medicated hypoallergenic shampoos and wipes. Was bolded to signify *p* < 0.05.

**Table 2:** Digital PVAS10 severity thresholds.

		Severe	Moderate	Mild	Normal	*p* Value *
		Digital PVAS10: ≥5.6	Digital PVAS10: 3.6–5.5	Digital PVAS10: 2–3.5	Digital PVAS10-N: <2	
Week 0	PBO (*n* = 29)	24 (83%)	5 (17%)	0 (0%)	0 (0%)	1.000
	PNB (*n* = 33)	26 (79%)	5 (15%)	1 (3%)	1 (3%)	
Week 2	PBO (*n* = 29)	18 (62%)	8 (28%)	1 (3%)	2 (7%)	0.264
	PNB (*n* = 33)	12 (36.5%)	12 (36.5%)	3 (9%)	6 (18%)	
Week 4	PBO (*n* = 29)	10 (34.5%)	12 (41.5%)	3 (10%)	4 (14%)	0.519
	PNB (*n* = 33)	13 (39.5%)	8 (24.5%)	5 (15%)	7 (21%)	
Week 7	PBO (*n* = 29)	10 (34.5%)	9 (31%)	2 (6.5%)	8 (28%)	1.000
	PNB (*n* = 33)	11 (34%)	7 (21%)	5 (15%)	10 (30%)	
Week 10	PBO (*n* = 29)	10 (34.5%)	10 (34.5%)	3 (10%)	6 (21%)	0.562
	PNB (*n* = 33)	12 (36.5%)	8 (24.5%)	3 (9%)	10 (30%)	

* Fisher’s exact test.

**Table 3:** Species with differential abundances between baseline and week 10 in PNB (*n* = 27, log2|fold change| ≥ 2 and FDR-adjusted *p* < 0.05).

Phylum	Class	Order	Family	Genus	Species	Relative Abundance (%) Median [IQR]		Week 10 vs. Baseline	
						Baseline	Week 10	Log2 FC Mean ± SE	Adjusted *p*-Value ^1^
Increased at week 10 (15 species)									
Firmicutes	Bacilli	Lactobacillales	Lactobacillaceae	Leuconostoc	kimchii	0.00 × 10^+0^ [0.00 × 10^+0^–0.00 × 10^+0^]	0.00 × 10^+0^ [0.00 × 10^+0^–0.00 × 10^+0^]	11.89 ± 3.96	2.57 × 10^−2^
Firmicutes	Bacilli	Lactobacillales	Lactobacillaceae	Lacticaseibacillus	rhamnosus	0.00 × 10^+0^ [0.00 × 10^+0^–1.19 × 10^−6^]	3.40 × 10^−6^ [4.57 × 10^−7^–2.69 × 10^−3^]	9.07 ± 0.99	4.32 × 10^−17^
Actinobacteriota	Actinomycetia	Actinomycetales	Bifidobacteriaceae	Bifidobacterium	animalis	0.00 × 10^+0^ [0.00 × 10^+0^–1.02 × 10^−7^]	0.00 × 10^+0^ [0.00 × 10^+0^–8.49 × 10^−4^]	8.45 ± 2.40	8.54 × 10^−3^
Firmicutes	Bacilli	Lactobacillales	Lactobacillaceae	Leuconostoc	carnosum	0.00 × 10^+0^ [0.00 × 10^+0^–0.00 × 10^+0^]	0.00 × 10^+0^ [0.00 × 10^+0^–1.41 × 10^−6^]	8.04 ± 2.75	3.07 × 10^−2^
Actinobacteriota	Actinomycetia	Actinomycetales	Bifidobacteriaceae	Bifidobacterium	unknown	0.00 × 10^+0^ [0.00 × 10^+0^–1.34 × 10^−7^]	4.09 × 10^−7^ [0.00 × 10^+0^–4.28 × 10^−5^]	7.56 ± 2.11	8.16 × 10^−3^
Firmicutes	Bacilli	Lactobacillales	Lactobacillaceae	Weissella	cibaria	0.00 × 10^+0^ [0.00 × 10^+0^–0.00 × 10^+0^]	1.87 × 10^−7^ [0.00 × 10^+0^–7.83 × 10^−6^]	7.12 ± 1.32	1.90 × 10^−5^
Actinobacteriota	Actinomycetia	Actinomycetales	Bifidobacteriaceae	Bifidobacterium	longum	0.00 × 10^+0^ [0.00 × 10^+0^–0.00 × 10^+0^]	4.09 × 10^−7^ [0.00 × 10^+0^–3.54 × 10^−6^]	7.08 ± 2.00	8.31 × 10^−3^
Firmicutes	Bacilli	Lactobacillales	Lactobacillaceae	Lactobacillus	unknown	0.00 × 10^+0^ [0.00 × 10^+0^–7.78 × 10^−8^]	0.00 × 10^+0^ [0.00 × 10^+0^–8.45 × 10^−6^]	6.05 ± 1.31	2.52 × 10^−4^
Firmicutes_A	Clostridia	Oscillospirales	Oscillospiraceae	Flavonifractor	unknown	0.00 × 10^+0^ [0.00 × 10^+0^–1.40 × 10^−6^]	0.00 × 10^+0^ [0.00 × 10^+0^–8.78 × 10^−5^]	5.34 ± 1.88	3.57 × 10^−2^
Firmicutes	Bacilli	Lactobacillales	Lactobacillaceae	Lacticaseibacillus	unknown	0.00 × 10^+0^ [0.00 × 10^+0^–7.78 × 10^−7^]	3.74 × 10^−6^ [1.95 × 10^−7^–1.12 × 10^−4^]	5.09 ± 1.06	1.53 × 10^−4^
Firmicutes	Bacilli	Lactobacillales	Streptococcaceae	Lactococcus	garvieae	0.00 × 10^+0^ [0.00 × 10^+0^–0.00 × 10^+0^]	0.00 × 10^+0^ [0.00 × 10^+0^–3.28 × 10^−6^]	4.59 ± 1.17	2.80 × 10^−3^
Actinobacteriota	Coriobacteriia	Coriobacteriales	Coriobacteriaceae	Collinsella	phocaeensis	0.00 × 10^+0^ [0.00 × 10^+0^–1.76 × 10^−5^]	0.00 × 10^+0^ [0.00 × 10^+0^–1.56 × 10^−4^]	4.02 ± 1.40	3.45 × 10^−2^
Firmicutes_A	Clostridia	Lachnospirales	Lachnospiraceae	Blautia_A	sp000433815	2.65 × 10^−6^ [3.99 × 10^−7^–6.95 × 10^−5^]	4.59 × 10^−5^ [7.21 × 10^−6^–4.53 × 10^−4^]	2.54 ± 0.62	1.73 × 10^−3^
Firmicutes	Bacilli	Lactobacillales	Lactobacillaceae	Lactobacillus	acidophilus	1.55 × 10^−6^ [4.27 × 10^−7^–3.29 × 10^−6^]	5.05 × 10^−6^ [8.73 × 10^−7^–3.89 × 10^−4^]	2.30 ± 0.70	1.31 × 10^−2^
Firmicutes_A	Clostridia	Lachnospirales	Lachnospiraceae	Ruminococcus_A	sp000432335	1.35 × 10^−5^ [1.34 × 10^−6^–6.17 × 10^−5^]	5.09 × 10^−5^ [7.29 × 10^−6^–3.15 × 10^−4^]	2.05 ± 0.60	1.04 × 10^−2^
Decreased at week 10 (38 species)									
Proteobacteria	Gammaproteobacteria	Enterobacterales	Enterobacteriaceae	Kosakonia	cowanii	0.00 × 10^+0^ [0.00 × 10^+0^–9.15 × 10^−7^]	0.00 × 10^+0^ [0.00 × 10^+0^–0.00 × 10^+0^]	−7.84 ± 2.30	1.11 × 10^−2^
Proteobacteria	Gammaproteobacteria	Enterobacterales	Enterobacteriaceae	Kluyvera	cryocrescens	2.43 × 10^−7^ [0.00 × 10^+0^–1.06 × 10^−6^]	0.00 × 10^+0^ [0.00 × 10^+0^–9.36 × 10^−8^]	−5.55 ± 1.40	2.77 × 10^−3^
Proteobacteria	Gammaproteobacteria	Enterobacterales	Enterobacteriaceae	Providencia	rettgeri_D	2.66 × 10^−7^ [0.00 × 10^+0^–3.05 × 10^−6^]	0.00 × 10^+0^ [0.00 × 10^+0^–8.66 × 10^−7^]	−5.49 ± 1.94	3.57 × 10^−2^
Firmicutes	Bacilli	Lactobacillales	Vagococcaceae	Vagococcus	fluvialis_A	0.00 × 10^+0^ [0.00 × 10^+0^–3.99 × 10^−6^]	0.00 × 10^+0^ [0.00 × 10^+0^–3.44 × 10^−7^]	−4.87 ± 1.72	3.57 × 10^−2^
Proteobacteria	Gammaproteobacteria	Enterobacterales	Enterobacteriaceae	Proteus	unknown	2.67 × 10^−6^ [0.00 × 10^+0^–8.42 × 10^−5^]	0.00 × 10^+0^ [0.00 × 10^+0^–7.36 × 10^−5^]	−4.82 ± 1.73	3.74 × 10^−2^
Proteobacteria	Gammaproteobacteria	Enterobacterales	Enterobacteriaceae	Proteus	mirabilis	3.24 × 10^−4^ [7.18 × 10^−6^–1.01 × 10^−2^]	5.01 × 10^−6^ [3.69 × 10^−7^–4.25 × 10^−3^]	−4.27 ± 0.84	6.96 × 10^−5^
Proteobacteria	Gammaproteobacteria	Enterobacterales	Enterobacteriaceae	Kluyvera	ascorbata	0.00 × 10^+0^ [0.00 × 10^+0^–5.80 × 10^−7^]	0.00 × 10^+0^ [0.00 × 10^+0^–2.35 × 10^−7^]	−3.93 ± 1.29	2.24 × 10^−2^
Firmicutes	Bacilli	Lactobacillales	Enterococcaceae	Enterococcus_A	avium	8.57 × 10^−4^ [3.54 × 10^−6^–7.50 × 10^−3^]	1.90 × 10^−5^ [8.49 × 10^−7^–5.11 × 10^−4^]	−3.71 ± 0.76	1.20 × 10^−4^
Proteobacteria	Gammaproteobacteria	Enterobacterales	Enterobacteriaceae	Klebsiella	quasivariicola	2.43 × 10^−7^ [0.00 × 10^+0^–5.51 × 10^−6^]	0.00 × 10^+0^ [0.00 × 10^+0^–1.09 × 10^−6^]	−3.62 ± 1.13	1.53 × 10^−2^
Firmicutes	Bacilli	Lactobacillales	Streptococcaceae	Streptococcus	equi	0.00 × 10^+0^ [0.00 × 10^+0^–1.71 × 10^−6^]	0.00 × 10^+0^ [0.00 × 10^+0^–6.14 × 10^−7^]	−3.29 ± 1.15	3.48 × 10^−2^
Proteobacteria	Gammaproteobacteria	Enterobacterales	Enterobacteriaceae	Cronobacter	malonaticus	2.25 × 10^−6^ [0.00 × 10^+0^–6.97 × 10^−6^]	2.88 × 10^−7^ [0.00 × 10^+0^–3.07 × 10^−6^]	−3.27 ± 0.83	2.80 × 10^−3^
Firmicutes	Bacilli	Lactobacillales	Streptococcaceae	Streptococcus	orisratti	0.00 × 10^+0^ [0.00 × 10^+0^–4.27 × 10^−7^]	0.00 × 10^+0^ [0.00 × 10^+0^–4.55 × 10^−7^]	−3.19 ± 1.14	3.74 × 10^−2^
Proteobacteria	Gammaproteobacteria	Enterobacterales	Enterobacteriaceae	Enterobacter	hormaechei	5.52 × 10^−7^ [0.00 × 10^+0^–2.20 × 10^−6^]	0.00 × 10^+0^ [0.00 × 10^+0^–7.67 × 10^−7^]	−3.14 ± 0.97	1.43 × 10^−2^
Proteobacteria	Gammaproteobacteria	Enterobacterales	Enterobacteriaceae	Phytobacter	diazotrophicus	0.00 × 10^+0^ [0.00 × 10^+0^–1.62 × 10^−6^]	0.00 × 10^+0^ [0.00 × 10^+0^–1.05 × 10^−6^]	−3.12 ± 1.16	4.41 × 10^−2^
Proteobacteria	Gammaproteobacteria	Enterobacterales	Enterobacteriaceae	Yersinia	pestis	7.18 × 10^−5^ [1.17 × 10^−5^–1.37 × 10^−4^]	9.73 × 10^−6^ [1.70 × 10^−6^–1.14 × 10^−4^]	−3.11 ± 0.67	2.52 × 10^−4^
Proteobacteria	Gammaproteobacteria	Enterobacterales	Enterobacteriaceae	Klebsiella_A	unknown	2.14 × 10^−6^ [2.68 × 10^−7^–8.37 × 10^−6^]	6.82 × 10^−7^ [0.00 × 10^+0^–3.53 × 10^−6^]	−2.88 ± 0.81	8.31 × 10^−3^
Firmicutes	Bacilli	Haloplasmatales	Turicibacteraceae	Turicibacter	sp001543345	1.44 × 10^−5^ [1.46 × 10^−6^–2.72 × 10^−4^]	1.05 × 10^−5^ [4.88 × 10^−7^–3.32 × 10^−5^]	−2.68 ± 0.63	1.05 × 10^−3^
Firmicutes_A	Clostridia	Clostridiales	Clostridiaceae	Clostridium	saudiense	1.06 × 10^−5^ [1.11 × 10^−6^–5.82 × 10^−5^]	3.07 × 10^−6^ [1.63 × 10^−7^–1.42 × 10^−5^]	−2.66 ± 0.64	1.73 × 10^−3^
Firmicutes_A	Clostridia	Clostridiales	Clostridiaceae	Clostridium	sp000753455	1.55 × 10^−6^ [1.79 × 10^−7^–2.01 × 10^−5^]	5.34 × 10^−7^ [0.00 × 10^+0^–1.94 × 10^−6^]	−2.64 ± 0.66	2.54 × 10^−3^
Proteobacteria	Gammaproteobacteria	Enterobacterales	Enterobacteriaceae	Citrobacter_A	rodentium	1.58 × 10^−5^ [1.33 × 10^−6^–5.33 × 10^−5^]	6.79 × 10^−6^ [1.09 × 10^−6^–1.46 × 10^−5^]	−2.64 ± 0.69	3.78 × 10^−3^
Proteobacteria	unknown	unknown	unknown	unknown	unknown	8.51 × 10^−6^ [4.13 × 10^−6^–2.41 × 10^−5^]	5.34 × 10^−6^ [1.26 × 10^−6^–1.36 × 10^−5^]	−2.61 ± 0.56	2.52 × 10^−4^
Firmicutes	Bacilli	Lactobacillales	Streptococcaceae	Lactococcus	piscium_C	6.28 × 10^−7^ [0.00 × 10^+0^–1.86 × 10^−6^]	1.88 × 10^−7^ [0.00 × 10^+0^–1.51 × 10^−6^]	−2.58 ± 0.72	8.16 × 10^−3^
Proteobacteria	Gammaproteobacteria	Enterobacterales	Enterobacteriaceae	Klebsiella_A	grimontii	2.17 × 10^−5^ [1.18 × 10^−6^–7.85 × 10^−5^]	6.51 × 10^−6^ [2.19 × 10^−7^–2.63 × 10^−5^]	−2.53 ± 0.70	8.16 × 10^−3^
Firmicutes	Bacilli	Lactobacillales	Enterococcaceae	Enterococcus_A	gilvus	2.96 × 10^−6^ [1.09 × 10^−6^–9.06 × 10^−6^]	1.53 × 10^−6^ [0.00 × 10^+0^–5.57 × 10^−6^]	−2.41 ± 0.75	1.49 × 10^−2^
Proteobacteria	Gammaproteobacteria	Enterobacterales	Enterobacteriaceae	Enterobacter	sichuanensis	1.74 × 10^−6^ [0.00 × 10^+0^–6.17 × 10^−6^]	4.04 × 10^−7^ [0.00 × 10^+0^–1.83 × 10^−6^]	−2.38 ± 0.78	2.24 × 10^−2^
Proteobacteria	Gammaproteobacteria	Enterobacterales	Enterobacteriaceae	Citrobacter	freundii	3.36 × 10^−5^ [1.12 × 10^−5^–7.62 × 10^−5^]	3.99 × 10^−5^ [1.65 × 10^−6^–1.48 × 10^−4^]	−2.32 ± 0.69	1.11 × 10^−2^
Proteobacteria	Gammaproteobacteria	Enterobacterales	Enterobacteriaceae	Klebsiella_A	michiganensis	1.28 × 10^−5^ [2.62 × 10^−6^–3.94 × 10^−5^]	2.83 × 10^−6^ [1.56 × 10^−6^–1.59 × 10^−5^]	−2.27 ± 0.68	1.31 × 10^−2^
Proteobacteria	Gammaproteobacteria	Enterobacterales	Enterobacteriaceae	Enterobacter	cloacae_M	1.55 × 10^−6^ [0.00 × 10^+0^–7.34 × 10^−6^]	6.00 × 10^−7^ [8.80 × 10^−8^–5.20 × 10^−6^]	−2.22 ± 0.80	3.84 × 10^−2^
Proteobacteria	Gammaproteobacteria	Enterobacterales	Enterobacteriaceae	Hafnia	paralvei	2.19 × 10^−5^ [7.68 × 10^−6^–9.94 × 10^−5^]	5.34 × 10^−6^ [3.11 × 10^−6^–2.76 × 10^−5^]	−2.21 ± 0.64	1.01 × 10^−2^
Proteobacteria	Gammaproteobacteria	Enterobacterales	Enterobacteriaceae	Klebsiella	pneumoniae	2.61 × 10^−5^ [5.86 × 10^−6^–1.50 × 10^−4^]	1.98 × 10^−5^ [1.86 × 10^−6^–8.75 × 10^−5^]	−2.19 ± 0.67	1.43 × 10^−2^
Fusobacteriota	Fusobacteriia	Fusobacteriales	Leptotrichiaceae	Streptobacillus	moniliformis	1.55 × 10^−5^ [5.45 × 10^−6^–2.77 × 10^−5^]	7.22 × 10^−6^ [1.77 × 10^−6^–1.69 × 10^−5^]	−2.18 ± 0.60	7.95 × 10^−3^
Proteobacteria	Gammaproteobacteria	Enterobacterales	Enterobacteriaceae	Citrobacter	unknown	1.85 × 10^−5^ [6.14 × 10^−6^–1.06 × 10^−4^]	1.12 × 10^−5^ [4.53 × 10^−6^–8.12 × 10^−5^]	−2.16 ± 0.72	2.59 × 10^−2^
Proteobacteria	Gammaproteobacteria	Enterobacterales	Enterobacteriaceae	Citrobacter	werkmanii	1.35 × 10^−5^ [4.14 × 10^−6^–6.91 × 10^−5^]	6.90 × 10^−6^ [6.13 × 10^−7^–2.37 × 10^−5^]	−2.15 ± 0.64	1.24 × 10^−2^
Proteobacteria	Gammaproteobacteria	Enterobacterales	Enterobacteriaceae	Escherichia	coli	3.86 × 10^−2^ [5.42 × 10^−3^–1.90 × 10^−1^]	1.84 × 10^−2^ [6.54 × 10^−3^–8.06 × 10^−2^]	−2.12 ± 0.60	8.37 × 10^−3^
Firmicutes	Bacilli	Lactobacillales	Enterococcaceae	Enterococcus_A	unknown	2.01 × 10^−5^ [2.22 × 10^−6^–1.29 × 10^−4^]	5.27 × 10^−6^ [5.40 × 10^−7^–4.47 × 10^−5^]	−2.12 ± 0.63	1.11 × 10^−2^
Proteobacteria	Gammaproteobacteria	Enterobacterales	unknown	unknown	unknown	3.76 × 10^−5^ [7.41 × 10^−6^–9.78 × 10^−5^]	1.69 × 10^−5^ [3.98 × 10^−6^–7.56 × 10^−5^]	−2.10 ± 0.63	1.20 × 10^−2^
Firmicutes	Bacilli	Lactobacillales	Enterococcaceae	Enterococcus_D	unknown	1.09 × 10^−5^ [2.82 × 10^−6^–3.41 × 10^−4^]	4.86 × 10^−6^ [8.65 × 10^−7^–7.83 × 10^−5^]	−2.08 ± 0.74	3.72 × 10^−2^
Proteobacteria	Gammaproteobacteria	Enterobacterales	Enterobacteriaceae	Salmonella	enterica	1.68 × 10^−4^ [3.19 × 10^−5^–2.08 × 10^−4^]	7.76 × 10^−5^ [3.43 × 10^−5^–1.43 × 10^−4^]	−2.05 ± 0.59	8.89 × 10^−3^

FC—fold change; SE—standard error. ^1^ *p* values were adjusted with false discovery rate for multiple comparisons.

**Table 4:** Species with differential abundances between baseline and week 10 in PBO (*n* = 23, log2|fold change| ≥ 2 and FDR-adjusted *p* < 0.05).

Phylum	Class	Order	Family	Genus	Species	Relative Abundance (%) Median [IQR]		Week 10 vs. Baseline	
						Baseline	Week 10	Log2 FC Mean ± SE	Adjusted *p*-Value ^1^
Increased at week 10 (31 species)									
Proteobacteria	Gammaproteobacteria	Enterobacterales	Enterobacteriaceae	Yersinia	nurmii	0.00 × 10^+0^ [0.00 × 10^+0^–0.00 × 10^+0^]	0.00 × 10^+0^ [0.00 × 10^+0^–0.00 × 10^+0^]	14.81 ± 4.29	1.42 × 10^−2^
Firmicutes_A	Clostridia	Oscillospirales	Oscillospiraceae	Flavonifractor	unknown	0.00 × 10^+0^ [0.00 × 10^+0^–0.00 × 10^+0^]	0.00 × 10^+0^ [0.00 × 10^+0^–7.13 × 10^−5^]	8.66 ± 2.04	1.39 × 10^−3^
Proteobacteria	Gammaproteobacteria	Enterobacterales	Enterobacteriaceae	Lelliottia	amnigena_A	0.00 × 10^+0^ [0.00 × 10^+0^–0.00 × 10^+0^]	3.35 × 10^−7^ [0.00 × 10^+0^–2.66 × 10^−6^]	8.22 ± 2.34	1.22 × 10^−2^
Proteobacteria	Gammaproteobacteria	Enterobacterales	Enterobacteriaceae	Providencia	rettgeri_D	0.00 × 10^+0^ [0.00 × 10^+0^–0.00 × 10^+0^]	0.00 × 10^+0^ [0.00 × 10^+0^–3.67 × 10^−6^]	7.15 ± 2.11	1.64 × 10^−2^
Proteobacteria	Gammaproteobacteria	Enterobacterales	Enterobacteriaceae	Franconibacter	helveticus	0.00 × 10^+0^ [0.00 × 10^+0^–0.00 × 10^+0^]	0.00 × 10^+0^ [0.00 × 10^+0^–2.21 × 10^−6^]	6.98 ± 2.37	4.34 × 10^−2^
Proteobacteria	Gammaproteobacteria	Enterobacterales	Enterobacteriaceae	Enterobacter	cancerogenus	0.00 × 10^+0^ [0.00 × 10^+0^–0.00 × 10^+0^]	6.41 × 10^−7^ [0.00 × 10^+0^–1.79 × 10^−6^]	6.66 ± 1.76	5.25 × 10^−3^
Proteobacteria	Gammaproteobacteria	Enterobacterales	Enterobacteriaceae	Citrobacter	gillenii	0.00 × 10^+0^ [0.00 × 10^+0^–5.80 × 10^−7^]	4.37 × 10^−7^ [0.00 × 10^+0^–1.40 × 10^−6^]	5.89 ± 1.46	2.35 × 10^−3^
Proteobacteria	Gammaproteobacteria	Enterobacterales	Enterobacteriaceae	Citrobacter	europaeus	0.00 × 10^+0^ [0.00 × 10^+0^–4.77 × 10^−6^]	2.17 × 10^−6^ [4.13 × 10^−7^–4.77 × 10^−5^]	5.25 ± 0.96	1.12 × 10^−5^
Proteobacteria	Gammaproteobacteria	Enterobacterales	Enterobacteriaceae	Enterobacter	roggenkampii	0.00 × 10^+0^ [0.00 × 10^+0^–7.33 × 10^−6^]	1.24 × 10^−5^ [1.06 × 10^−6^–4.26 × 10^−5^]	5.15 ± 0.86	1.31 × 10^−6^
Proteobacteria	Gammaproteobacteria	Enterobacterales	Enterobacteriaceae	Leclercia	adecarboxylata	0.00 × 10^+0^ [0.00 × 10^+0^–2.03 × 10^−7^]	5.17 × 10^−7^ [1.09 × 10^−7^–7.09 × 10^−6^]	4.86 ± 1.17	1.89 × 10^−3^
Proteobacteria	Gammaproteobacteria	Enterobacterales	Enterobacteriaceae	Enterobacter	cloacae_M	1.15 × 10^−7^ [0.00 × 10^+0^–1.96 × 10^−6^]	4.39 × 10^−6^ [2.72 × 10^−7^–2.77 × 10^−5^]	4.48 ± 0.89	9.80 × 10^−5^
Proteobacteria	Gammaproteobacteria	Enterobacterales	Enterobacteriaceae	Kluyvera	cryocrescens	0.00 × 10^+0^ [0.00 × 10^+0^–4.02 × 10^−7^]	2.68 × 10^−7^ [0.00 × 10^+0^–2.61 × 10^−6^]	4.31 ± 1.50	4.75 × 10^−2^
Proteobacteria	Gammaproteobacteria	Enterobacterales	Enterobacteriaceae	Klebsiella	quasivariicola	0.00 × 10^+0^ [0.00 × 10^+0^–1.12 × 10^−6^]	6.35 × 10^−7^ [0.00 × 10^+0^–8.16 × 10^−6^]	3.97 ± 1.23	2.17 × 10^−2^
Proteobacteria	Gammaproteobacteria	Enterobacterales	Enterobacteriaceae	Raoultella	ornithinolytica	8.89 × 10^−7^ [6.73 × 10^−8^–1.83 × 10^−6^]	8.91 × 10^−6^ [1.33 × 10^−6^–6.04 × 10^−5^]	3.83 ± 0.83	3.79 × 10^−4^
Proteobacteria	Gammaproteobacteria	Enterobacterales	Enterobacteriaceae	Pantoea	ananatis	0.00 × 10^+0^ [0.00 × 10^+0^–9.54 × 10^−7^]	5.68 × 10^−7^ [0.00 × 10^+0^–5.83 × 10^−6^]	3.75 ± 1.15	2.15 × 10^−2^
Proteobacteria	Gammaproteobacteria	Enterobacterales	Enterobacteriaceae	Enterobacter	ludwigii	4.87 × 10^−7^ [0.00 × 10^+0^–2.60 × 10^−6^]	7.27 × 10^−6^ [0.00 × 10^+0^–2.39 × 10^−5^]	3.63 ± 0.92	3.39 × 10^−3^
Proteobacteria	Gammaproteobacteria	Enterobacterales	Enterobacteriaceae	Pluralibacter	gergoviae	2.17 × 10^−7^ [0.00 × 10^+0^–7.54 × 10^−7^]	9.75 × 10^−7^ [0.00 × 10^+0^–9.11 × 10^−6^]	3.57 ± 1.00	1.15 × 10^−2^
Proteobacteria	Gammaproteobacteria	Enterobacterales	Enterobacteriaceae	Enterobacter	cloacae	2.24 × 10^−7^ [0.00 × 10^+0^–2.76 × 10^−6^]	3.36 × 10^−6^ [3.92 × 10^−7^–1.13 × 10^−5^]	3.40 ± 0.96	1.22 × 10^−2^
Proteobacteria	Gammaproteobacteria	Enterobacterales	Enterobacteriaceae	Enterobacter	kobei	1.50 × 10^−6^ [3.01 × 10^−7^–1.19 × 10^−5^]	1.81 × 10^−5^ [3.40 × 10^−6^–6.89 × 10^−5^]	3.39 ± 0.70	1.34 × 10^−4^
Bacteroidota	Bacteroidia	Bacteroidales	Tannerellaceae	Parabacteroides	sp900155425	4.48 × 10^−7^ [0.00 × 10^+0^–2.11 × 10^−6^]	5.68 × 10^−7^ [0.00 × 10^+0^–1.02 × 10^−5^]	3.33 ± 1.15	4.50 × 10^−2^
Proteobacteria	Gammaproteobacteria	Enterobacterales	Enterobacteriaceae	Raoultella	planticola	0.00 × 10^+0^ [0.00 × 10^+0^–7.59 × 10^−7^]	1.63 × 10^−6^ [0.00 × 10^+0^–2.39 × 10^−5^]	3.27 ± 1.07	3.22 × 10^−2^
Proteobacteria	Gammaproteobacteria	Enterobacterales	Enterobacteriaceae	Klebsiella	quasipneumoniae	5.70 × 10^−6^ [0.00 × 10^+0^–1.90 × 10^−5^]	1.71 × 10^−5^ [2.84 × 10^−6^–7.39 × 10^−5^]	3.27 ± 0.74	8.03 × 10^−4^
Proteobacteria	Gammaproteobacteria	Enterobacterales	Enterobacteriaceae	Citrobacter	unknown	9.52 × 10^−6^ [4.92 × 10^−6^–1.97 × 10^−5^]	8.82 × 10^−5^ [4.31 × 10^−6^–1.05 × 10^−3^]	3.20 ± 0.78	2.13 × 10^−3^
Proteobacteria	Gammaproteobacteria	Enterobacterales	Enterobacteriaceae	Klebsiella_A	oxytoca	4.34 × 10^−7^ [0.00 × 10^+0^–2.05 × 10^−5^]	8.55 × 10^−6^ [5.06 × 10^−7^–3.83 × 10^−5^]	3.06 ± 0.93	2.09 × 10^−2^
Proteobacteria	Gammaproteobacteria	Enterobacterales	Enterobacteriaceae	Enterobacter	unknown	2.44 × 10^−6^ [8.35 × 10^−7^–6.91 × 10^−6^]	1.66 × 10^−5^ [5.58 × 10^−6^–1.29 × 10^−4^]	2.95 ± 0.67	8.03 × 10^−4^
Proteobacteria	Gammaproteobacteria	Enterobacterales	Enterobacteriaceae	Cronobacter	malonaticus	7.73 × 10^−7^ [0.00 × 10^+0^–3.08 × 10^−6^]	3.97 × 10^−6^ [1.10 × 10^−6^–1.58 × 10^−5^]	2.86 ± 0.88	2.17 × 10^−2^
Firmicutes_C	Negativicutes	Selenomonadales	Selenomonadaceae	Megamonas	funiformis	7.02 × 10^−7^ [0.00 × 10^+0^–3.22 × 10^−3^]	1.72 × 10^−4^ [4.76 × 10^−7^–6.68 × 10^−3^]	2.82 ± 0.92	3.25 × 10^−2^
Proteobacteria	Gammaproteobacteria	Enterobacterales	Enterobacteriaceae	Klebsiella	variicola	3.05 × 10^−6^ [1.07 × 10^−6^–1.66 × 10^−5^]	2.16 × 10^−5^ [8.05 × 10^−6^–5.52 × 10^−5^]	2.73 ± 0.71	4.32 × 10^−3^
Proteobacteria	Gammaproteobacteria	Enterobacterales	Enterobacteriaceae	Citrobacter	freundii	2.50 × 10^−5^ [3.89 × 10^−6^–5.65 × 10^−5^]	5.88 × 10^−5^ [6.85 × 10^−6^–5.12 × 10^−4^]	2.17 ± 0.75	4.50 × 10^−2^
Proteobacteria	Gammaproteobacteria	Enterobacterales	Enterobacteriaceae	Citrobacter	portucalensis	7.48 × 10^−5^ [3.96 × 10^−5^–1.28 × 10^−4^]	1.15 × 10^−4^ [1.58 × 10^−5^–1.35 × 10^−3^]	2.15 ± 0.73	4.34 × 10^−2^
Proteobacteria	Gammaproteobacteria	Enterobacterales	Enterobacteriaceae	Enterobacter	sesami	1.57 × 10^−6^ [0.00 × 10^+0^–4.34 × 10^−6^]	1.01 × 10^−5^ [1.77 × 10^−6^–1.49 × 10^−5^]	2.08 ± 0.71	4.34 × 10^−2^
Decreased at week 10 (17 species)									
Firmicutes	Bacilli	Lactobacillales	Lactobacillaceae	Lactiplantibacillus	unknown	1.91 × 10^−7^ [0.00 × 10^+0^–2.24 × 10^−6^]	0.00 × 10^+0^ [0.00 × 10^+0^–0.00 × 10^+0^]	−8.61 ± 2.11	2.13 × 10^−3^
Firmicutes	Bacilli	Lactobacillales	Lactobacillaceae	Loigolactobacillus	coryniformis	2.24 × 10^−7^ [0.00 × 10^+0^–1.88 × 10^−6^]	0.00 × 10^+0^ [0.00 × 10^+0^–0.00 × 10^+0^]	−7.92 ± 2.26	1.22 × 10^−2^
Actinobacteriota	Actinomycetia	Actinomycetales	Bifidobacteriaceae	Bifidobacterium	animalis	0.00 × 10^+0^ [0.00 × 10^+0^–6.83 × 10^−7^]	0.00 × 10^+0^ [0.00 × 10^+0^–0.00 × 10^+0^]	−7.52 ± 2.61	4.70 × 10^−2^
Firmicutes	Bacilli	Lactobacillales	Lactobacillaceae	Leuconostoc	lactis	1.11 × 10^−6^ [0.00 × 10^+0^–1.51 × 10^−5^]	0.00 × 10^+0^ [0.00 × 10^+0^–1.71 × 10^−6^]	−4.89 ± 1.27	4.32 × 10^−3^
Firmicutes	Bacilli	Lactobacillales	Lactobacillaceae	Lactiplantibacillus	plantarum	4.28 × 10^−6^ [1.19 × 10^−6^–1.77 × 10^−5^]	3.25 × 10^−7^ [0.00 × 10^+0^–1.95 × 10^−6^]	−4.75 ± 0.96	1.14 × 10^−4^
Firmicutes	Bacilli	Lactobacillales	Brochotrichaceae	Barochoric	thermosphacta	4.04 × 10^−7^ [0.00 × 10^+0^–2.41 × 10^−6^]	0.00 × 10^+0^ [0.00 × 10^+0^–2.46 × 10^−7^]	−4.72 ± 1.59	4.34 × 10^−2^
Firmicutes	Bacilli	Lactobacillales	Carnobacteriaceae	unknown	unknown	0.00 × 10^+0^ [0.00 × 10^+0^–9.51 × 10^−6^]	0.00 × 10^+0^ [0.00 × 10^+0^–1.19 × 10^−6^]	−4.63 ± 1.39	2.00 × 10^−2^
Firmicutes	Bacilli	Lactobacillales	Lactobacillaceae	Weissella	cibaria	7.63 × 10^−7^ [0.00 × 10^+0^–1.44 × 10^−5^]	0.00 × 10^+0^ [0.00 × 10^+0^–1.83 × 10^−6^]	−4.50 ± 1.40	2.17 × 10^−2^
Firmicutes	Bacilli	Lactobacillales	Lactobacillaceae	Lactobacillus	unknown	4.87 × 10^−7^ [0.00 × 10^+0^–4.00 × 10^−6^]	0.00 × 10^+0^ [0.00 × 10^+0^–7.12 × 10^−7^]	−4.37 ± 1.40	2.82 × 10^−2^
Firmicutes	Bacilli	Lactobacillales	Lactobacillaceae	Latilactobacillus	sakei	7.38 × 10^−7^ [9.78 × 10^−8^–4.16 × 10^−5^]	1.82 × 10^−7^ [0.00 × 10^+0^–1.89 × 10^−6^]	−4.36 ± 1.28	1.64 × 10^−2^
Bacteroidota	Bacteroidia	Bacteroidales	Bacteroidaceae	Bacteroides	pyogenes_A	2.58 × 10^−7^ [0.00 × 10^+0^–1.91 × 10^−6^]	0.00 × 10^+0^ [0.00 × 10^+0^–2.91 × 10^−6^]	−4.13 ± 1.32	2.77 × 10^−2^
Firmicutes	Bacilli	Lactobacillales	Lactobacillaceae	Levilactobacillus	brevis	9.84 × 10^−7^ [0.00 × 10^+0^–2.92 × 10^−6^]	0.00 × 10^+0^ [0.00 × 10^+0^–3.41 × 10^−7^]	−3.96 ± 1.22	2.17 × 10^−2^
Firmicutes	Bacilli	Lactobacillales	Lactobacillaceae	Leuconostoc	unknown	1.55 × 10^−6^ [9.54 × 10^−8^–9.30 × 10^−6^]	0.00 × 10^+0^ [0.00 × 10^+0^–4.06 × 10^−6^]	−3.59 ± 1.14	2.57 × 10^−2^
Firmicutes	Bacilli	Lactobacillales	Lactobacillaceae	Lacticaseibacillus	unknown	8.65 × 10^−7^ [0.00 × 10^+0^–4.64 × 10^−5^]	2.18 × 10^−7^ [0.00 × 10^+0^–5.34 × 10^−6^]	−3.32 ± 1.13	4.34 × 10^−2^
Firmicutes	Bacilli	Lactobacillales	Lactobacillaceae	Lacticaseibacillus	paracasei	7.36 × 10^−6^ [1.58 × 10^−6^–4.00 × 10^−5^]	1.09 × 10^−6^ [9.21 × 10^−8^–2.47 × 10^−5^]	−2.81 ± 0.86	2.09 × 10^−2^
Bacteroidota	Bacteroidia	Bacteroidales	Bacteroidaceae	Bacteroides	xylanisolvens	6.70 × 10^−6^ [1.61 × 10^−6^–4.65 × 10^−4^]	5.40 × 10^−6^ [1.11 × 10^−6^–5.06 × 10^−5^]	−2.56 ± 0.79	2.17 × 10^−2^
Firmicutes	Bacilli	Lactobacillales	Lactobacillaceae	Leuconostoc	gelidum	5.29 × 10^−6^ [7.32 × 10^−7^–1.75 × 10^−5^]	1.37 × 10^−6^ [0.00 × 10^+0^–9.28 × 10^−6^]	−2.39 ± 0.82	4.36 × 10^−2^

FC—fold change; SE—standard error. ^1^ *p* values were adjusted with false discovery rate for multiple comparisons.

## Data Availability

Whole genome metagenome sequence reads used for the microbiome analysis have been deposited at NCBI SRA under the NCBI BioProject accession PRJNA994039. Health survey data are available as a Supplementary File (File S1).

## Supplementary Materials

The following supporting information can be downloaded at https://www.mdpi.com/article/10.3390/ani14030453/s1, Figure S1: Qualification screening questionnaire, Figure S2: Characteristics of supplements. (A) Placebo (Maltodextrin); and (B) probiotic and nutrient blend (PNB), Figure S3: Pruritus and health survey questions, Figure S4: Taxa post-filtering—682 species, Figure S5: Individual digital PVAS10 scores at baseline and week 10, Figure S6: Individual OA-SASI scores at baseline and week 10, Figure S7: Boxplot OA-SASI score for all weeks (subgroup = symptom onset ages 1–3), Figure S8: Boxplot OA-SASI score for all weeks (subgroup = nonseasonal symptoms), Figure S9: Boxplot digital PVAS10 score for all weeks (subgroup = nonseasonal symptoms), Figure S10: Boxplot of quality of life (QOL) for all weeks, Figure S11: Eigenvalues of the first 25 PCoA axes, Figure S12: PCoA1 shift from baseline to week 10, Figure S13: Phyla relative abundances, Figure S14: Abundances all six supplemented probiotics from baseline to week 10, Figure S15: KEGG Orthology (KO) eigenvalues of the first 25 PCoA axes, Figure S16: Principal coordinate analysis (PCoA) of the Kyoto Encyclopedia of Genes and Genomes Orthology (KO) terms, Figure S17: Baseline PCoA1 vs. change in PCoA1 scores from baseline to week 10, Figure S18: α-diversity metrics at baseline vs. change in PCoA1 scores from baseline to week 10, Figure S19: Volcano plots demonstrating the fold-change (FC) in the differential abundance analysis of gut bacteria at the species level between subjects in the first and the third tertile in PNB group at baseline (tertiles defined by the magnitude of shift along the PCoA1 axis), Figure S20: Volcano plots demonstrating the fold-change (FC) in the differential abundance analysis of gut bacteria at the species level at week 10 compared to week 0 in low responders in PBO group (*n* = 8), Figure S21: Volcano plots demonstrating the fold-change (FC) in the differential abundance analysis of gut bacteria at the species level at week 10 compared to week 0 in high responders in PBO group (*n* = 8), Figure S22: Volcano plots demonstrating the fold-change (FC) in the differential abundance analysis of gut bacteria at the species level at week 10 compared to week 0 in high responders in PNB group (*n* = 9), Figure S23: Volcano plots demonstrating the fold-change (FC) in the differential abundance analysis of gut bacteria at the species level at week 10 compared to week 0 in low responders in PNB group (*n* = 9), Figure S24: Volcano plots demonstrating the fold-change (FC) in the differential abundance analysis of gut bacteria at the species level between low responders (LRs, *n* = 9) and high responders (HRs, *n* = 9) in PNB group at baseline, Figure S25: The α-diversity metrics between HRs, MRs, and LRs at baseline in both groups, Figure S26: Volcano plots demonstrating the fold-change (FC) in the differential abundance analysis of gut bacteria at the species level between low responders (LRs, *n* = 8) and high responders (HRs, *n* = 8) in PBO group at baseline, Table S1: Eligibility criteria, Table S2: Characteristics of each participant, Table S3: Median canine pruritus severity scores (digital PVAS10) at baseline and week 10, Table S4: Percentage change in OA-SASI from baseline as ranked improvement scores, Table S5: OA-SASI scores at week 2 by lesion and body site, Table S6: Week 10 Health and behavioral outcomes, Table S7: (a) KO terms with differential abundances between baseline and week 10 in PNB (*n* = 27, log2|fold change| ≥ 2 and FDR-adjusted *p* < 0.05), (b) KEGG Enzymes with differential abundances between baseline and week 10 in PNB group (*n* = 27, log2|fold change| ≥ 2 and FDR-adjusted *p* < 0.05), Table S8: (a) KO terms with differential abundances between baseline and week 10 in PBO group (*n* = 23, log2|fold change| ≥ 2 and FDR-adjusted *p* < 0.05), (b) KEGG Enzymes with differential abundances between baseline and week 10 in PBO (*n* = 23, log2|fold change| ≥ 2 and FDR-adjusted *p* < 0.05), Table S9: Species with differential abundances between subjects in the first and the third tertile in PNB group at baseline (tertiles defined by the magnitude of shift along the PCoA1 axis, log2|fold change| ≥ 2 and FDR-adjusted *p* < 0.05), Table S10: Species with differential abundances between baseline and week 10 in low responders in PBO group (*n* = 8, log2|fold change| ≥ 2 and FDR-adjusted *p* < 0.05), Table S11: Species with differential abundances between baseline and week 10 in high responders in PBO group (*n* = 8, log2|fold change| ≥ 2 and FDR-adjusted *p* < 0.05), Table S12: Species with differential abundances between baseline and week 10 in high responders in PNB group (*n* = 9, log2|fold change| ≥ 2 and FDR-adjusted *p* < 0.05), Table S13: Species with differential abundances between baseline and week 10 in low responders in PNB group (*n* = 9, log2|fold change| ≥ 2 and FDR-adjusted *p* < 0.05), Table S14: Species with differential abundances between low responders (LRs, *n* = 9) and high responders (HRs, *n* = 9) in PNB group at baseline (log2|fold change| ≥ 2 and FDR-adjusted *p* < 0.05), Table S15: Species with differential abundances between low responders (LRs, *n* = 8) and high responders (HRs, *n* = 8) in PBO group at baseline (log2|fold change| ≥ 2 and FDR-adjusted *p* < 0.05), File S1: Raw health survey data.
