# Supplementary material for: A Randomized Controlled Trial to Evaluate the Impact of a Novel Probiotic and Nutraceutical Supplement on Pruritic Dermatitis and the Gut Microbiota in Privately Owned Dogs
Source: Animals (Basel). 2024 Jan 30;14(3):453. doi: 10.3390/ani14030453 (PMC10854619; doi:10.3390/ani14030453)
Supplement: Supplementary file 1 [file animals-14-00453-s001.zip › Supplementary Figures.pdf]

**Supplemental Figure S1:** Qualification screening questionnaire

In which state was [Pet Name] **predominantly raised**? _________

In which state does [Pet Name] **live now**? _________

How would you describe [Pet Name]'s **coat**? (Select the best description)

- Short-coated, medium coated, long-coated, wire-coated, curly-coated, hairless

**How old** is [Pet Name]?

- < 1 year old, 1-12 years old, > 12 years old

Which of the following best describes [Pet Name]'s **body condition**?

| UNDERWEIGHT 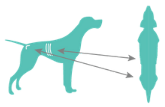 | IDEAL 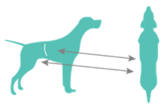 | OVERWEIGHT 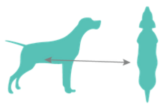 | VERY OVERWEIGHT 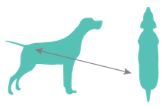 | OBESE 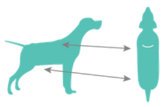 | SEVERELY OBESE 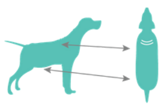 |
|---|---|---|---|---|---|
| **Underweight** Ribs & hips highly visible, appear to jut out | **Ideal** A visible waist, outline of ribs visible, belly tucks up | **Overweight** Slight waist visible from top, ribs covered by excess fat | **Very Overweight** Waist barely visible, fat deposits on the back | **Obese** No waist visible from top or side, lots of fat deposits on the back | **Severely Obese** Abdomen is bigger than chest, belly severely rounded |

What is [Pet Name]'s **current weight** (in pounds)? _________ (> 50 lb)

How would you describe [Pet Name]'s **current diet**? (Select all that apply)

- Nom Nom, Other commercial fresh cooked food, Home prepared fresh cooked, food, Commercial raw food, Home prepared raw food, Frozen food, Kibble, Canned food, Dehydrated food, Other food

Has [Pet Name} been on Nom Nom for **>1 month**? YES or NO

Are you willing to keep [Pet Name] on **only Nom Nom** throughout the study period (10 weeks)? YES or NO

Does [Pet Name] have any of the following **health conditions**? (Select all that apply)

- Diabetes, Hypothyroidism, Cushing's Disease, Pancreatitis, Kidney Disease or Failure, Liver Disease, Heart Disease, Cancer, Current pregnant or lactating, anticipated surgery or surgery within the last 3 months, Nope - they're pretty healthy (besides their itchy skin!)

Has [Pet Name] had any of the following **skin infections or skin diseases** in the last 1 month? (Select all that apply)

- Bacterial pyoderma or Malassezia dermatitis, Uncontrolled flea allergy dermatitis, Seborrhea, Sarcoptic mange, Demodicosis/Demodex canis, Dermatophytosis (ringworm), Mites - i.e. Cheyletiella or Trombicula autumnalis, Louse - i.e. Trichodectes canis, Ectoparasitic disorders i.e. scabies, None of the above - they're just itchy!

Does [Pet Name] have a known **food allergy**?

- Yes, No, Maybe

Have you tried [Pet Name] on an **elimination** diet or **hydrolyzed** diet? YES or NO

- YES → Did [Pet Name]'s itchiness improve? YES or NO

Has [Pet Name] been diagnosed with **canine atopic dermatitis (CAD)** and have **visible skin allergies**?

- Yes, No, What's that?
  - YES → Was this diagnosed by a vet? YES or NO

Which of the following apply to [Pet Name]? (select all that apply)

- Visibly itchy and irritated skin (red, bumps, scratches) - all over; Visible itchy and irritated skin - but localized; Constant licking, biting, or chewing - especially at their paws; Dry, crusty, or oily skin; Dull coat; Hair loss; None of these sound like my dog

Do you know when [Pet Name]'s itchy skin allergies started?

- When they were a puppy (<1 years old), As a young adults (1-3 years old), As an adults (3-7 years old), As a mature dog (7+ years old)

Do you know if [Pet Name]'s allergies are seasonal?

- Yes - they are, I'm not sure - but they're itchy

Does [Pet Name] also suffer from gastrointestinal issues? *Like frequent diarrhea, constipation, vomiting, excessive gas.* YES or NO

Please select which **medications** [Pet Name] is receiving for their allergies/itchiness? (select all that apply).

- steroids, antihistamines, immunosuppressants, apoquel (oclacitinib), cytopoint (lokivetmab) and allergen immunotherapy, oral allergy therapy, topicals, and medicated hypo-allergic shampoos and wipes, none of the above, other
    - OTHER → What other medications is [Pet Name] on for their itchy skin allergies?

Has [Pet Name] been on these **medications** (dose and frequency) for at least one month? YES or NO

And [Pet Name] still has **visibly itchy skin**? YES or NO

Would you be willing to keep [Pet Name]'s **medication regime (dose & frequency, or no medication) the same** for at least 1 month before the beginning of the study and throughout the study period (10 weeks)? YES or NO

Is [Pet Name] on any **flea** allergy medications? YES or NO

Can you continue to keep [Pet Name] **on**/**off** of flea allergy medications for the duration of the 10 week study? YES or NO

Has [Pet Name] been on any **antibiotics, antifungals, or antiparasitics** in the last 1 month? YES or NO

Is [Pet Name] currently on a **supplement** that contains any of the following ingredients: prebiotics, probiotics, yeast fermentate or brewer's yeast, vitamin E, B-vitamins, vitamin A or carotenoids (i.e. beta-carotene, lutein), or fatty acids (i.e. fish or marine oil)? *Or any other antioxidants, plant extracts, cultured or fermented foods (i.e. yogurt kefir, unpasteurized dairy), or yeast products.* YES or NO

---

**Study Information & Consent**

For the purpose of this study we would like to compare [Pet Name]'s itchiness, skin health, and overall health before, during, and after taking an allergy targeting supplement. *Supplements will be provided at no cost. You will be rewarded up to $200 in Nom Nom credit for active participation. The study is 10 weeks long. You can willingly remove [Pet Name] from the study at any point.*

[Pet Name] will be randomized to one of 2 groups: placebo (no active ingredients) or yeast fermentate + nutrient blend. You will not know which group [Pet Name] is in until the end of the study. *All ingredients in the supplement are approved by AAFCO and 100% safe for your dogs.*

During the study you will have to mix in the supplement into [Pet Name]'s food, provide two stool samples (at the beginning and end), and fill out 5 surveys (at the start of the study and at the end of weeks 2, 4, 7, and 10). *Don't worry we'll provide kits for microbiome collection. The surveys will require you to examine [Pet Name]'s body for hair loss, redness, and bumps, rate their itchiness, and answer additional health, wellness, and compliance questions - it shouldn't take long but we want you to be thorough. Each survey missed or stool sample not received will result in a deduction of $50 from the possible $200 Nom Nom credit.*

- I'm ready to do this
- This is to much for me to commit to

This is great news! We'll also need your **consent** to use [Pet Name]'s de-identified information - but never your personal information - for purposes including publication of studies.

- I permit my dog's participation in this study
- I do not permit my dog's participation in this study

**Responses in RED indicate disqualification from trial eligibility*

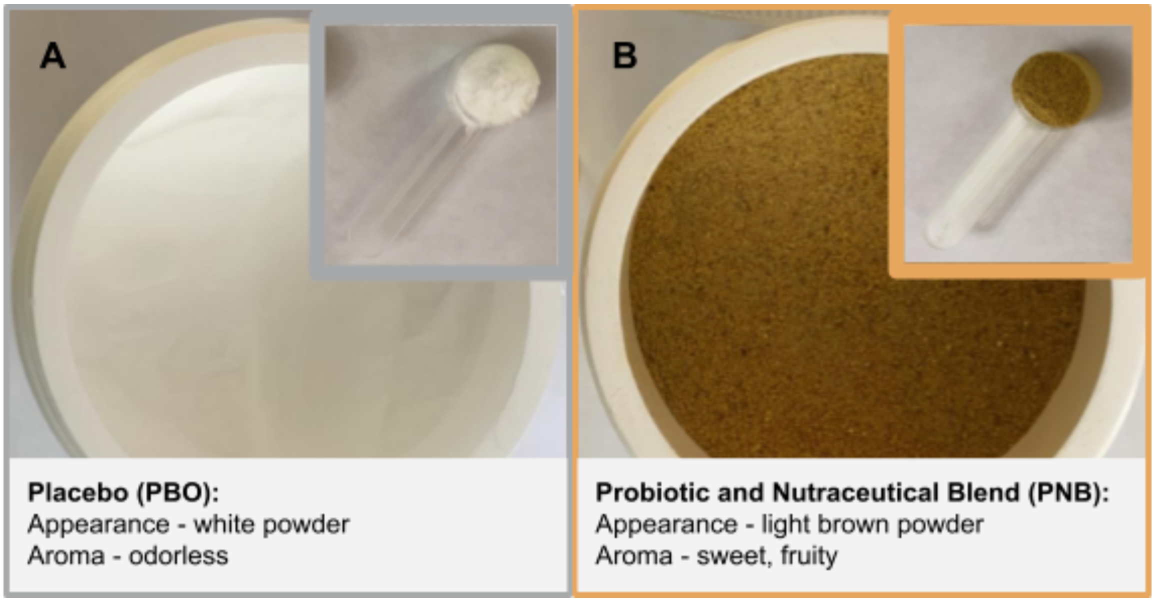


**Supplemental Figure S2**: Characteristics of supplements. **A**: Placebo (Maltodextrin); **B**: Probiotic and Nutrient Blend (PNB)

**Supplemental Figure S3:** Pruritus and health survey

**Canine Pruritus Severity Scale:** - *asked at weeks 0, 2, 4, 7, and 10*

This scale is designed to measure the severity of [Pet Name]'s itching!
Itching can include scratching, biting, licking, chewing, nibbling or rubbing.

Please read all the descriptions below carefully.

**Normal Dog**
I don't think there is a problem

**Very Mild Itching/only occasional episodes**
The dog is slightly more itchy than it was before the skin problem started

**Mild Itching/a bit more frequent**
Wouldn't itch when sleeping, eating, playing, exercising or being distracted

**Moderate Itching/regular episodes**
Itching might occur at night (if observed), but not when eating, playing, exercising or being distracted

**Severe Itching/prolonged episodes**
Itching might occur at night (if observed), and also when eating,playing,exercising or being distracted

**Extremely Severe Itching/almost continuous**
Itching doesn't stop whatever is happening, even in the consulting room (needs to be physically restrained from itching)

Based on the **descriptions above**, **move the slider to anywhere on the horizontal line** to indicate the point at which you think [Pet Name]'s level of itchiness lies.

**Weeks 1 - 10:** The slider is in the position you last left it - has [Pet Name]'s itchiness improved or worsened since then?

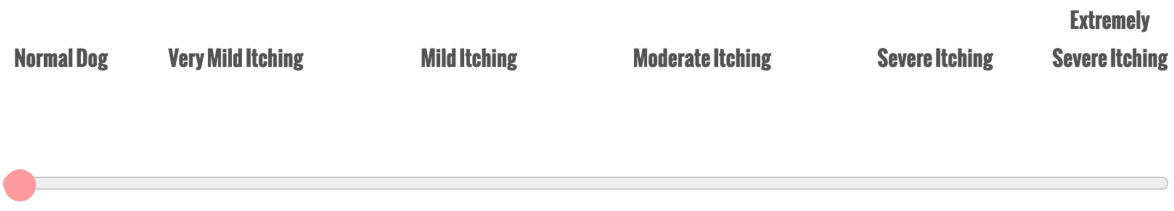

**<u>Owner Modified Canine Atopic Dermatitis Extent Severity Index:</u>** *- asked at weeks 0, 2, 4, 7, and 10*

This is used to assess skin lesions on individual body sites on your dog.

Please examine the following **regions** on [Pet Name] closely:

- FACE
- EARS
- PAWS
- LIMBS
- UNDERSIDE

...for the following types of **trauma**:

- HAIR LOSS
- SKIN REDNESS/IRRITATION
- HARD THICKENED SKIN/BUMPS

For each **region** please rank the severity of the **trauma** from 0 (none) to 3 (severe).

Looking at [Pet Name]'s **FACE** please rank the following:

- Hair Loss: *0 = None; 1 = Mild; 2 = Moderate; 3 = Severe*
- Skin Redness/irritation: *0 = None; 1 = Mild; 2 = Moderate; 3 = Severe*
- Hard thickened skin/bumps: *0 = None; 1 = Mild; 2 = Moderate; 3 = Severe*

Looking at [Pet Name]'s **EARS** please rank the following:

- Hair Loss: *0 = None; 1 = Mild; 2 = Moderate; 3 = Severe*
- Skin Redness/irritation: *0 = None; 1 = Mild; 2 = Moderate; 3 = Severe*
- Hard thickened skin/bumps: *0 = None; 1 = Mild; 2 = Moderate; 3 = Severe*

Looking at [Pet Name]'s **PAWS** please rank the following:

- Hair Loss: *0 = None; 1 = Mild; 2 = Moderate; 3 = Severe*
- Skin Redness/irritation: *0 = None; 1 = Mild; 2 = Moderate; 3 = Severe*
- Hard thickened skin/bumps: *0 = None; 1 = Mild; 2 = Moderate; 3 = Severe*

Looking at [Pet Name]'s **LIMBS** please rank the following:

- Hair Loss: *0 = None; 1 = Mild; 2 = Moderate; 3 = Severe*
- Skin Redness/irritation: *0 = None; 1 = Mild; 2 = Moderate; 3 = Severe*
- Hard thickened skin/bumps: *0 = None; 1 = Mild; 2 = Moderate; 3 = Severe*

Looking at [Pet Name]'s **UNDERSIDE** please rank the following:

- Hair Loss: *0 = None; 1 = Mild; 2 = Moderate; 3 = Severe*
- Skin Redness/irritation: *0 = None; 1 = Mild; 2 = Moderate; 3 = Severe*
- Hard thickened skin/bumps: *0 = None; 1 = Mild; 2 = Moderate; 3 = Severe*

**<u>Health Questions:</u>** - *asked at weeks 0, 2, 4, 7, and 10*

**When answering these questions please think about [Pet Name]'s health.**

***At this point….***

How would you rate [Pet Name]'s overall health from **0 (very poor) to 10 (excellent)**?

0 1 2 3 4 **5** 6 7 8 9 10

How would you describe [Pet Name]'s **<u>overall stress</u>** level?

- Not stressed, Mildly stressed, Average stress level, Somewhat stressed, Very stressed

How would you rate [Pet Name]'s **<u>overall energy</u>**?

- Very Low, Low, About average, High, Very High

How would you rate [Pet Name]'s **<u>quality of life</u>** on a scale from **0 (very poor) to 10 (very high)?**

*Quality of life refers to [Pet Name]'s ability to meet their basic needs (eat, drink, move, relieve themselves) all without pain or discomfort, and further exhibits signs of enjoyment and happiness in their day-to-day life.*

0 1 2 3 4 **5** 6 7 8 9 10

**<u>Allergy Description</u>**: - *asked at week 0*

Which of the following best describes [Pet Name]'s allergies? (Select all that apply)

- Associated with Diarrhea, Associated with Food, Mostly involves back, Mostly involves belly and armpits, Mostly involves face, Mostly involves ears, Mostly involves feet, Occurs all over body, Seasonal Symptoms, None of the above
  - None of the above → please describe ________

**<u>Skin & Coat questions</u>**: - *asked at weeks 0, 2, 4, 7, and 10*

***At this point….***

How would you describe [Pet Name]'s **skin type**?

- Dry/flaky skin, Frequent irritations with red bumps and/or hot spots, Normal, Oily, discolored and/or smelly, None of the above
    - None of the above → please describe ________

Which of the following describes [Pet Name]'s **coat** (choose all that apply)?

- Dull, Shiny, Dry, Oily, Soft, Coarse, Thin, Thick, Bald spots, Extensive hair loss, Appears healthy, Appears unhealthy, None of the above
    - None of the above → please describe ________

**When answering these questions please think about [Pet Name]'s skin & coat.**

***At this point...***

How **disruptive** would you rate [Pet Name]'s skin condition to your household on a scale from **0 (not disruptive at all) to 10 (very disruptive)?**

0 1 2 3 4 **5** 6 7 8 9 10

The amount of **scratching/itching** of their skin that [Pet Name] is currently displaying on a scale from **0 (not scratching at all) to 10 (scratching all of the time)?**

0 1 2 3 4 **5** 6 7 8 9 10

The amount of **licking** of their skin that [Pet Name] is currently displaying on a scale from **0 (not licking at all) to 10 (licking all over)?**

0 1 2 3 4 **5** 6 7 8 9 10

The **redness** of [Pet Name]'s skin (in areas displaying red) on a scale from **0 (not red at all) to 10 (extremely red)?**

0 1 2 3 4 **5** 6 7 8 9 10

The **overall condition** of [Pet Name]'s skin and coat on a scale from **0 (very healthy) to 10 (extremely poor)?**

0 1 2 3 4 **5** 6 7 8 9 10

How much **hair loss** is [Pet Name] currently experiencing on a scale from **0 (no hair loss) to 10 (extreme hair loss)?**

0 1 2 3 4 **5** 6 7 8 9 10

**Additional Information Questions:** - *asked at weeks 0, 2, 4, 7, and 10*

***At this point….***

Which of the following best represents [Pet Name]'s **stool** on average?

| 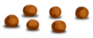 | 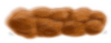 | 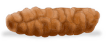 | 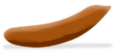 | 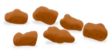 | 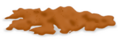 | 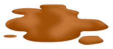 |
|---|---|---|---|---|---|---|
| Separate hard lumps (like nuts) | Sausage-shaped (but lumpy) | Sausage-shaped (but with cracks on surface) | Sausage or snake-like (smooth and soft) | Soft blobs with clear-cut edges | [Pet Name] pieces with ragged edges (mushy) | Watery/no solid pieces (entirely liquid) |

How much time does [Pet Name] **spend outside per day**?

- None, 0-1 hours/day, 1-4 hours/day, 4-8 hours/day, >8 hours/day

**Recency of Bath**

Week 0 - How recently have you bathed [Pet Name]?

- Within the last week, within the last month, within the last three months, not recently - I only bathe my dog occasionally
  - Within the last week, within the last month → Did you use any medicated or hypo-allergenic shampoos? YES or NO
    - YES→ Which shampoo did you use? _________

Weeks 2, 4, 7, and 10 - Have you bathed [Pet Name] in the time since the last survey you took? YES or NO

- YES→ Did you use any medicated or hypo-allergenic shampoos?
- YES→ Which shampoo did you use? _________

**Compliance questions:** - *asked at weeks 2, 4, 7, and 10*

At this point, how would you rate [Pet Name]'s **acceptance** of the treatment?

1 poor 2 fair 3 average 4 very good 5 excellent

Since we last checked, did [Pet Name] skip taking the treatment altogether on one or more days?

- TRUE → How many days did your dog skip taking the treatment?

Does [Pet Name] always eat the **entire dose** of their treatment?

- FALSE → How often is this the case and how much does [Pet Name] leave behind? - *Please describe*.

Did you **change** any of [Pet Name]'s medications?

- TRUE → What changes did you make to your dog's medication? *Please describe*.

Did you significantly change [Pet Name]'s **diet**?

- TRUE → What changes did you make to their diet? *Please describe*.

Did you significantly change [Pet Name]'s exercise habits?

- TRUE → What changes did you make to your dog's exercise habits? *Please describe*.

Any additional notes on health improvement or adverse effects that you would like to report?

**End of Study questions:** *- asked at week 10*

Please **rate** how easy or hard performing each of the following study parameters was:
*1 = Very Easy; 2 = Easy; 3 = Neutral; 4 = Difficult; 5 = Very Difficult*

- Gathering & sending 2 stool samples
- Completing five surveys
- Administering the study supplement daily
- Adhering to guidelines for 10 week study period

Please **rate** how important or impactful you think the results of this study will be, with 5 stars being very impactful! (5-star scale)

Anything else you'd like to share with us about your research participation or experience with the R&D team?

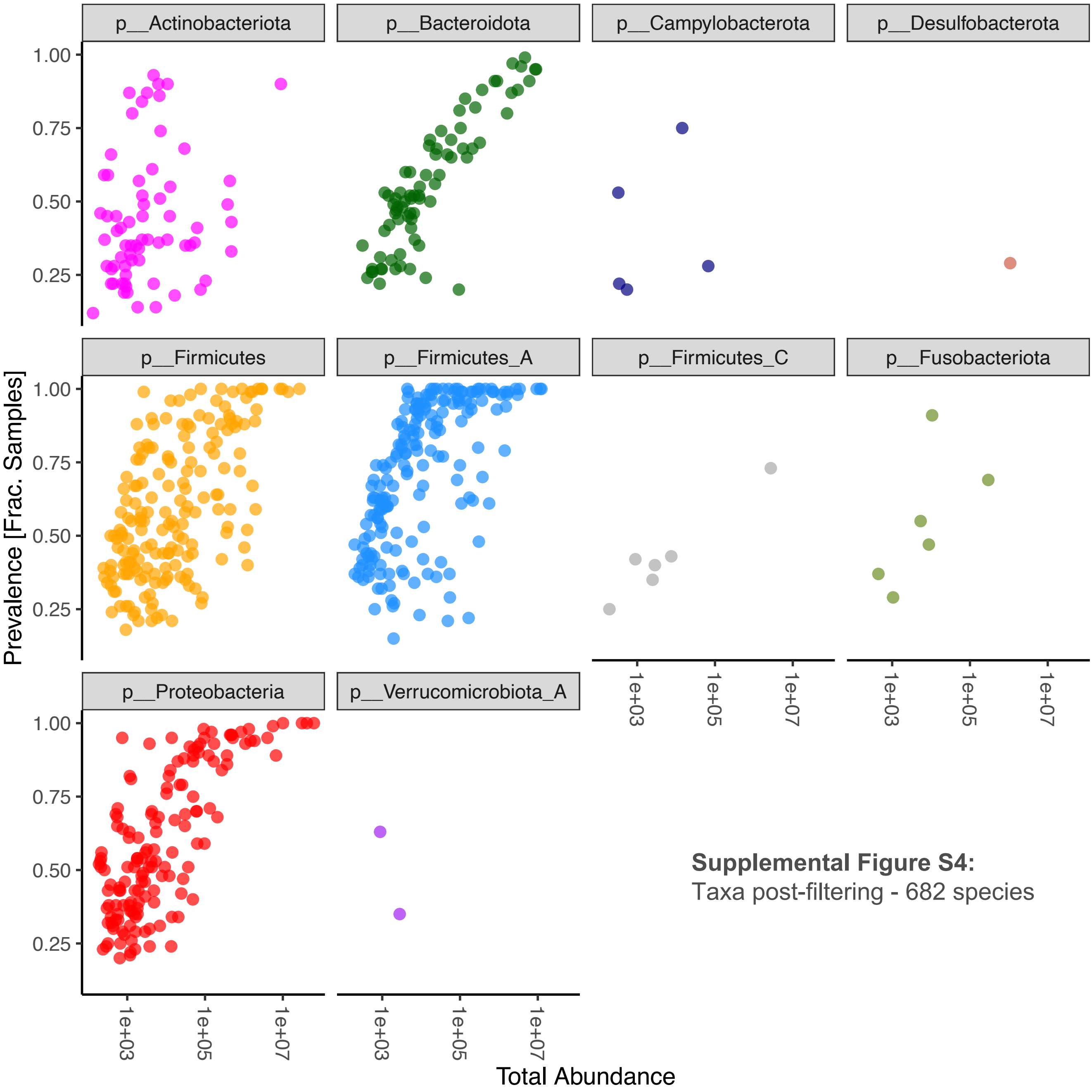


**Supplemental Figure S4:**
Taxa post-filtering - 682 species

**Supplemental Figure S5:** Individual digital PVAS10 scores baseline and week 10

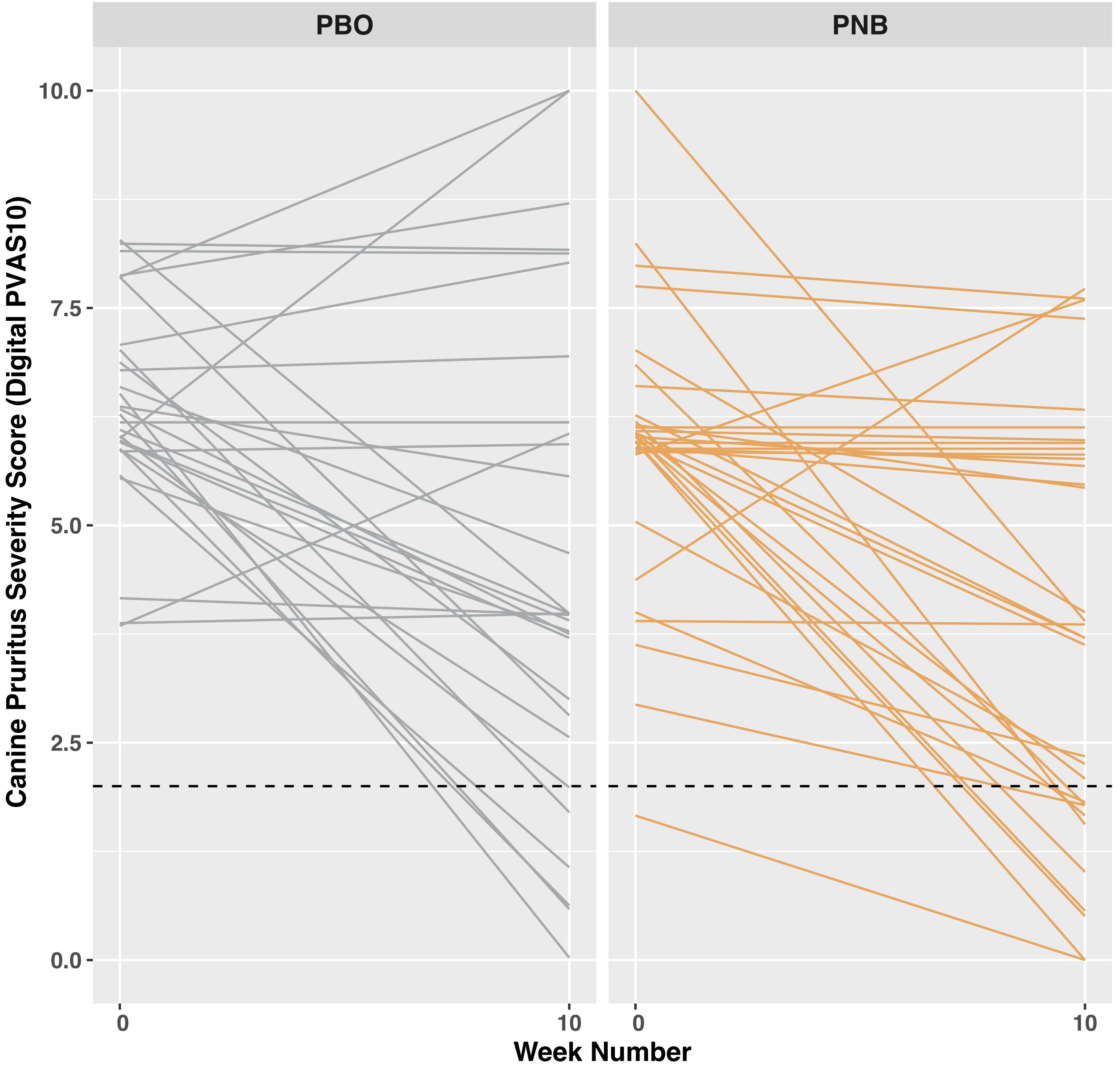

**Supplemental Figure S6:** Individual OA-SASI scores baseline and week 10

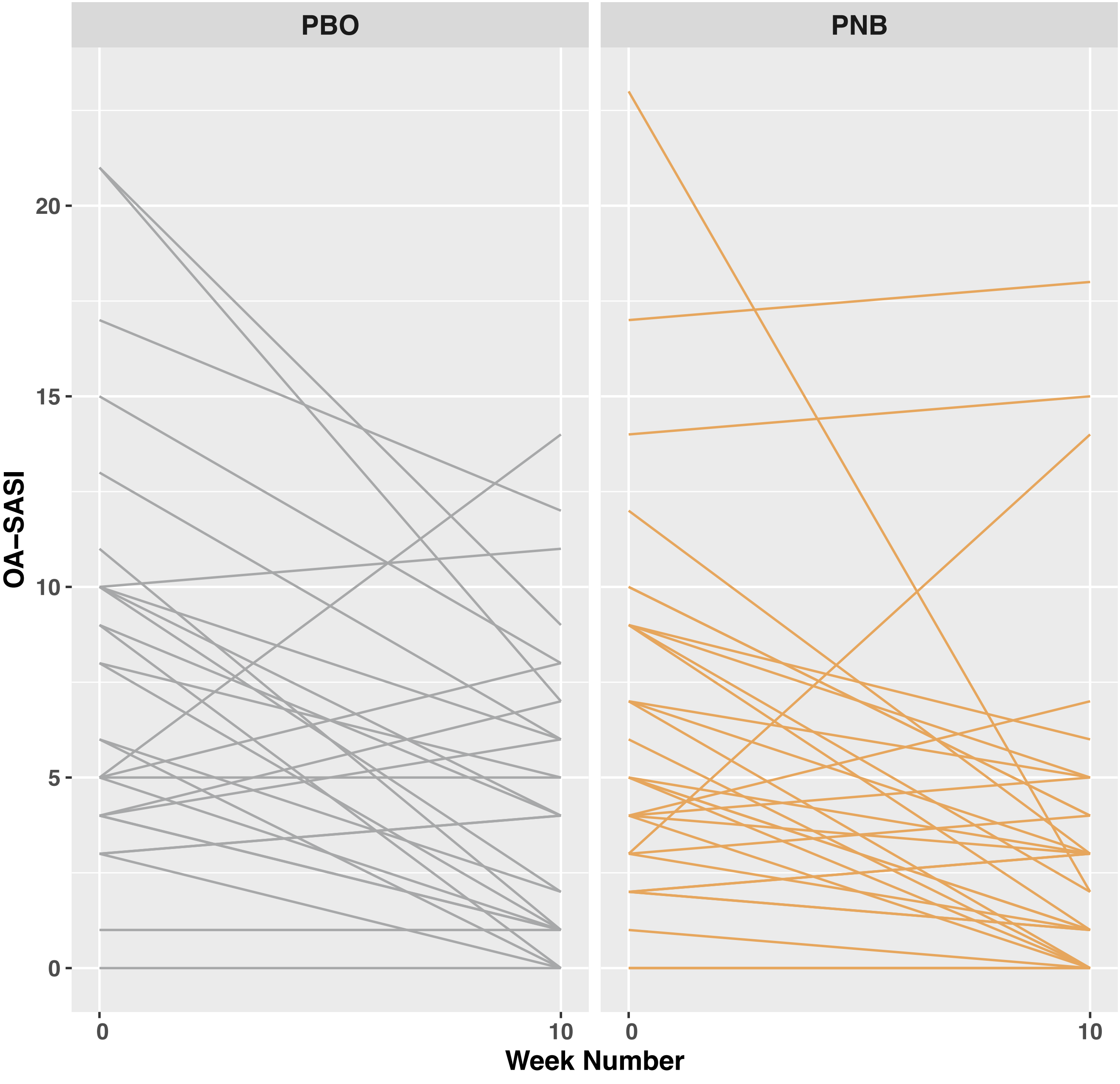

**Supplemental Figure S7:** Boxplot OA-SASI score for all weeks (subgroup = symptom onset ages 1-3)

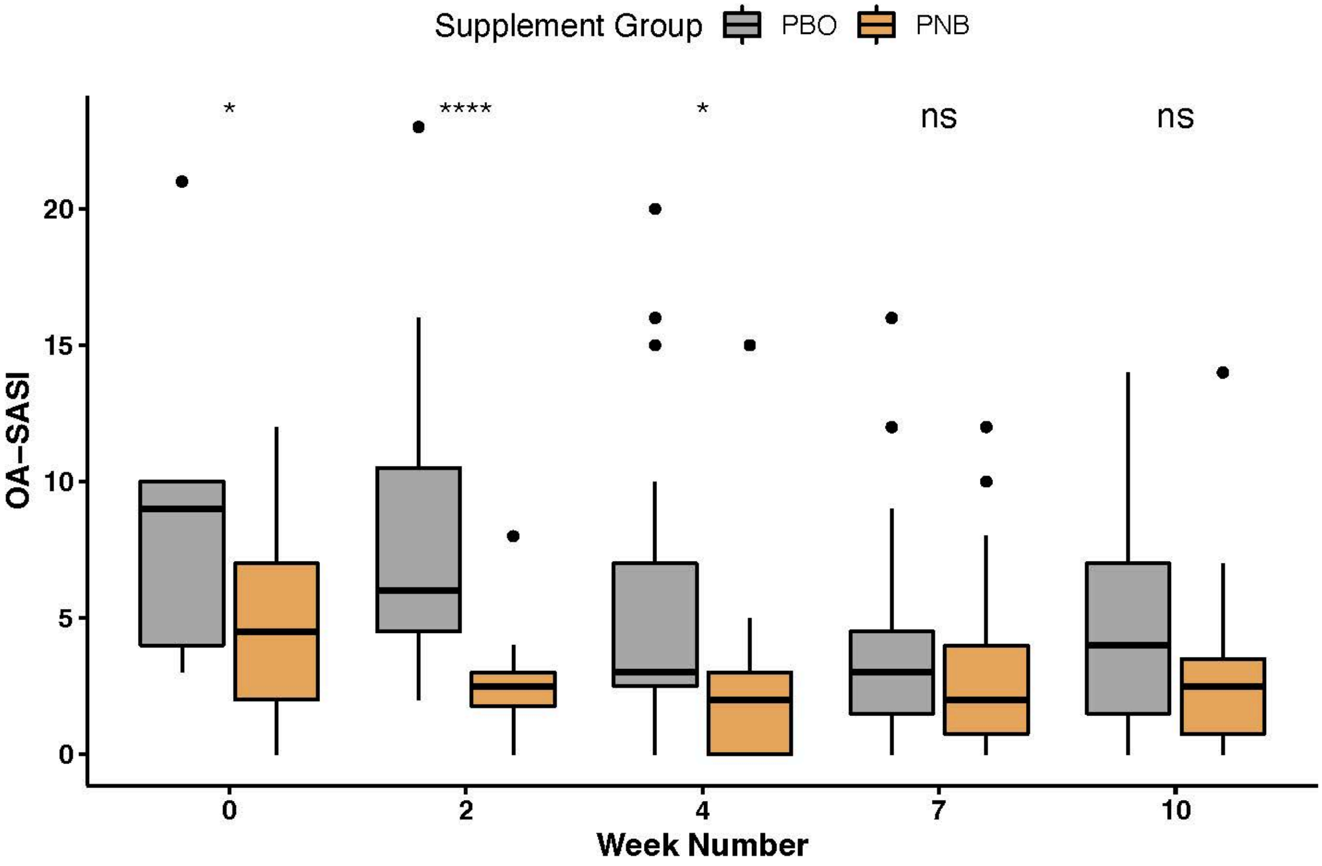

**Supplemental Figure S8:** Boxplot OA-SASI score for all weeks (subgroup = nonseasonal symptoms)

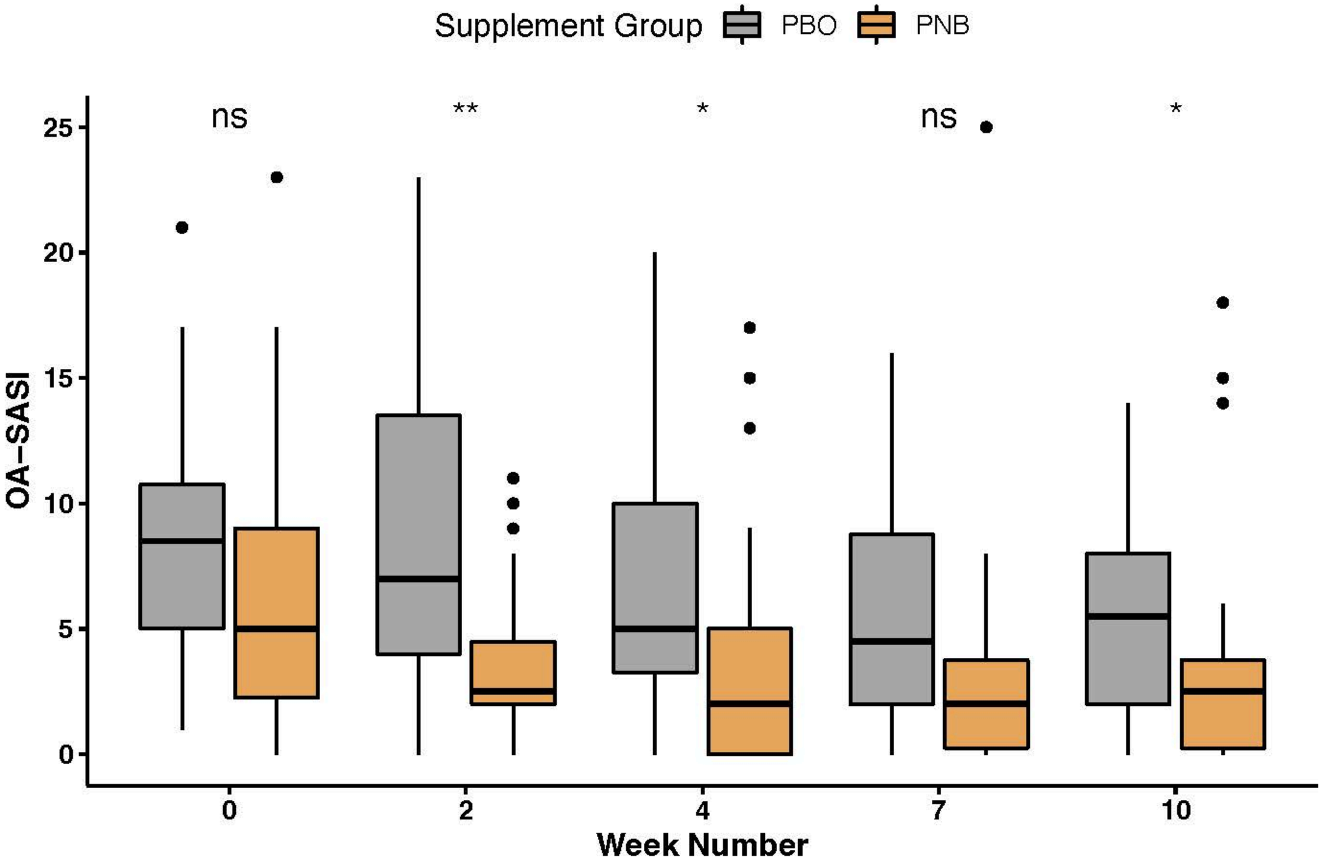

**Supplemental Figure S9**: Boxplot digital PVAS10 score for all weeks (subgroup = nonseasonal symptoms)

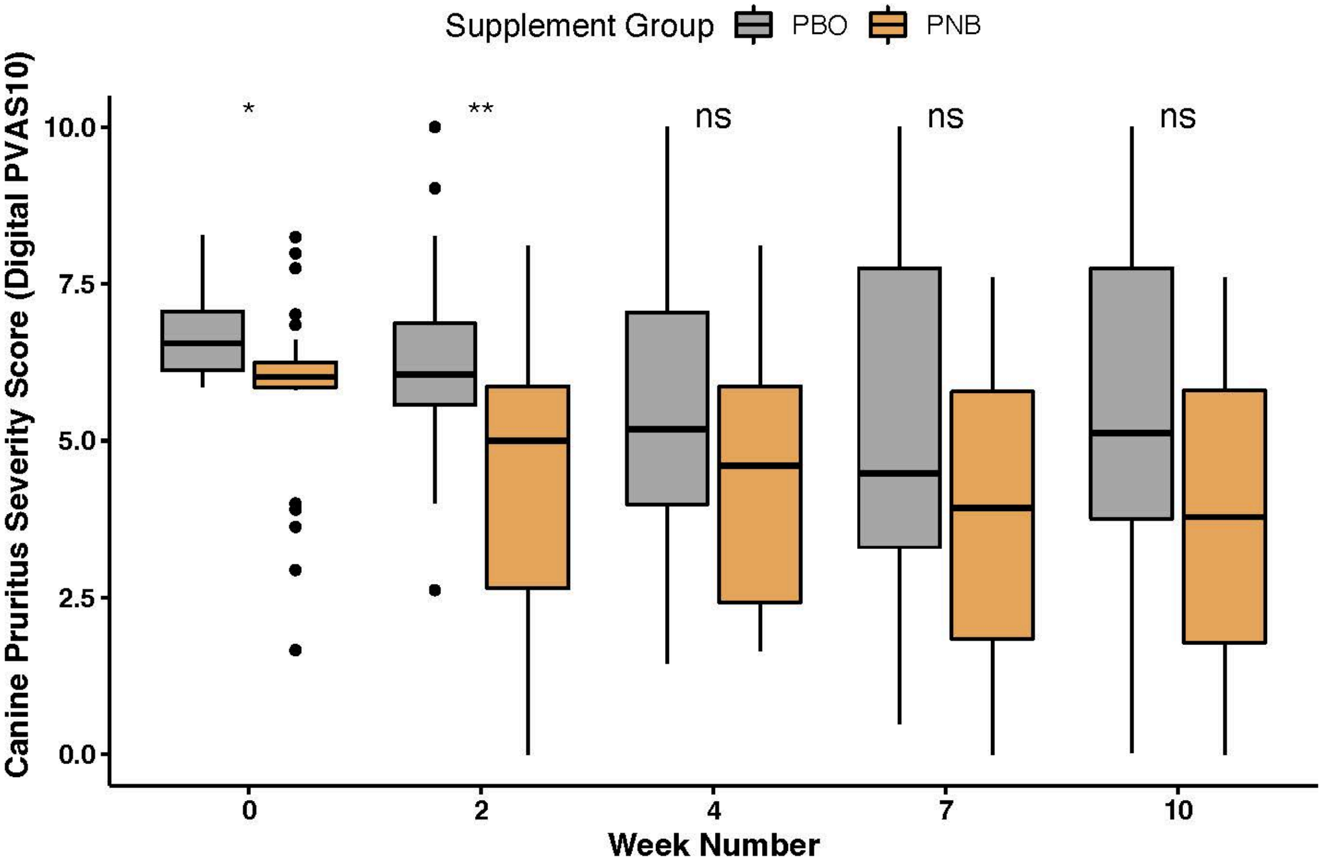

**Supplemental Figure S10:** Boxplot of quality of life (QOL) for all weeks

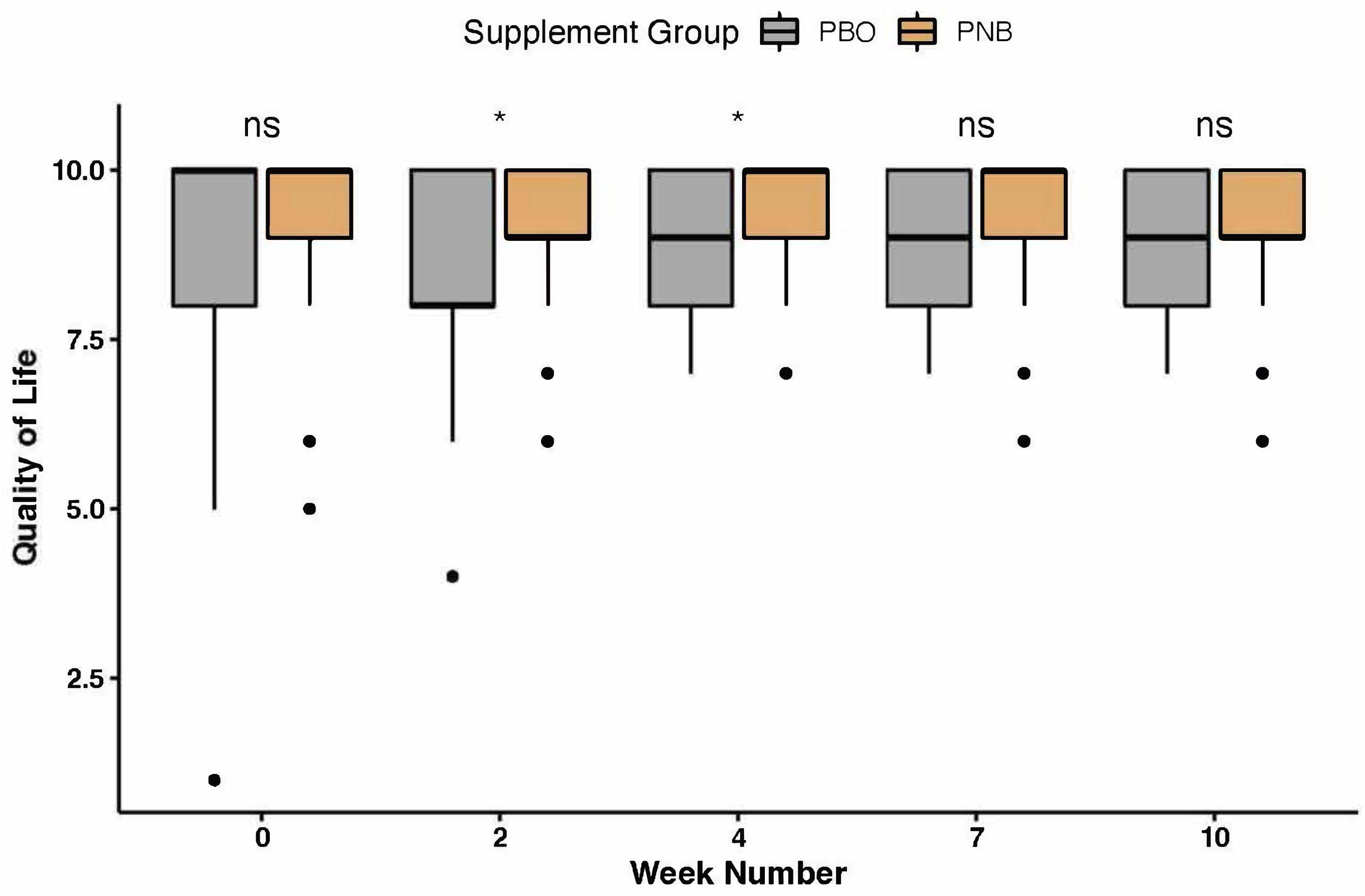

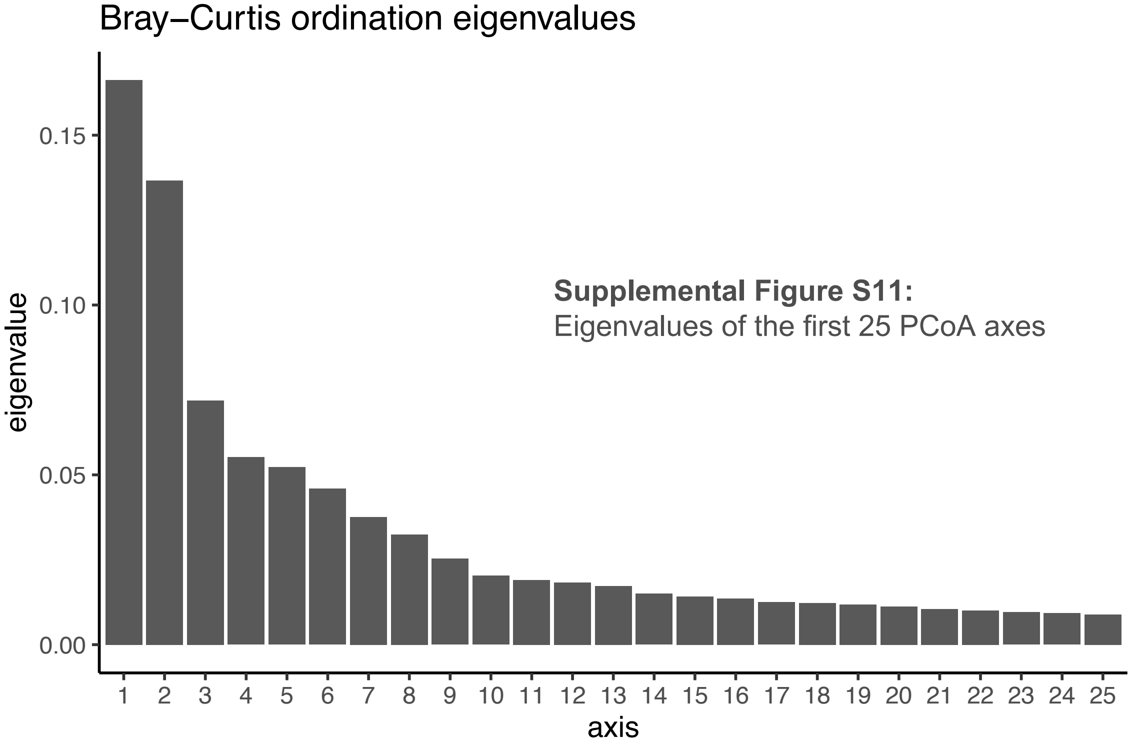


**Supplemental Figure S11:**
Eigenvalues of the first 25 PCoA axes

**Supplemental Figure S12:** PCoA1 shift from baseline to week 10

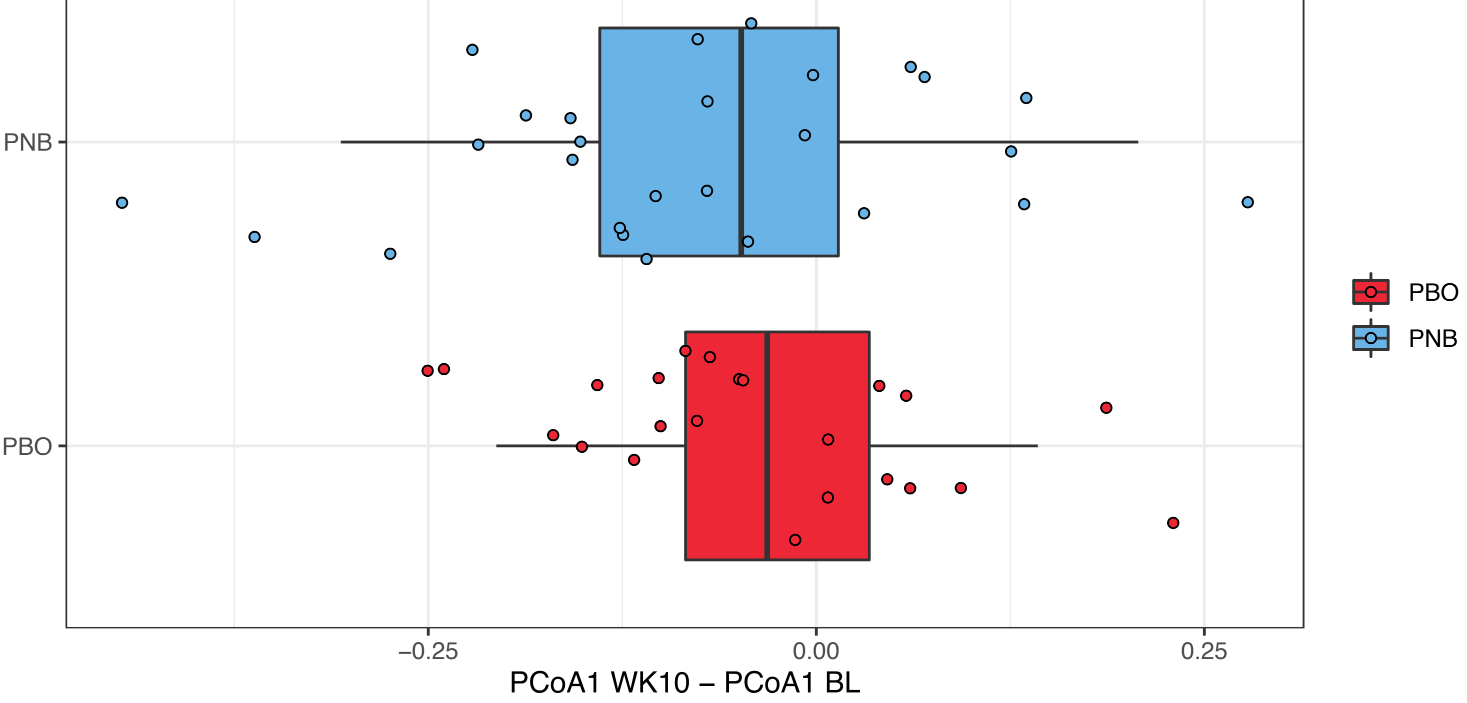

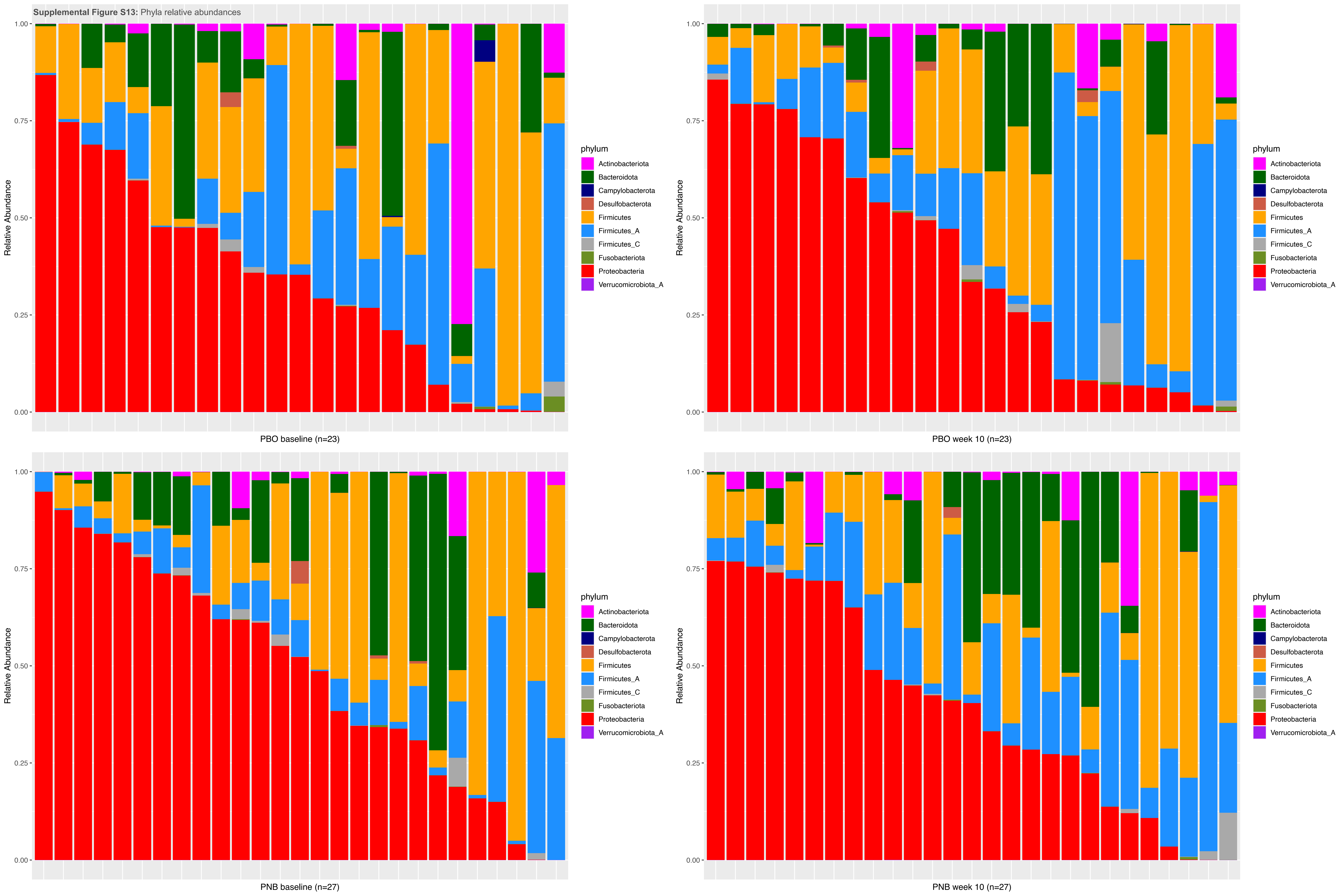

**Supplemental Figure S13:** Phyla relative abundances

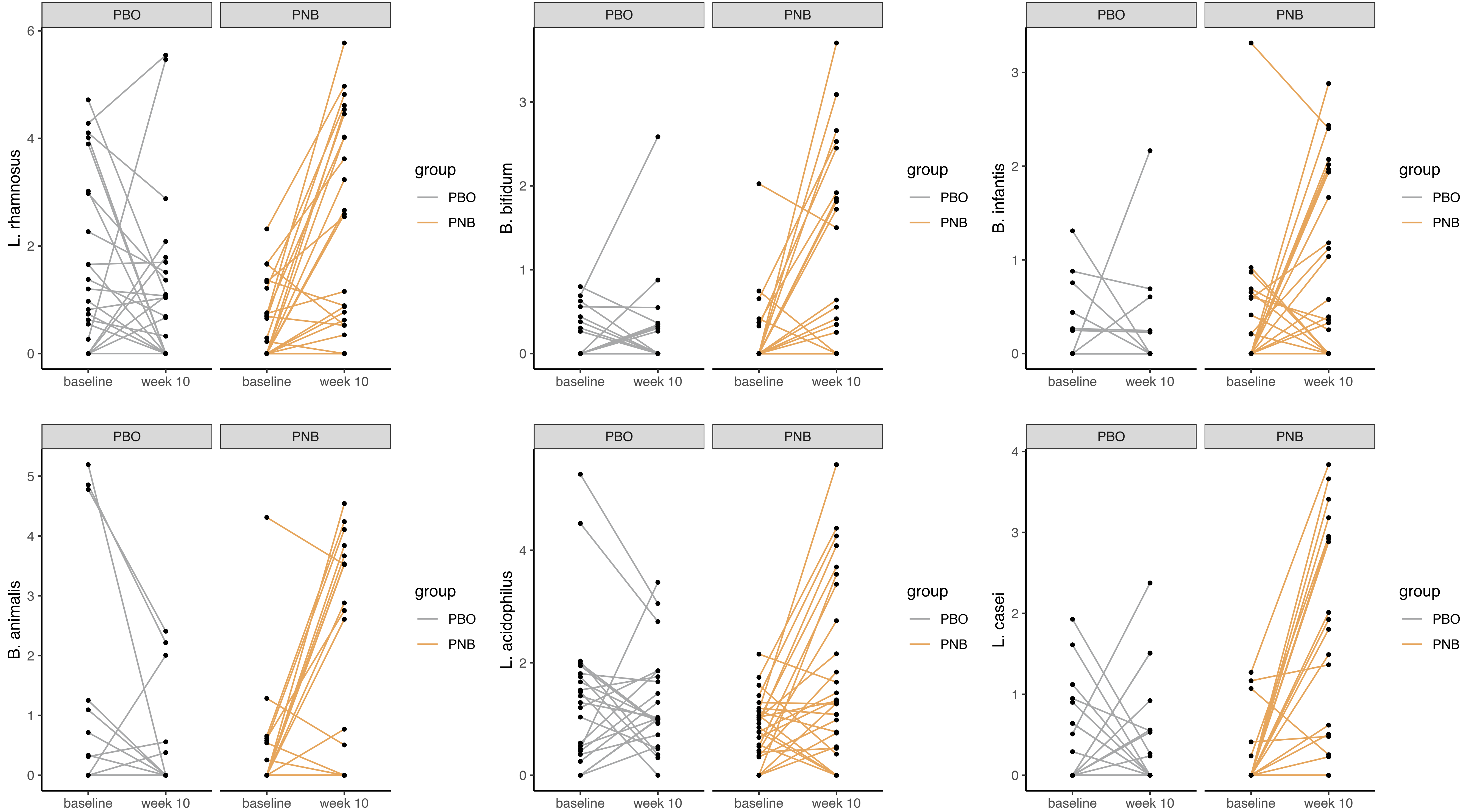


**Supplemental Figure S14:** Abundances all six supplemented probiotics from baseline to week 10

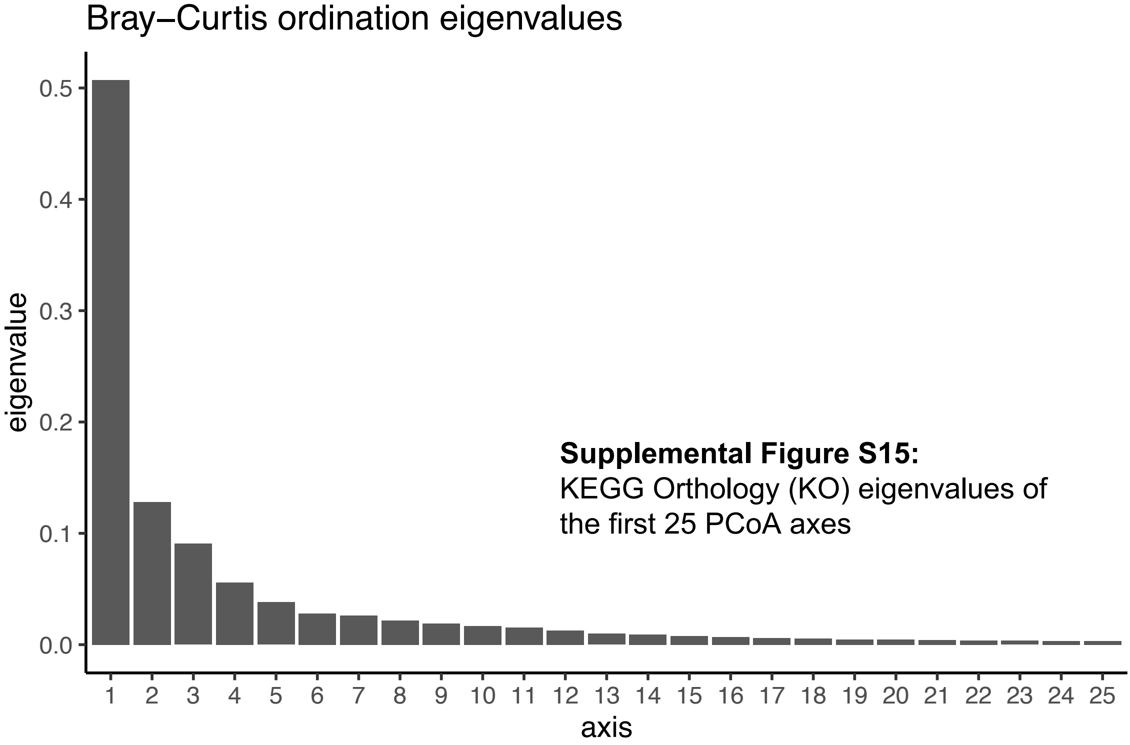


**Supplemental Figure S15:**
KEGG Orthology (KO) eigenvalues of the first 25 PCoA axes

**Supplemental Figure S16:** Principal coordinate analysis (PCoA) of the Kyoto Encyclopedia of Genes and Genomes Orthology (KO) terms

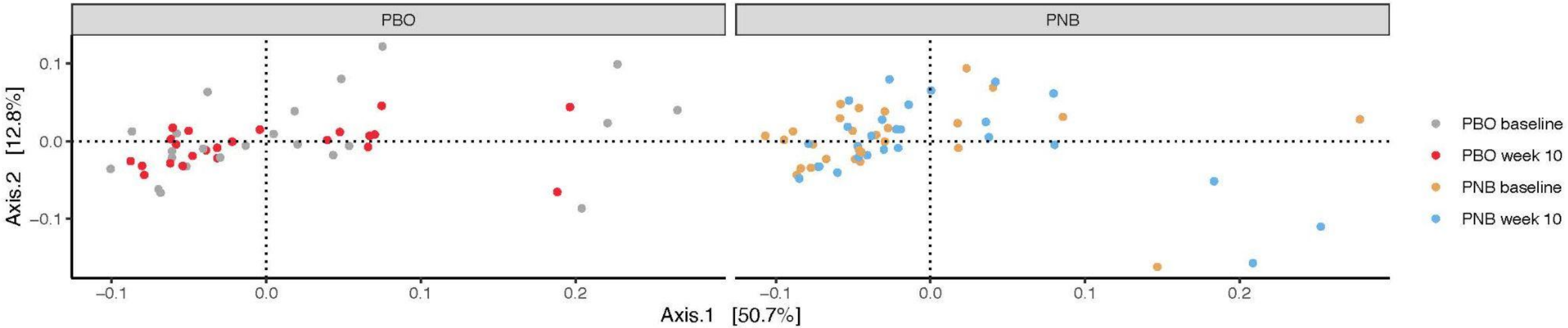


The abundances of reads mapped to the 3,867 Kyoto Encyclopedia of Genes and Genomes Orthology (KO) terms were log-transformed and Principal coordinate analysis (PCoA) was performed. The first and second axes explained 50.7% and 12.8% of the overall variance, respectively, with only the first three PCoA axes explaining >6% of the variance. Along the first two PCoA axes, no spatial separation was observed between the two time points within either of the two treatment groups (PERMANOVA with or without the group*time point interaction term using the Bray-Curtis dissimilarity matrix). When each PCoA axis was examined individually, no significant shift was observed along PCoA1 through PCoA3 axes in any of the groups. PBO = Placebo group; PNB = Probiotic and Nutraceutical blend group.

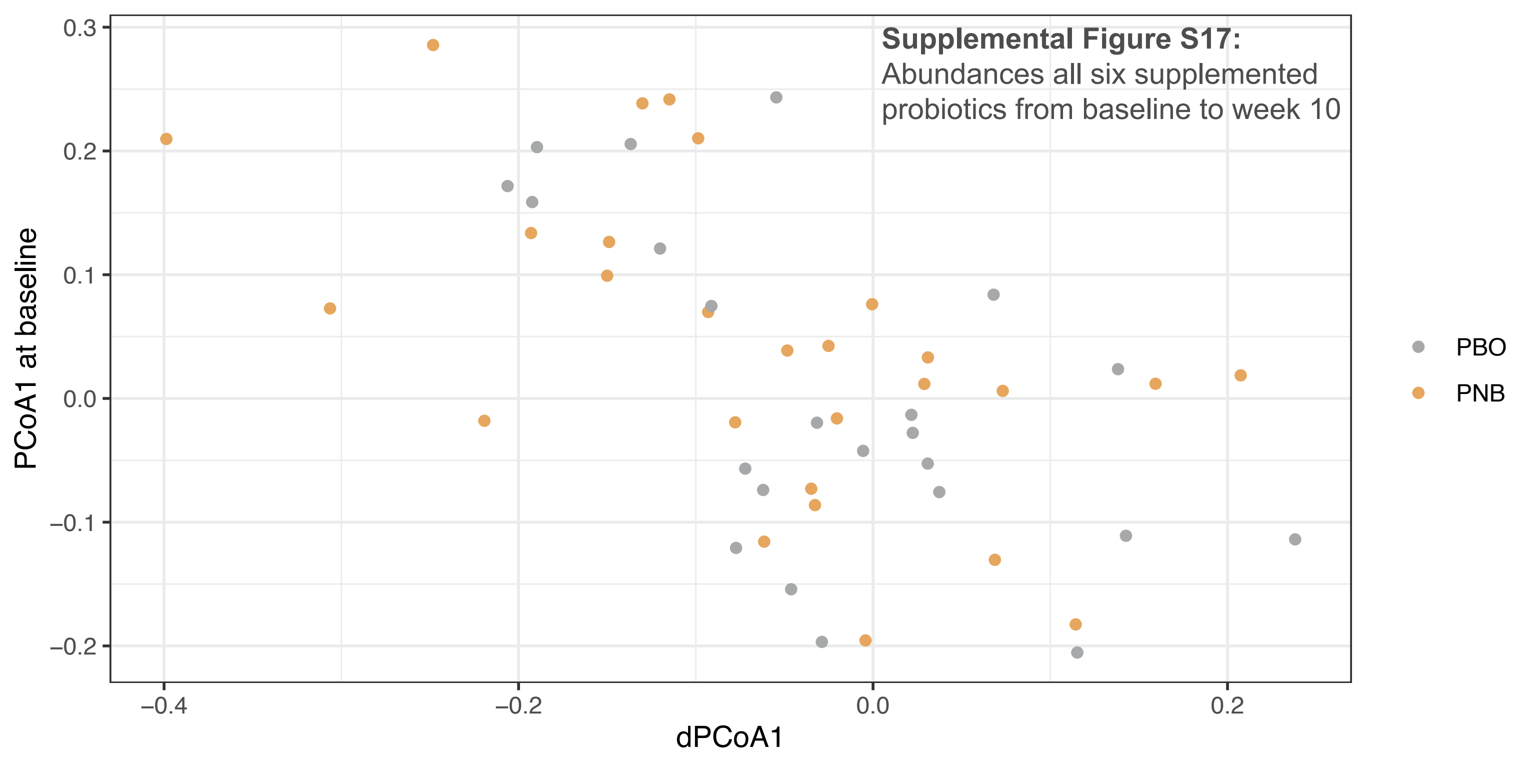
Supplemental Figure S17:
Abundances all six supplemented probiotics from baseline to week 10
PCoA1 at baseline
dPCoA1
PBO
PNB
0.3
0.2
0.1
0.0
−0.1
−0.2
−0.4
−0.2
0.0
0.2

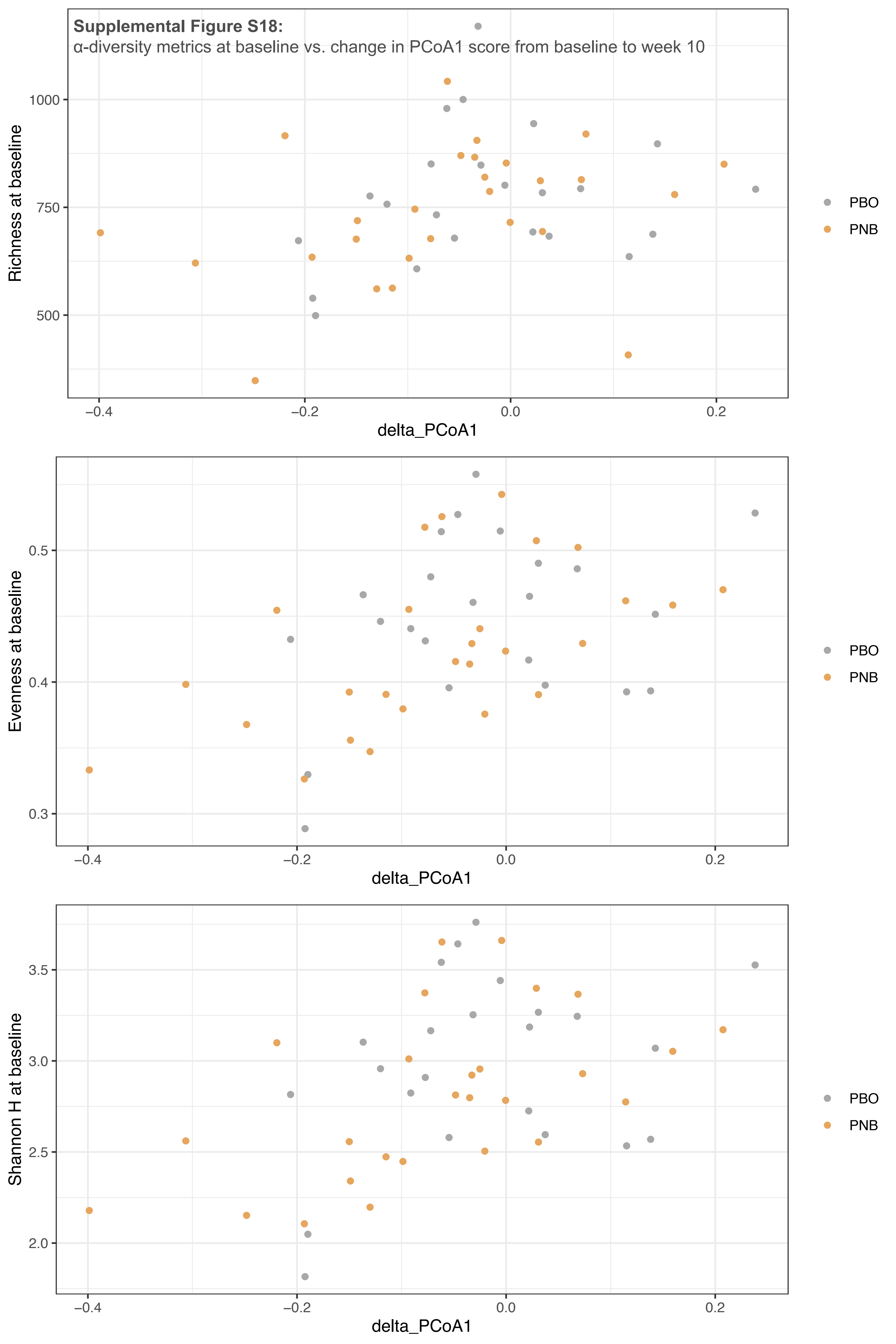
Supplemental Figure S18:
α-diversity metrics at baseline vs. change in PCoA1 score from baseline to week 10
Richness at baseline
1000
750
500
−0.4
−0.2
0.0
0.2
delta_PCoA1
PBO
PNB
Evenness at baseline
0.5
0.4
0.3
−0.4
−0.2
0.0
0.2
delta_PCoA1
PBO
PNB
Shannon H at baseline
3.5
3.0
2.5
2.0
−0.4
−0.2
0.0
0.2
delta_PCoA1
PBO
PNB

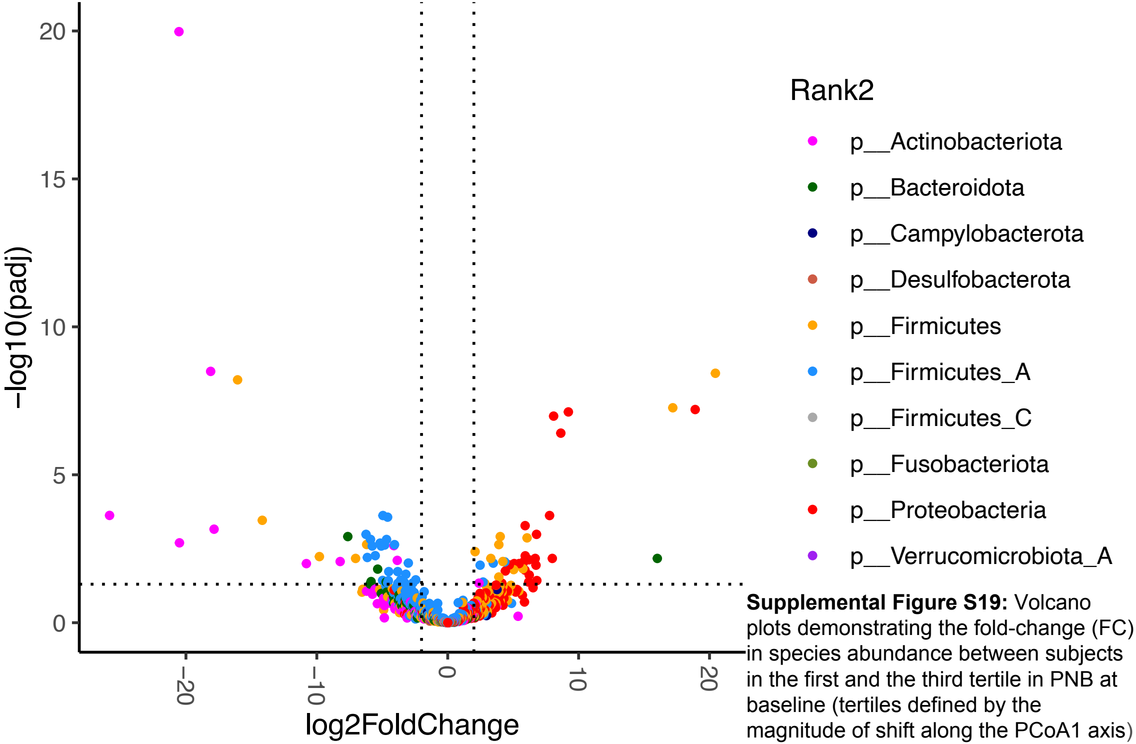


**Supplemental Figure S19:** Volcano plots demonstrating the fold-change (FC) in species abundance between subjects in the first and the third tertile in PNB at baseline (tertiles defined by the magnitude of shift along the PCoA1 axis)

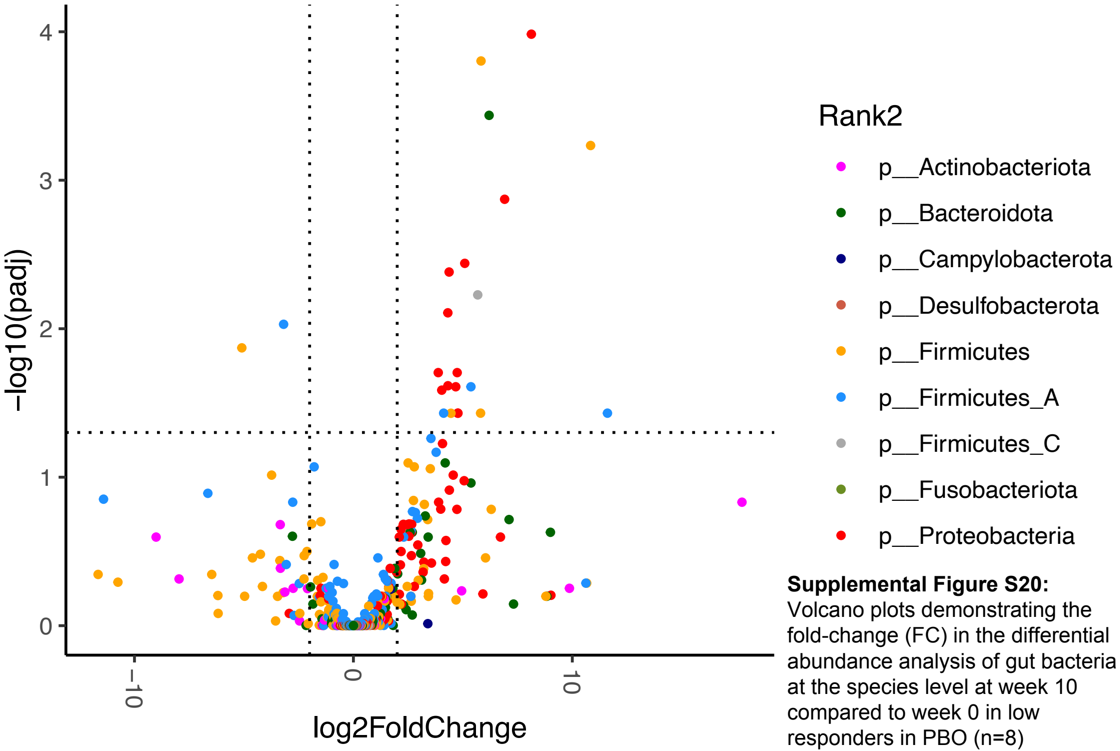


**Supplemental Figure S20:** Volcano plots demonstrating the fold-change (FC) in the differential abundance analysis of gut bacteria at the species level at week 10 compared to week 0 in low responders in PBO (n=8)

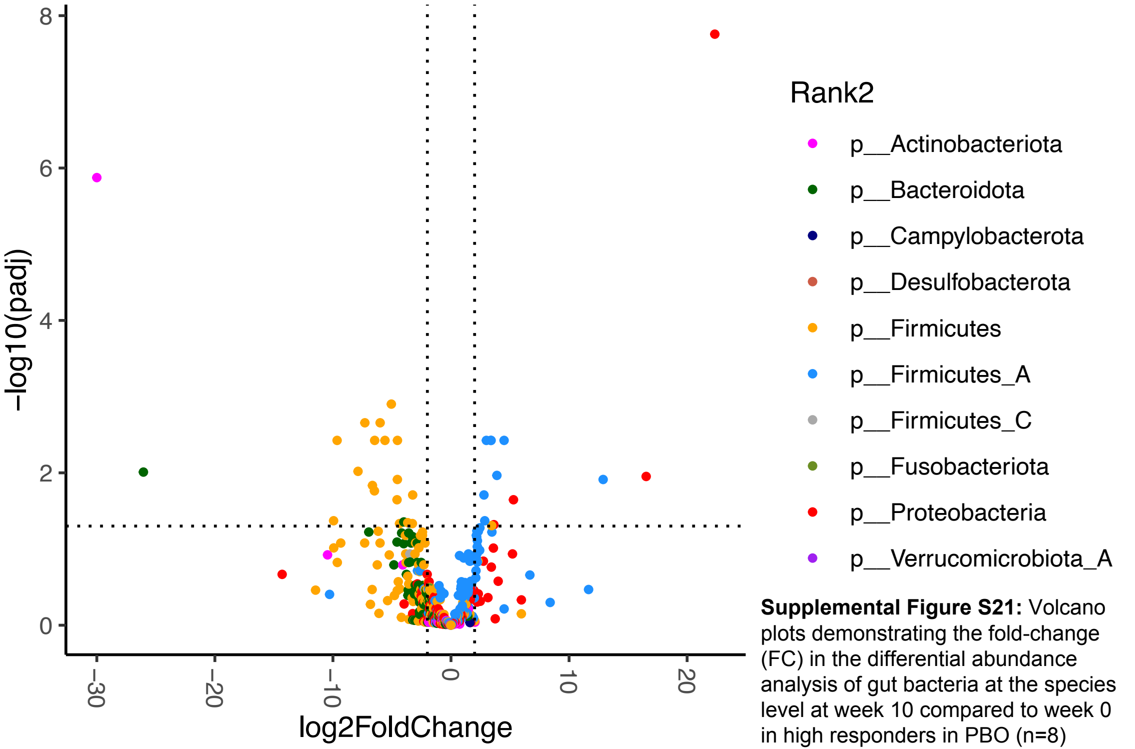


**Supplemental Figure S21:** Volcano plots demonstrating the fold-change (FC) in the differential abundance analysis of gut bacteria at the species level at week 10 compared to week 0 in high responders in PBO (n=8)

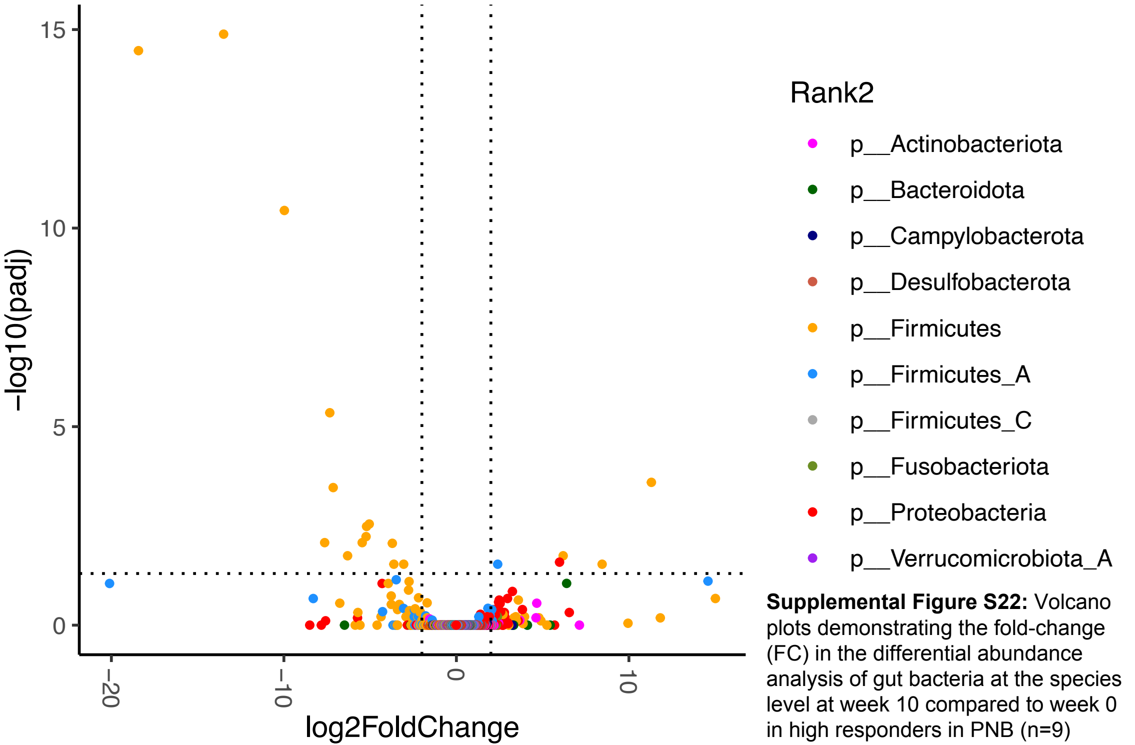


**Supplemental Figure S22:** Volcano plots demonstrating the fold-change (FC) in the differential abundance analysis of gut bacteria at the species level at week 10 compared to week 0 in high responders in PNB (n=9)

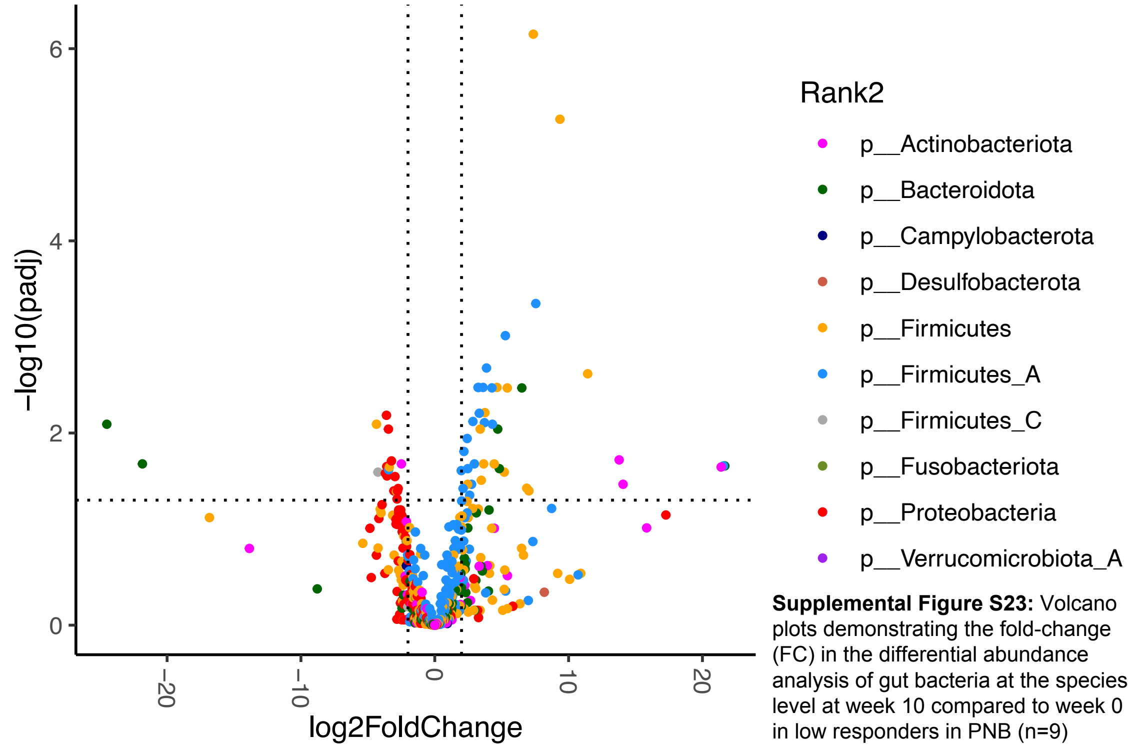


**Supplemental Figure S23:** Volcano plots demonstrating the fold-change (FC) in the differential abundance analysis of gut bacteria at the species level at week 10 compared to week 0 in low responders in PNB (n=9)

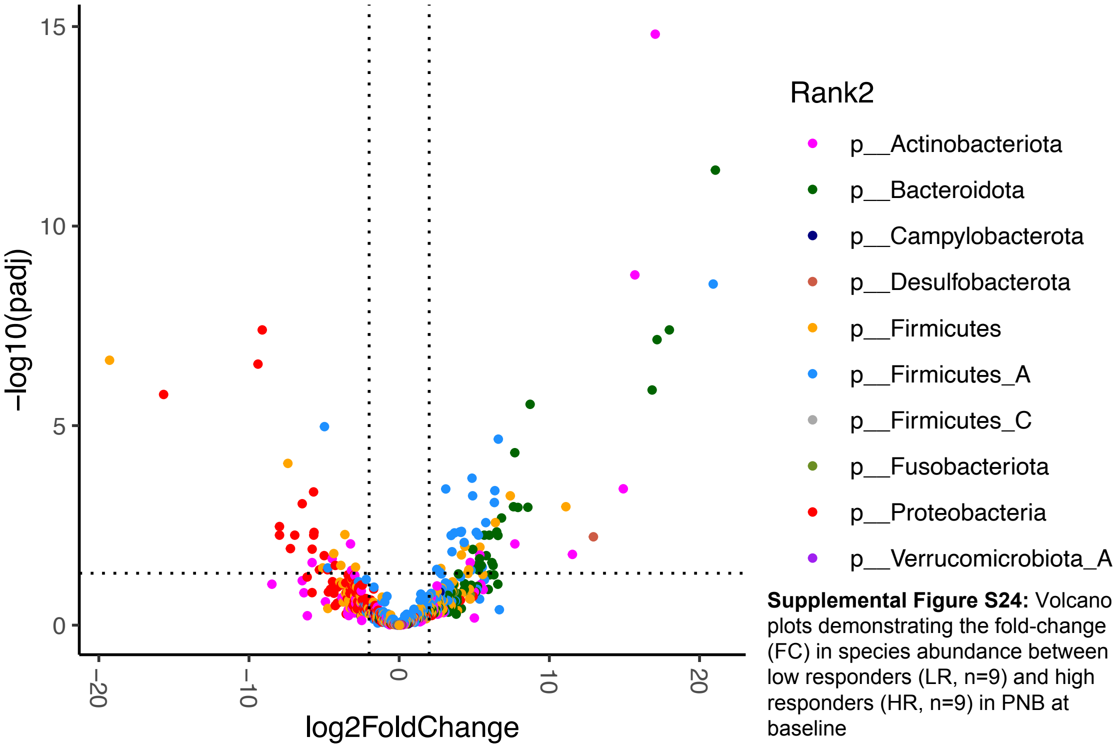


**Supplemental Figure S24:** Volcano plots demonstrating the fold-change (FC) in species abundance between low responders (LR, n=9) and high responders (HR, n=9) in PNB at baseline

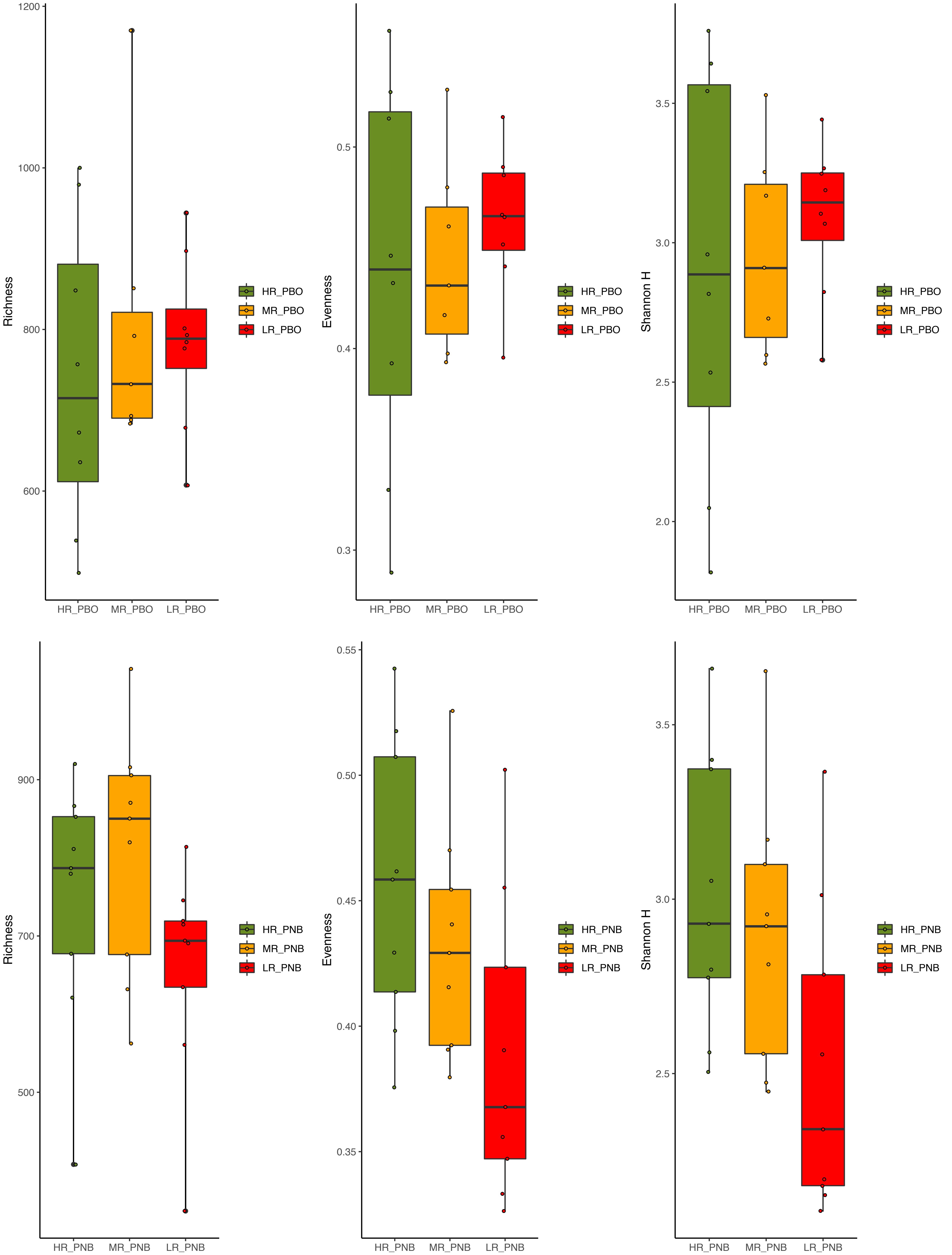


**Supplemental Figure S25:** The α-diversity metrics between HR, MR, and LR at baseline in the both groups

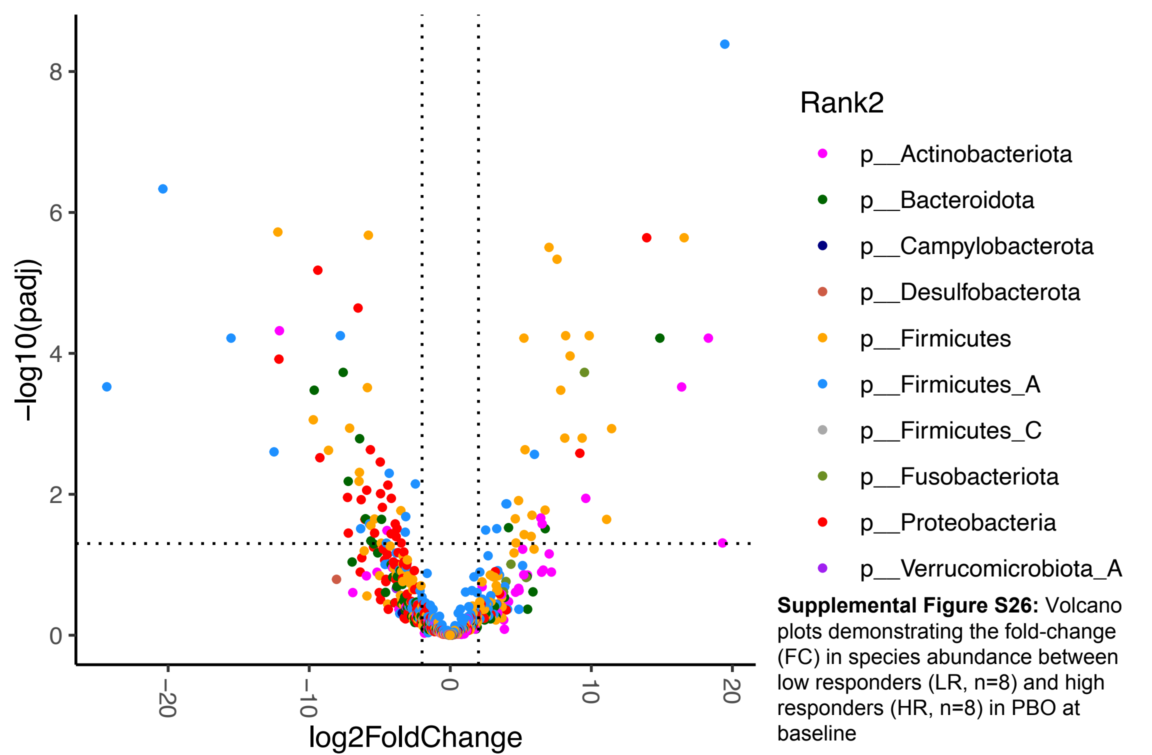


**Supplemental Figure S26:** Volcano plots demonstrating the fold-change (FC) in species abundance between low responders (LR, n=8) and high responders (HR, n=8) in PBO at baseline
