# Supplementary material for: A Randomized Controlled Trial to Evaluate the Impact of a Novel Probiotic and Nutraceutical Supplement on Pruritic Dermatitis and the Gut Microbiota in Privately Owned Dogs
Source: Animals (Basel). 2024 Jan 30;14(3):453. doi: 10.3390/ani14030453 (PMC10854619; doi:10.3390/ani14030453)
Supplement: Supplementary file 1 [file animals-14-00453-s001.zip › Supplementary Tables.pdf]

Supplemental Table S1 - Eligibility Criteria

Subject Characteristics	● aged 1-12 years<br>● body condition score (BCS) of 4-6 (ideal to overweight but not obese)<br>● 22.7 kg or less<br>● no restrictions on: breed, sex, or spay/neuter status
Pruritic Dermatitis	● presence of seasonal or nonseasonal pruritic dermatitis determined by:<br>○ compatible history, clinical signs or veterinary indication<br>○ absence of bacterial pyoderma and *Malassezia* dermatitis, uncontrolled flea allergy dermatitis, seborrhea, sarcoptic mange, demodicosis, dermatophytosis, cheyletiellosis, or any additional ectoparasitic disorders, presence of mites or louse<br>○ absence of adverse food reactions or improvement of clinical signs with diet<br>○ lack of gastrointestinal (GI) issues<br>○ onset of clinical signs post one year of age.
Health Status	● absence of any concurrent systemic disease including:<br>○ diabetes<br>○ hypothyroidism<br>○ hyperadrenocorticism<br>○ pancreatitis<br>○ kidney disease<br>○ liver disease<br>○ heart disease<br>○ cancer<br>● not pregnant or lactating<br>● having no surgery within the last 3 months
Medications & Supplements	● permitted (given dogs were using them for at least one month prior to the trial, had persisting visible skin allergy clinical signs, and agreed to adhere to their dose and frequency regime throughout the study period):<br>○ steroids<br>○ antihistamines<br>○ immunosuppressants<br>○ Apoquel (oclacitinib)<br>○ Cytopoint (lokivetmab) and allergen immunotherapy<br>○ sublingual immunotherapy

	○ topical products<br>○ medicated hypo-allergic shampoos and wipes<br>● not permitted (within one month of the trial or at any point):<br>○ oral antibiotics, antifungals, or antiparasitics (except regular flea preventatives)<br>○ dietary supplements containing:<br>■ probiotics<br>■ prebiotics<br>■ yeast fermentate<br>■ vitamin E<br>■ B vitamins<br>■ vitamin A or carotenoids<br>■ fatty acids<br>■ plant extracts
Diet	● consuming any combination of four commercially available fresh canine diets (NomNomNow, Inc., Nashville, TN, USA) for one month prior to the start of the trial and throughout the study period<br>● no restriction on treat intake

Supplemental Table S2 - Characteristics of each participant (n=63*)

Group	Breed	Weight (kg)	Medications	Region[a]	Included in survey analysis?	Included in MB analysis?	Spay/neuter	BCS[b]	Age	Sex	Coat Type	Flea Medications	Pruritus Onset[c]	Seasonal
PNB	French Bulldog	10.9	Oclacitinib, Lokivetmab	south	YES	YES	TRUE	6	2.6	female	Short-coated	FALSE	YOUNG	FALSE
PNB	Beagle	18.1	None	south	YES	YES	TRUE	6	9.4	female	Short-coated	FALSE	YOUNG	FALSE
PNB	Havanese	6.8	None	north	YES	YES	TRUE	4-5	7.1	male	Curly-coated	TRUE	ADULT	FALSE
PNB	Shih Poo	7.7	Allergen immunotherapy (hypo-sensitization/allergy shots)	north	YES	YES	TRUE	4-5	7.6	male	Curly-coated	TRUE	ADULT	FALSE
PNB	Bichon Frise And Poodle	5.4	Lokivetmab	midwest	YES	YES	TRUE	4-5	7.6	male	Curly-coated	TRUE	ADULT	FALSE
PNB	Poodle (miniature)	2.7	Antihistamines (i.e. Diphenhydramine, Cetirizine, Loratadine, Chlorpheniramine)	south	YES	YES	FALSE	4-5	11	female	Curly-coated	TRUE	MATURE	FALSE
PNB	Mixed Breed	7.7	None	west	YES	YES	TRUE	4-5	6.7	male	Medium coated	FALSE	YOUNG	FALSE
PNB	Miniature Schnauzer	5.0	Oclacitinib	south	YES	YES	TRUE	4-5	3.8	male	Medium coated	FALSE	YOUNG	FALSE
PNB	Dachshund, Yorkie, Shihtzu Mix	6.4	Medicated and hypo-allergenic shampoos and wipes	west	YES	NO	TRUE	4-5	9.3	male	Long-coated	FALSE	ADULT	FALSE
PNB	Pomeranian	2.7	None	west	YES	NO	TRUE	4-5	1.4	male	Long-coated	FALSE	YOUNG	TRUE
PNB	Maltese	2.7	None	west	YES	YES	TRUE	4-5	4.6	male	Curly-coated	TRUE	YOUNG	FALSE
PNB	Mix	15.9	None	west	YES	YES	TRUE	4-5	9.3	male	Medium coated	TRUE	YOUNG	FALSE
PNB	Shih Poo	5.4	Oclacitinib	north	YES	NO	TRUE	4-5	8	female	Curly-coated	TRUE	YOUNG	TRUE
PNB	French Bulldog	11.8	Antihistamines (i.e. Diphenhydramine, Cetirizine, Loratadine, Chlorpheniramine), Medicated and hypo-allergenic shampoos and wipes	south	YES	YES	TRUE	6	7.3	female	Short-coated	FALSE	YOUNG	FALSE
PNB	Maltese	4.1	Oclacitinib	north	YES	YES	TRUE	4-5	12.5	female	Short-coated	TRUE	MATURE	FALSE
PNB	NA	3.2	None	west	YES	YES	TRUE	4-5	8.7	female	Short-coated	FALSE	YOUNG	TRUE
PNB	Dalmatian	19.5	None	west	YES	NO	TRUE	4-5	6.3	female	Short-coated	FALSE	YOUNG	FALSE
PNB	Chihuahua	5.4	None	north	YES	NO	TRUE	4-5	8.6	male	Short-coated	TRUE	YOUNG	FALSE
PNB	Poodle Bichon	10.0	Allergen immunotherapy (hypo-sensitization/allergy shots)	west	YES	YES	TRUE	6	7.4	male	Curly-coated	FALSE	YOUNG	FALSE
PNB	Chihuahua	9.1	Medicated and hypo-allergenic shampoos and wipes, Oclacitinib	south	YES	YES	TRUE	4-5	7.8	female	Wire-coated	FALSE	ADULT	FALSE
PNB	Cockapoo	8.2	Lokivetmab	south	YES	YES	TRUE	4-5	11.8	male	Curly-coated	TRUE	ADULT	FALSE
PNB	Beagle	12.7	Medicated and hypo-allergenic shampoos and wipes, Topicals (i.e. Nystatin-neomycin sulfate-thiostrepton-triamcinolone acetonide, Betamethasone), Antihistamines (i.e. Diphenhydramine, Cetirizine, Loratadine, Chlorpheniramine)	north	YES	YES	TRUE	4-5	4	female	Short-coated	TRUE	YOUNG	TRUE

PNB	Shih Tzu	5.4	Steroids (i.e. Prednisone, Dexamethasone, Hydrocortisone, Triamcinolone), Antihistamines (i.e. Diphenhydramine, Cetirizine, Loratadine, Chlorpheniramine), Medicated and hypo-allergenic shampoos and wipes, Allergen immunotherapy (hypo-sensitization/allergy shots)	north	YES	YES	TRUE	4-5	10.9	male	Long-coated	TRUE	MATURE	FALSE
PNB	Jack Russel Terrier Mix	5.4	Oclacitinib, Allergen immunotherapy (hypo-sensitization/allergy shots), Other	south	YES	YES	TRUE	4-5	9.6	female	Short-coated	TRUE	YOUNG	TRUE
PNB	Chihuahua	4.5	None	midwest	YES	YES	TRUE	4-5	14.4	male	Medium coated	FALSE	MATURE	FALSE
PNB	Pug	17.7	Antihistamines (i.e. Diphenhydramine, Cetirizine, Loratadine, Chlorpheniramine)	north	YES	YES	TRUE	6	6.4	female	Short-coated	TRUE	YOUNG	FALSE
PNB	Border Collie	22.2	Oclacitinib	south	YES	YES	TRUE	4-5	6.8	female	Medium coated	TRUE	ADULT	FALSE
PNB	Shihpoo	8.6	Allergen immunotherapy (hypo-sensitization/allergy shots)	south	YES	YES	TRUE	4-5	2.2	male	Curly-coated	FALSE	YOUNG	FALSE
PNB	Yorkiepoo	5.9	Medicated and hypo-allergenic shampoos and wipes	west	YES	YES	TRUE	4-5	13.6	male	Medium coated	FALSE	MATURE	FALSE
PNB	N/A	17.7	Oclacitinib, Medicated and hypo-allergenic shampoos and wipes	south	YES	YES	TRUE	4-5	3.6	female	Short-coated	FALSE	YOUNG	FALSE
PNB	Jack Russell Chihuahua Mix	5.9	None	west	YES	YES	TRUE	4-5	8.8	female	Short-coated	FALSE	ADULT	FALSE
PNB	Mini Australian Shepard	14.5	None	west	YES	NO	TRUE	4-5	2.9	female	Medium coated	TRUE	YOUNG	FALSE
PNB	Yorkshire Terrier	3.2	Medicated and hypo-allergenic shampoos and wipes	south	YES	NO	TRUE	4-5	5.7	male	Long-coated	TRUE	YOUNG	FALSE
PNB	Boston Terrier	17.7	Oclacitinib, Medicated and hypo-allergenic shampoos and wipes	north	NO	YES	TRUE	6	8.3	male	Short-coated	FALSE	YOUNG	FALSE
PBO	Chihuahua	4.1	Antihistamines (i.e. Diphenhydramine, Cetirizine, Loratadine, Chlorpheniramine), Medicated and hypo-allergenic shampoos and wipes	west	YES	YES	TRUE	4-5	8.3	male	Short-coated	TRUE	ADULT	FALSE
PBO	Goldendoodle	20.4	Oclacitinib	south	YES	YES	TRUE	4-5	2.7	female	Curly-coated	TRUE	YOUNG	FALSE
PBO	NA	20.4	Allergen immunotherapy (hypo-sensitization/allergy shots)	west	YES	YES	TRUE	4-5	8.2	female	Medium coated	FALSE	ADULT	FALSE
PBO	Shih Tzu	5.4	Antihistamines (i.e. Diphenhydramine, Cetirizine, Loratadine, Chlorpheniramine)	south	YES	YES	TRUE	4-5	11.5	female	Short-coated	TRUE	MATURE	FALSE
PBO	Yorkie pomerian	6.8	None	west	YES	NO	TRUE	6	4.2	female	Medium coated	TRUE	YOUNG	FALSE
PBO	Mixed Breed	6.8	Antihistamines (i.e. Diphenhydramine, Cetirizine, Loratadine, Chlorpheniramine)	south	YES	NO	TRUE	4-5	4.4	male	Short-coated	TRUE	YOUNG	TRUE
PBO	Miniature Schnauzer	7.7	None	west	YES	YES	TRUE	4-5	7.4	female	Wire-coated	FALSE	YOUNG	TRUE
PBO	Australian Shepherd	8.6	Medicated and hypo-allergenic shampoos and wipes	south	YES	NO	TRUE	4-5	7	male	Long-coated	TRUE	ADULT	FALSE
PBO	Mixed Breed	16.8	None	north	YES	YES	TRUE	4-5	1.8	female	Medium coated	FALSE	YOUNG	FALSE
PBO	Shih Tzu	7.3	None	north	YES	YES	TRUE	4-5	8	female	Medium coated	TRUE	ADULT	FALSE
PBO	Maltese	5.9	Oclacitinib, Antihistamines (i.e. Diphenhydramine, Cetirizine, Loratadine, Chlorpheniramine), Medicated and hypo-allergenic shampoos and	north	YES	YES	TRUE	6	12.9	male	Medium coated	FALSE	MATURE	FALSE

			wipes											
PBO	Pitbull Mix	21.8	Medicated and hypo-allergenic shampoos and wipes	midwest	YES	YES	TRUE	4-5	11.7	female	Short-coated	TRUE	MATURE	FALSE
PBO	NA	3.6	None	west	YES	YES	TRUE	4-5	5.8	female	Curly-coated	FALSE	YOUNG	FALSE
PBO	Australian Cattle Dog	21.8	Topicals (i.e. Nystatin-neomycin sulfate-thiostrepton-triamcinolone acetonide, Betamethasone), Medicated and hypo-allergenic shampoos and wipes, Oclacitinib	south	YES	YES	TRUE	4-5	5.3	male	Medium coated	TRUE	ADULT	FALSE
PBO	Chihuahua	3.6	Oclacitinib	west	YES	NO	TRUE	4-5	15.3	male	Short-coated	TRUE	ADULT	FALSE
PBO	Mix	22.7	Oclacitinib	west	YES	YES	TRUE	4-5	9.2	male	Short-coated	TRUE	YOUNG	FALSE
PBO	Labradoodle	10.9	Lokivetmab	south	YES	YES	TRUE	4-5	3.4	female	Curly-coated	FALSE	YOUNG	FALSE
PBO	Miniature Pinscher	7.3	Antihistamines (i.e. Diphenhydramine, Cetirizine, Loratadine, Chlorpheniramine)	west	YES	YES	TRUE	4-5	9	female	Short-coated	FALSE	YOUNG	FALSE
PBO	Yorkshire Terrier	2.7	None	midwest	YES	YES	TRUE	6	10.9	male	Long-coated	FALSE	ADULT	FALSE
PBO	Shih Tzu	7.7	None	south	YES	YES	TRUE	4-5	6.8	male	Medium coated	TRUE	YOUNG	FALSE
PBO	Chihuahua	2.7	None	midwest	YES	YES	TRUE	4-5	12.3	female	Short-coated	FALSE	ADULT	TRUE
PBO	Tibetan Terrier Cocker Spaniel	8.6	Antihistamines (i.e. Diphenhydramine, Cetirizine, Loratadine, Chlorpheniramine)	midwest	YES	YES	TRUE	4-5	5.5	female	Medium coated	FALSE	YOUNG	FALSE
PBO	Poodle (toy)	4.5	Oclacitinib	south	YES	NO	TRUE	4-5	8.8	female	Curly-coated	TRUE	YOUNG	FALSE
PBO	Yorkshire Terrier	5.9	None	south	YES	YES	TRUE	4-5	7	female	Short-coated	FALSE	ADULT	FALSE
PBO	Lhasa Apso	13.6	Steroids (i.e. Prednisone, Dexamethasone, Hydrocortisone, Triamcinolone), Antihistamines (i.e. Diphenhydramine, Cetirizine, Loratadine, Chlorpheniramine)	north	YES	YES	TRUE	4-5	10	male	Medium coated	TRUE	ADULT	FALSE
PBO	Papillon Mutt	13.2	None	west	YES	YES	TRUE	6	10	female	Long-coated	FALSE	YOUNG	FALSE
PBO	Mixed Breed	18.1	Steroids (i.e. Prednisone, Dexamethasone, Hydrocortisone, Triamcinolone), Antihistamines (i.e. Diphenhydramine, Cetirizine, Loratadine, Chlorpheniramine), Medicated and hypo-allergenic shampoos and wipes, Allergen immunotherapy (hypo-sensitization/allergy shots)	west	YES	NO	TRUE	4-5	0.9	male	Medium coated	TRUE	YOUNG	FALSE
PBO	NA	7.7	None	south	YES	YES	TRUE	4-5	3.3	male	Short-coated	TRUE	YOUNG	FALSE
PBO	Yorkshire Terrier	4.1	Oclacitinib	south	YES	YES	TRUE	4-5	8.9	male	Short-coated	FALSE	ADULT	FALSE

* n=63 dogs (n=62 included in survey analysis, n=50 included in microbiome analysis; 1 dog was included in microbiome analysis and not survey analysis; 13 dogs were included in survey analysis and not microbiome analysis)

[a] Regions (Continental US): **north** = ME, NH, VT, MA, RI, CT, NY, NJ, PA, DE; **south** = MD, WV, VA, KY, NC, SC, TN, GA, FL, AL, MS, LA, AS, OK, TX; **midwest** = MO, KS, NE, SD, ND, MN, IA, WI, IL, IN, MI, OH; **west** = NM, CO, WY, MT, ID, UT, AZ, NV, WA, OR, CA

[b] 9-point scale; Body Condition Score (BCS): 4-5 (ideal weight), 6 (slightly overweight)

[c] YOUNG = 1-3 years old; ADULT = 3 - 7 years old; MATURE = >7 years old

Supplemental Table S3: Median canine pruritus severity scores (digital PVAS10) at baseline and week 10

	Week 0			Week 10		
	Min	Max	Median	Min	Max	Median
PBO (n=29)	3.84	8.28	**6.28**	0.03	10.00	**3.99**
PNB (n=33)	1.66	10.00	**6.02**	0.00	7.72	**3.86**

Supplemental Table S4: Percent change OA-SASI from baseline as ranked improvement score

	PBO (n=29)	PNB (n=33)	***p*** value*
**Week 2**			0.087
Score 0	11 (37%)	3 (9%)	
Score 1	4 (14%)	9 (27%)	
Score 2	6 (21%)	7 (21%)	
Score 3	6 (21%)	9 (27%)	
Score 4	2 (7%)	5 (16%)	
**Week 4**			0.363
Score 0	7 (24%)	7 (21%)	
Score 1	4 (14%)	7 (21%)	
Score 2	1 (4%)	1 (4%)	
Score 3	12 (41%)	7 (21%)	
Score 4	5 (17%)	11 (33%)	
**Week 7**			0.343
Score 0	4 (14%)	5 (16%)	
Score 1	6 (21%)	6 (18%)	
Score 2	4 (14%)	2 (6%)	
Score 3	3 (10%)	10 (30%)	
Score 4	12 (41%)	10 (30%)	
**Week 10**			1.000
Score 0	7 (24%)	8 (24%)	
Score 1	3 (10%)	3 (9%)	
Score 2	4 (14%)	5 (16%)	
Score 3	6 (21%)	6 (18%)	
Score 4	9 (31%)	11 (33%)	

* Fisher's Exact Test

Score 0 = increased CADESI; score 1 = <25% reduction; score 2 = 25–49% reduction; score 3 = 50–74% reduction; score 4 = ≥75% reduction

Supplemental Table S5: OA-SASI scores at week 2 by lesion and body site

	PBO (n=29)	PNB (n=33)	***p*** **value***
**Alopecia/excoriation**	2.28 ± 2.4	1.18 ± 1.9	**0.030**
Face	0.28 ± 0.5	0.21 ± 0.5	0.431
Ears	0.28 ± 0.6	0.06 ± 0.2	0.083
Paws	0.59 ± 0.7	0.27 ± 0.6	**0.033**
Limbs	0.52 ± 0.7	0.30 ± 0.6	0.123
Underside	0.62 ± 0.8	0.33 ± 0.7	0.068
**Erythema**	3.72 ± 2.4	2.09 ± 1.5	**0.005**
Face	0.62 ± 0.7	0.30 ± 0.5	0.066
Ears	0.55 ± 0.7	0.24 ± 0.4	0.061
Paws	1.14 ± 0.9	0.55 ± 0.7	**0.007**
Limbs	0.62 ± 0.8	0.45 ± 0.6	0.528
Underside	0.79 ± 0.8	0.55 ± 0.7	0.221
**Lichenification**	1.10 ± 1.6	0.33 ± 0.8	**0.039**
Face	0.17 ± 0.4	0.12 ± 0.3	0.579
Ears	0.14 ± 0.4	0.00 ± 0.0	**0.030**
Paws	0.24 ± 0.5	0.06 ± 0.3	**0.036**
Limbs	0.24 ± 0.5	0.09 ± 0.3	0.192
Underside	0.31 ± 0.6	0.06 ± 0.2	**0.042**

* Wilcoxon rank sum test

Supplemental Table S6 - Week 10 Health & Behavioral Outcomes

	PBO (n=29)	PNB (n=33)	***p*** **value***
Overall Health[a]	**8.55 ± 0.95**	**8.79 ± 0.99**	**0.260**
Better	12 (41%)	12 (36%)	0.500
No change	12 (41%)	11 (34%)	
Worse	5 (18%)	10 (30%)	
Overall Stress[b]	**1.28 ± 0.96**	**1.12 ± 0.86**	**0.463**
Better	13 (45%)	10 (30%)	0.065
No change	15 (52%)	15 (46%)	
Worse	1 (3%)	8 (24%)	
Overall Energy[c]	**2.35 ± 0.97**	**2.30 ± 0.68**	**0.835**
Better	6 (21%)	4 (12%)	0.609
No change	20 (69%)	27 (82%)	
Worse	3 (10%)	2 (6%)	
Quality of Life[d]	**9.07 ± 0.92**	**9.09 ± 1.1**	**0.702**
Better	6 (21%)	7 (21%)	1.000
No change	15 (52%)	17 (52%)	
Worse	8 (27%)	9 (27%)	
Overall Skin Condition[e]	**3.76 ± 2.8**	**3.70 ± 3.4**	**0.728**
Better	17 (59%)	16 (49%)	0.071
No change	1 (3%)	8 (24%)	
Worse	11 (38%)	9 (27%)	
Overall Hair Loss[f]	**1.72 ± 2.4**	**1.15 ± 2.0**	**0.153**
Better	12 (41%)	12 (36%)	0.944
No change	12 (41%)	15 (46%)	
Worse	5 (18%)	6 (18%)	
Licking Amount[g]	**3.86 ± 3.0**	**3.55 ± 2.5**	**0.753**
Better	24 (83%)	22 (67%)	0.205
No change	2 (7%)	8 (24%)	
Worse	3 (10%)	3 (9%)	
Scratching Amount[h]	**3.90 ± 2.8**	**3.12 ± 2.6**	**0.221**
Better	24 (83%)	22 (67%)	0.192
No change	1 (3%)	6 (18%)	
Worse	4 (14%)	5 (15%)	
Redness Amount[i]	**2.62 ± 2.7**	**1.82 ± 2.4**	**0.163**
Better	22 (76%)	23 (70%)	0.774
No change	4 (14%)	7 (21%)	
Worse	3 (10%)	3 (9%)	
Household Disruption[j]	**3.86 ± 3.2**	**2.52 ± 2.6**	**0.073**
Better	15 (52%)	23 (70%)	0.200
No change	9 (30%)	4 (12%)	
Worse	5 (18%)	6 (18%)	
Fecal score[k]	2.73 ± 0.94	2.70 ± 0.73	0.865

Skin Type<br>Normal<br>Not Normal	 21 (72%)<br>8 (28%)	 24 (73%)<br>9 (27%)	1.000
Hours Outside (per day)<br>≤ 1 hour<br>> 1 hour	 10 (34%)<br>19 (66 %)	 19 (58%)<br>14 (42%)	0.081
Recent Bath[l]	18 (62%)	21 (64%)	1.000
Coat Description<br>Dry<br>Soft<br>Thin<br>Bald spots<br>Healthy<br>Shiny<br>Oily<br>Coarse<br>Thick<br>Hair loss<br>Unhealthy	 4 (14%)<br>21 (72%)<br>1 (3%)<br>1 (3%)<br>18 (62%)<br>8 (28%)<br>1 (3%)<br>4 (14%)<br>5 (18%)<br>2 (7%)<br>0 (0%)	 6 (18%)<br>23 (70%)<br>2 (6%)<br>1 (3%)<br>22 (67%)<br>16 (49%)<br>1 (3%)<br>7 (21%)<br>10 (30%)<br>1 (3%)<br>0 (0%)	 0.738<br>1.000<br>1.000<br>1.000<br>0.793<br>0.120<br>1.000<br>0.519<br>0.254<br>0.595<br>1.000

Data are expressed as **mean ± SD (bold)** or n (%)

* Wilcoxon rank sum test for **continuous variables (bold)** and Fisher's exact test for categorical

[a] Overall Health - 0 (very poor) to 10 (excellent).

[b] Overall Stress - 0 (not stressed) to 4 (very stressed).

[c] Overall Energy - 0 (very low) to 4 (very high).

[d] Quality of life refers to your pet's ability to meet their basic needs (eat, drink, move, relieve themselves) all without pain or discomfort, and further exhibit signs of enjoyment and happiness in their day-to-day life; 0 (very poor) to 10 (very high).

[e] Overall Skin Condition - 0 (very healthy) to 10 (extremely poor).

[f] Overall Hair Loss - 0 (no hair loss) to 10 (extreme hair loss).

[g] Licking Amount - 0 (not licking at all) to 10 (licking all the time).

[h] Scratching Amount - 0 (not scratching at all) to 10 (scratching all the time).

[i] Redness Amount - 0 (not red at all) to 10 (extremely red).

[j] How disruptive would you rate your pet's skin condition to your household; 0 (not disruptive at all) to 10 (very disruptive).

[k] Bristol Stool Form Scale; regular scores 2-5 (ideal 3-4)

[l] Since prior survey (3 weeks)

Supplemental Table S7a. KO terms with differential abundances between baseline and week 10 in PNB (n=27, log_2|fold change| ≥ 2 and FDR-adjusted p < 0.05)

KO term	Abbreviation	Description	Log 2 FC mean ± SE	Adjusted *P*-value[1]
**Increased at week 10 (4 terms)**				
K15327	pksC	polyketide biosynthesis malonyl-CoA-[acyl-carrier-protein] transacylase	3.89±0.87	2.31E-02
K16214	pezT	UDP-N-acetylglucosamine kinase [EC:2.7.1.176]	2.97±0.72	2.31E-02
K16649	glft1	rhamnopyranosyl-N-acetylglucosaminyl-diphospho-decaprenol beta-1,3/1,4-galactofuranosyltransferase [EC:2.4.1.287]	2.27±0.55	2.31E-02
K23997	nnr	ADP-dependent NAD(P)H-hydrate dehydratase / NAD(P)H-hydrate epimerase [EC:4.2.1.136 5.1.99.6]	2.10±0.54	4.12E-02
**Decreased at week 10 (1 terms)**				
K01457	atzF	allophanate hydrolase [EC:3.5.1.54]	-2.42±0.59	2.31E-02

FC: fold change, KO: KEGG Orthology, SE: standard error

[1] p values were adjusted with false discovery rate for multiple comparisons

Supplemental Table S7b. KEGG Enzymes with differential abundances between baseline and week 10 in PNB (n=27, log_2|fold change| ≥ 2 and FDR-adjusted p < 0.05)

EC number	Abbreviation	Description	Log 2 FC mean ± SE	Adjusted *P*-value[1]
**Increased at week 10 (7 terms)**				
2.6.1.-	bacF	bacilysin biosynthesis transaminase BacF	7.39±1.17	6.80E-07
2.7.1.176	pezT	UDP-N-acetylglucosamine kinase	3.20±0.75	1.12E-02

1.1.1.339	tll	dTDP-6-deoxy-L-talose 4-dehydrogenase (NAD+)	2.94±0.75	1.92E-02
1.3.8.16	sgcG	2-amino-4-deoxychorismate dehydrogenase	2.49±0.52	1.78E-03
2.4.1.287	glft1	rhamnopyranosyl-N-acetylglucosaminyl-diphospho-decaprenol beta-1,3/1,4-galactofuranosyltransferase	2.34±0.55	1.12E-02
2.3.3.3	E2.3.3.3	citrate (Re)-synthase	2.25±0.58	1.92E-02
3.4.-.-	cwlS	peptidoglycan DL-endopeptidase CwlS	2.15±0.54	1.92E-02
**Decreased at week 10 (3 terms)**				
3.4.21.-	esp, sigA, sepA	serine protease autotransporter	-2.94±0.74	1.92E-02
2.7.13.3	pgtB	two-component system, NtrC family, phosphoglycerate transport system sensor histidine kinase PgtB	-2.83±0.72	1.92E-02
3.5.1.54	atzF	allophanate hydrolase	-2.70±0.65	1.15E-02

FC: fold change, EC: Enzyme Commission, SE: standard error

[1] p values were adjusted with false discovery rate for multiple comparisons

Differential abundance analysis demonstrated an increase in the abundance of four KO terms and a decrease of one KO term from baseline to week 10 in the PNB group. Likewise, for KEGG enzymes the abundance of seven terms increased and three terms decreased at week 10. Both KO and enzyme analyses identified the two enzymes "UDP-N-acetylglucosamine kinase [EC:2.7.1.176]" and "rhamnopyranosyl-N-acetylglucosaminyl-diphospho-decaprenol beta-1,3/1,4-galactofuranosyltransferase [EC:2.4.1.287]" to be increased, as well as the enzyme "atzf (allophanate hydrolase [EC:3.5.1.54])" to be decreased at week 10. One KEGG module associated with enediyne biosynthesis was found to increase at week 10 ("C-1027 benzoxazolinate moiety biosynthesis, chorismate → benzoxazolinyl-CoA [M00826]", log2FC: 2.21±0.47, adjusted p: 7.85×10^{-04}). Consistently, the abundance of one pathway also increased at week 10 ("biosynthesis of enediyne antibiotics", log2FC: 2.03±0.46, adjusted p: 1.03×10^{-03}).

Supplemental Table S8a. KO terms with differential abundances between baseline and week 10 in PBO (n=23, $\log_2|$fold change$|$ ≥ 2 and FDR-adjusted p < 0.05)

KO term	Abbreviation	Description	Log 2 FC mean ± SE	Adjusted *P*-value[1]
**Increased at week 10 (5 terms)**				
K05358	quiA	quinate dehydrogenase (quinone) [EC:1.1.5.8]	7.85±1.84	2.73E-02
K08167	smvA, qacA, lfrA	MFS transporter, DHA2 family, multidrug resistance protein	4.36±1.06	3.02E-02
K00276	AOC3, AOC2, tynA	primary-amine oxidase [EC:1.4.3.21]	3.23±0.71	2.33E-02
K02616	paaX	phenylacetic acid degradation operon negative regulatory protein	2.82±0.67	2.81E-02
K02615	paaJ	3-oxo-5,6-didehydrosuberyl-CoA/3-oxoadipyl-CoA thiolase [EC:2.3.1.223 2.3.1.174]	2.76±0.65	2.73E-02

FC: fold change, KO: KEGG Orthology, SE: standard error

[1] p values were adjusted with false discovery rate for multiple comparisons

Supplemental Table S8b. KEGG Enzymes with differential abundances between baseline and week 10 in PBO (n=23, $\log_2|$fold change$|$ ≥ 2 and FDR-adjusted p < 0.05)

EC number	Abbreviation	Description	Log 2 FC mean ± SE	Adjusted *P*-value[1]
**Increased at week 10 (8 terms)**				
1.1.5.8	quiA	quinate dehydrogenase (quinone)	8.05±1.83	4.91E-03
2.5.1.140	sbnA	N-(2-amino-2-carboxyethyl)-L-glutamate synthase	5.71±1.22	2.52E-03
1.8.5.5	phsA, psrA	thiosulfate reductase / polysulfide reductase chain A	3.46±0.89	2.80E-02
5.3.3.18	paaG	2-(1,2-epoxy-1,2-dihydrophenyl)acetyl-CoA isomerase	3.44±0.74	2.52E-03
1.4.3.21	AOC3, AOC2,	primary-amine oxidase	3.21±0.75	6.97E-03

	tynA			
2.3.1.223	paaJ	3-oxo-5,6-didehydrosuberyl-CoA/3-oxoadipyl-CoA thiolase	3.20±0.68	2.52E-03
4.1.2.52	hpaI, hpcH	4-hydroxy-2-oxoheptanedioate aldolase	3.09±0.74	1.06E-02
2.3.1.174	paaJ	3-oxo-5,6-didehydrosuberyl-CoA/3-oxoadipyl-CoA thiolase	2.93±0.65	3.30E-03

FC: fold change, EC: Enzyme Commission, SE: standard error

[1] p values were adjusted with false discovery rate for multiple comparisons

In the placebo (PBO) group, five KO terms and eight enzymes were observed to be increased at week 10. Both comparisons identified the increase in the abundance of the enzymes “quinate dehydrogenase (quinone) [EC:1.1.5.8]”, “primary-amine oxidase [EC:1.4.3.21]”, and “3-oxo-5,6-didehydrosuberyl-CoA/3-oxoadipyl-CoA thiolase [EC:2.3.1.223 & EC:2.3.1.174]”. Two KEGG modules were found to increase (“hydroxyproline degradation, trans-4-hydroxy-L-proline → 2-oxoglutarate [M00948]”, log2FC: 10.72±2.36, adjusted-p: 1.64×10^{-03}; “staphyloferrin B biosynthesis, L-serine → staphyloferrin B [M00875]”, log2FC: 4.86±1.25, adjusted p: 1.01×10^{-02}) and one module was found to decrease at week 10 (“elloramycin biosynthesis, 8-demethyltetracenomycin C → elloramycin A [M00784]”, log2FC: -3.67±0.94, adjusted p: 1.01×10^{-02}). The KEGG pathway “fluorobenzoate degradation” was the only pathway found to increase in its abundance at week 10 (log2FC: 7.52±1.40, adjusted p:1.43×10^{-05}), while no pathway was found to decrease.

Supplemental Table S9. Species with differential abundances between subjects in the first and the third tertile in PNB at baseline (tertiles defined by the magnitude of shift along the PCoA1 axis, $\log_2$|fold change| ≥ 2 and FDR-adjusted p < 0.05)

Phylum	Class	Order	Family	Genus	Species	Relative abundance (%) Median [IQR]		Third tertile vs First tertile	
						First tertile	Third tertile	Log 2 FC mean ± SE	Adjusted *P*-value[1]
**Higher in the third tertile at baseline (42 species)**									
Firmicutes	Bacilli	Lactobacillales	Streptococcaceae	Streptococcus	urinalis	0.00E+00 [0.00E+00-0.00E+00]	0.00E+00 [0.00E+00-0.00E+00]	20.45±3.04	3.71E-09
Proteobacteria	Gammaproteobacteria	Enterobacterales	Pasteurellaceae	Haemophilus_A	sputorum	0.00E+00 [0.00E+00-0.00E+00]	0.00E+00 [0.00E+00-0.00E+00]	18.91±3.05	6.24E-08
Firmicutes	Bacilli	Lactobacillales	Lactobacillaceae	Lacticaseibacillus	saniviri	0.00E+00 [0.00E+00-0.00E+00]	0.00E+00 [0.00E+00-3.45E-07]	17.19±2.75	5.43E-08
Bacteroidota	Bacteroidia	Bacteroidales	Bacteroidaceae	Prevotella	copri	0.00E+00 [0.00E+00-0.00E+00]	0.00E+00 [0.00E+00-0.00E+00]	16.01±4.52	6.72E-03
Proteobacteria	Gammaproteobacteria	Enterobacterales	Enterobacteriaceae	Citrobacter	portucalensis	1.01E-04 [2.18E-05-1.17E-04]	1.80E-04 [8.70E-05-3.93E-04]	9.22±1.50	7.52E-08
Proteobacteria	Gammaproteobacteria	Enterobacterales	Enterobacteriaceae	Citrobacter	unknown	4.86E-06 [2.13E-06-3.56E-05]	3.97E-05 [1.06E-05-2.12E-03]	8.64±1.48	3.92E-07
Proteobacteria	Gammaproteobacteria	Enterobacterales	Enterobacteriaceae	Klebsiella_A	michiganensis	4.05E-06 [2.59E-06-3.24E-05]	2.08E-05 [2.75E-06-1.84E-04]	8.09±1.33	1.04E-07
Proteobacteria	Gammaproteobacteria	Enterobacterales	Enterobacteriaceae	Yersinia	massiliensis	0.00E+00 [0.00E+00-0.00E+00]	4.82E-07 [0.00E+00-2.36E-06]	7.99±2.26	6.72E-03
Proteobacteria	Gammaproteobacteria	Enterobacterales	Enterobacteriaceae	Raoultella	planticola	2.67E-07 [0.00E+00-7.40E-07]	3.80E-06 [0.00E+00-1.32E-05]	7.78±1.68	2.39E-04
Proteobacteria	Gammaproteobacteria	Enterobacterales	Enterobacteriaceae	Erwinia	pyrifoliae	0.00E+00 [0.00E+00-0.00E+00]	0.00E+00 [0.00E+00-1.45E-06]	6.81±2.36	3.76E-02
Proteobacteria	Gammaproteobacteria	Enterobacterales	Enterobacteriaceae	Cronobacter	malonaticus	1.06E-06 [0.00E+00-4.07E-06]	6.21E-06 [0.00E+00-1.65E-05]	6.78±1.62	1.03E-03
Proteobacteria	Gammaproteobacteria	Enterobacterales	Enterobacteriaceae	Kluyvera	ascorbata	0.00E+00 [0.00E+00-4.05E-07]	2.43E-07 [0.00E+00-1.91E-06]	6.74±2.04	1.14E-02
Proteobacteria	Gammaproteobacteria	Enterobacterales	Enterobacteriaceae	Klebsiella_A	unknown	7.40E-07 [0.00E+00-5.84E-06]	4.28E-06 [3.48E-07-1.12E-04]	6.68±1.89	6.72E-03
Proteobacteria	Gammaproteobacteria	Enterobacterales	Enterobacteriaceae	Lelliottia	sp000016325	0.00E+00 [0.00E+00-0.00E+00]	9.73E-07 [0.00E+00-1.19E-06]	6.23±2.05	2.45E-02
Proteobacteria	Gammaproteobacteria	Enterobacterales	Enterobacteriaceae	Citrobacter	europaeus	5.70E-07 [0.00E+00-1.23E-05]	6.23E-06 [1.74E-07-6.34E-05]	6.21±1.78	7.46E-03
Proteobacteria	Gammaproteobacteria	Enterobacterales	Enterobacteriaceae	Mangrovibacter	phragmitis	0.00E+00 [0.00E+00-0.00E+00]	4.82E-07 [0.00E+00-1.04E-06]	6.18±2.19	4.23E-02
Firmicutes	Bacilli	Lactobacillales	Streptococcaceae	Streptococcus	anginosus	0.00E+00 [0.00E+00-1.59E-06]	9.65E-07 [3.89E-07-1.44E-05]	6.06±1.48	1.36E-03
Proteobacteria	Gammaproteobacteria	Enterobacterales	Enterobacteriaceae	Cronobacter	dublinensis	4.49E-07 [0.00E+00-3.31E-06]	4.85E-07 [0.00E+00-3.89E-06]	5.92±1.63	5.49E-03
Proteobacteria	Gammaproteobacteria	Enterobacterales	Enterobacteriaceae	Citrobacter	braakii	6.55E-05 [3.41E-05-1.06E-04]	1.56E-04 [9.58E-05-1.42E-03]	5.92±1.35	5.24E-04
Proteobacteria	Gammaproteobacteria	Enterobacterales	Enterobacteriaceae	Kosakonia	radicincitans	0.00E+00 [0.00E+00-5.52E-07]	0.00E+00 [0.00E+00-3.45E-07]	5.90±1.86	1.67E-02
Proteobacteria	Gammaproteobacteria	Enterobacterales	Enterobacteriaceae	Raoultella	unknown	0.00E+00 [0.00E+00-2.76E-07]	7.28E-07 [0.00E+00-2.76E-06]	5.80±1.69	8.80E-03
Proteobacteria	Gammaproteobacteria	Enterobacterales	Enterobacteriaceae	Raoultella	ornithinolytica	1.35E-06 [0.00E+00-8.55E-06]	1.94E-06 [0.00E+00-2.55E-05]	5.77±1.70	9.64E-03
Firmicutes	Bacilli	Lactobacillales	Streptococcaceae	Streptococcus	canis	4.05E-07 [0.00E+00-2.34E-06]	2.72E-06 [0.00E+00-3.08E-05]	5.75±1.79	1.50E-02
Proteobacteria	Gammaproteobacteria	Enterobacterales	Enterobacteriaceae	Enterobacter	cloacae_M	0.00E+00 [0.00E+00-8.09E-07]	4.87E-06 [0.00E+00-3.18E-05]	5.45±1.62	1.00E-02
Proteobacteria	Gammaproteobacteria	Enterobacterales	Enterobacteriaceae	Enterobacter	cloacae	0.00E+00 [0.00E+00-1.34E-06]	1.94E-06 [0.00E+00-9.05E-06]	5.08±1.51	1.00E-02
Firmicutes	Bacilli	Lactobacillales	Streptococcaceae	Streptococcus	henryi	0.00E+00 [0.00E+00-0.00E+00]	4.75E-07 [0.00E+00-3.31E-06]	5.04±1.58	1.60E-02
Proteobacteria	Gammaproteobacteria	Enterobacterales	Enterobacteriaceae	Enterobacter	unknown	1.08E-05 [2.48E-06-3.18E-05]	3.57E-05 [1.28E-05-1.09E-04]	4.71±1.42	1.12E-02

Proteobacteria	Gammaproteobacteria	Enterobacterales	Enterobacteriaceae	Enterobacter	kobei	2.14E-05 [0.00E+00-2.70E-05]	1.01E-05 [3.33E-06-1.16E-04]	4.64±1.40	1.12E-02
Proteobacteria	Gammaproteobacteria	Enterobacterales	Enterobacteriaceae	Hafnia	paralvei	1.08E-05 [7.97E-07-3.10E-05]	4.49E-05 [9.67E-06-7.19E-04]	4.41±1.40	1.79E-02
Firmicutes_A	Clostridia	Lachnospirales	Lachnospiraceae	Blautia	sp900541955	1.88E-04 [1.04E-04-2.85E-04]	2.76E-05 [1.25E-05-6.24E-05]	4.36±1.27	8.80E-03
Firmicutes	Bacilli	Lactobacillales	Lactobacillaceae	Lentilactobacillus	parabuchneri	8.55E-07 [4.05E-07-8.24E-06]	5.84E-07 [0.00E+00-3.49E-05]	4.21±1.22	8.56E-03
Proteobacteria	Gammaproteobacteria	Enterobacterales	Enterobacteriaceae	Enterobacter	roggenkampii	9.89E-06 [4.25E-06-1.38E-05]	8.73E-06 [9.73E-07-9.01E-05]	4.14±1.49	4.61E-02
Firmicutes	Bacilli	Lactobacillales	Streptococcaceae	Streptococcus	suis	3.46E-06 [1.85E-06-6.14E-06]	1.55E-06 [9.98E-07-2.72E-05]	4.01±0.97	1.23E-03
Firmicutes	Bacilli	Lactobacillales	Streptococcaceae	Streptococcus	thermophilus	1.17E-05 [6.08E-06-1.63E-05]	4.63E-05 [1.77E-05-1.51E-04]	3.91±1.00	2.30E-03
Firmicutes	Bacilli	Lactobacillales	Streptococcaceae	Streptococcus	macedonicus	8.54E-06 [3.72E-06-6.32E-05]	1.06E-04 [5.82E-06-7.23E-04]	3.91±1.31	2.85E-02
Firmicutes_A	Clostridia	Peptostreptococcales	Peptostreptococcaceae	Clostridioides	difficile	3.95E-05 [1.89E-05-5.70E-05]	5.90E-05 [2.85E-05-1.08E-04]	3.48±1.03	9.70E-03
Firmicutes	Bacilli	unknown	unknown	unknown	unknown	7.54E-05 [2.43E-05-2.03E-04]	3.12E-04 [5.25E-05-2.23E-03]	3.29±0.93	6.72E-03
Firmicutes	Bacilli	Lactobacillales	unknown	unknown	unknown	4.05E-04 [1.77E-04-1.85E-03]	1.41E-03 [2.43E-04-1.17E-02]	2.84±1.03	4.72E-02
Firmicutes_A	Clostridia	Peptostreptococcales	Anaerovoracaceae	Emergencia	timonensis	1.85E-06 [3.10E-07-3.24E-06]	1.38E-06 [3.22E-07-4.72E-06]	2.70±0.95	4.22E-02
Firmicutes_A	Clostridia	Lachnospirales	Lachnospiraceae	Anaerostipes	hadrus	2.26E-05 [7.69E-06-3.04E-05]	1.70E-05 [6.21E-06-6.14E-05]	2.48±0.75	1.14E-02
Actinobacteriota	Actinomycetia	Actinomycetales	Bifidobacteriaceae	Bifidobacterium	breve	3.50E-06 [1.60E-06-4.42E-06]	3.11E-06 [7.77E-07-1.18E-05]	2.39±0.86	4.61E-02
Firmicutes	Bacilli	Erysipelotrichales	Erysipelotrichaceae	Holdemanella	sp002299315	2.18E-05 [1.54E-05-4.13E-05]	2.59E-05 [6.99E-06-1.57E-04]	2.09±0.56	3.96E-03
**Higher in the first tertile at baseline (43 species)**									
Actinobacteriota	Coriobacteriia	Coriobacteriales	Eggerthellaceae	Slackia_A	sp900553775	0.00E+00 [0.00E+00-0.00E+00]	0.00E+00 [0.00E+00-0.00E+00]	-25.83±5.57	2.37E-04
Actinobacteriota	unknown	unknown	unknown	unknown	unknown	8.09E-07 [0.00E+00-7.48E-06]	0.00E+00 [0.00E+00-0.00E+00]	-20.53±2.05	1.05E-20
Actinobacteriota	Coriobacteriia	Coriobacteriales	Eggerthellaceae	Slackia_A	unknown	0.00E+00 [0.00E+00-0.00E+00]	0.00E+00 [0.00E+00-0.00E+00]	-20.49±5.16	2.00E-03
Actinobacteriota	Actinomycetia	Actinomycetales	Bifidobacteriaceae	Bifidobacterium	unknown	0.00E+00 [0.00E+00-2.67E-07]	0.00E+00 [0.00E+00-0.00E+00]	-18.11±2.66	3.20E-09
Actinobacteriota	Coriobacteriia	Coriobacteriales	Eggerthellaceae	Slackia_A	piriformis	0.00E+00 [0.00E+00-2.66E-07]	0.00E+00 [0.00E+00-0.00E+00]	-17.85±4.14	6.91E-04
Firmicutes	Bacilli	Lactobacillales	Lactobacillaceae	Pediococcus	pentosaceus	0.00E+00 [0.00E+00-0.00E+00]	0.00E+00 [0.00E+00-0.00E+00]	-16.05±2.43	6.19E-09
Firmicutes	Bacilli	Lactobacillales	Lactobacillaceae	Leuconostoc	carnosum	0.00E+00 [0.00E+00-0.00E+00]	0.00E+00 [0.00E+00-0.00E+00]	-14.17±3.15	3.46E-04
Actinobacteriota	Actinomycetia	Mycobacteriales	Mycobacteriaceae	Corynebacterium	auriscanis	0.00E+00 [0.00E+00-1.21E-06]	0.00E+00 [0.00E+00-0.00E+00]	-10.80±3.22	1.01E-02
Firmicutes	Erysipelotrichia	Erysipelotrichales	Erysipelotrichaceae	Catenibacterium	mitsuokai	0.00E+00 [0.00E+00-3.70E-07]	0.00E+00 [0.00E+00-0.00E+00]	-9.80±2.72	5.83E-03
Actinobacteriota	Actinomycetia	Actinomycetales	Bifidobacteriaceae	Bifidobacterium	infantis	0.00E+00 [0.00E+00-7.40E-07]	0.00E+00 [0.00E+00-0.00E+00]	-8.22±2.39	8.56E-03
Bacteroidota	Bacteroidia	Bacteroidales	Bacteroidaceae	Bacteroides	xylanisolvens	1.87E-05 [4.25E-06-5.18E-05]	0.00E+00 [0.00E+00-1.70E-06]	-7.63±1.85	1.23E-03
Firmicutes	Bacilli	Haloplasmatales	Turicibacteraceae	Turicibacter	sanguinis	1.62E-05 [0.00E+00-3.81E-04]	0.00E+00 [0.00E+00-3.45E-07]	-7.04±1.99	6.72E-03
Firmicutes_A	Clostridia	Peptostreptococcales	Peptostreptococcaceae	Terrisporobacter	unknown	8.77E-06 [3.10E-07-1.17E-05]	0.00E+00 [0.00E+00-1.61E-06]	-6.24±1.48	1.03E-03
Firmicutes	Bacilli	Lactobacillales	Enterococcaceae	Enterococcus_C	dispar	1.06E-06 [0.00E+00-1.87E-05]	0.00E+00 [0.00E+00-7.87E-07]	-6.19±1.58	2.28E-03
Firmicutes_A	Clostridia	Clostridiales	Clostridiaceae	Clostridium	sp000753455	4.04E-06 [1.55E-06-1.79E-05]	5.22E-07 [0.00E+00-1.29E-06]	-6.14±1.71	6.25E-03
Bacteroidota	Bacteroidia	Bacteroidales	Bacteroidaceae	Bacteroides	caecimuris	0.00E+00 [0.00E+00-1.98E-05]	0.00E+00 [0.00E+00-2.38E-07]	-5.93±2.16	4.94E-02
Firmicutes_A	Clostridia	Peptostreptococcales	Peptostreptococcaceae	Terrisporobacter	glycolicus_A	8.24E-06 [2.67E-07-1.98E-05]	7.28E-07 [0.00E+00-1.73E-06]	-5.90±1.45	1.53E-03
Bacteroidota	Bacteroidia	Bacteroidales	Tannerellaceae	Parabacteroides	gordonii	0.00E+00 [0.00E+00-9.62E-06]	0.00E+00 [0.00E+00-0.00E+00]	-5.85±2.06	4.07E-02
Firmicutes_A	Clostridia	Peptostreptococcales	Peptostreptococcaceae	Terrisporobacter	glycolicus	9.68E-06 [0.00E+00-2.56E-05]	1.55E-06 [0.00E+00-3.86E-06]	-5.77±1.50	2.56E-03
Firmicutes_A	Clostridia	Lachnospirales	Lachnospiraceae	Blautia_A	sp000433815	5.68E-05 [8.01E-07-4.81E-04]	1.29E-06 [2.38E-07-1.74E-06]	-5.54±1.52	5.42E-03

Bacteroidota	Bacteroidia	Bacteroidales	Bacteroidaceae	Phocaeicola	sp900546645	8.28E-07 [0.00E+00-9.03E-05]	7.79E-07 [0.00E+00-6.55E-06]	-5.35±1.67	1.55E-02
Firmicutes_A	Clostridia	Lachnospirales	Lachnospiraceae	Blautia_A	sp900541345	1.23E-04 [1.57E-05-4.54E-04]	7.88E-06 [9.70E-07-1.27E-05]	-5.17±1.30	2.04E-03
Firmicutes_A	Clostridia	Lachnospirales	Lachnospiraceae	Anaerobutyricum	hallii_A	1.17E-06 [2.85E-07-3.24E-06]	0.00E+00 [0.00E+00-0.00E+00]	-5.08±1.32	2.57E-03
Firmicutes_A	Clostridia	Clostridiales	Clostridiaceae	Clostridium	chauvoei	7.40E-07 [0.00E+00-1.59E-06]	0.00E+00 [0.00E+00-0.00E+00]	-4.98±1.72	3.73E-02
Firmicutes_A	Clostridia	Oscillospirales	Ruminococcaceae	Faecalibacterium	prausnitzii_C	2.25E-06 [9.31E-07-9.71E-06]	0.00E+00 [0.00E+00-2.43E-07]	-4.95±1.08	2.39E-04
Firmicutes_A	Clostridia	Lachnospirales	Lachnospiraceae	Ruminococcus_A	sp000432335	2.17E-05 [1.39E-05-3.43E-04]	4.99E-07 [0.00E+00-1.94E-06]	-4.82±1.24	2.37E-03
Bacteroidota	Bacteroidia	Bacteroidales	Bacteroidaceae	Bacteroides	ovatus	4.67E-05 [4.69E-06-6.99E-04]	4.82E-07 [0.00E+00-5.83E-05]	-4.70±1.67	4.23E-02
Firmicutes_A	Clostridia	Lachnospirales	Lachnospiraceae	Blautia_A	sp900120195	2.92E-06 [1.14E-06-6.52E-06]	0.00E+00 [0.00E+00-0.00E+00]	-4.67±1.15	1.54E-03
Firmicutes_A	Clostridia	Lachnospirales	Lachnospiraceae	Schaedlerella	glycyrrhizinilyticum	7.91E-05 [2.70E-05-1.39E-04]	4.49E-06 [9.51E-07-6.62E-06]	-4.59±1.01	2.71E-04
Firmicutes_A	Clostridia	Lachnospirales	Lachnospiraceae	Coprococcus	eutactus	3.10E-07 [0.00E+00-2.34E-06]	0.00E+00 [0.00E+00-0.00E+00]	-4.59±1.65	4.52E-02
Firmicutes_A	Clostridia	Clostridiales	Clostridiaceae	Clostridium	celatum	5.32E-06 [1.34E-06-5.89E-06]	0.00E+00 [0.00E+00-2.34E-06]	-4.53±1.58	3.82E-02
Firmicutes_A	Clostridia	Peptostreptococcales	Peptostreptococcaceae	Asaccharospora	irregularis	3.50E-06 [2.67E-07-4.94E-06]	0.00E+00 [0.00E+00-0.00E+00]	-4.51±1.44	1.88E-02
Actinobacteriota	Actinomycetia	unknown	unknown	unknown	unknown	2.34E-06 [5.70E-07-1.53E-05]	5.84E-07 [2.49E-07-1.61E-06]	-4.12±1.06	2.48E-03
Firmicutes_A	Clostridia	Lachnospirales	Lachnospiraceae	Dorea	sp900240315	2.30E-05 [8.99E-06-5.86E-05]	1.93E-06 [0.00E+00-2.76E-06]	-4.06±1.04	2.30E-03
Actinobacteriota	Actinomycetia	Actinomycetales	unknown	unknown	unknown	4.44E-06 [1.17E-06-1.21E-05]	1.13E-06 [2.49E-07-1.19E-06]	-3.85±1.11	7.83E-03
Firmicutes_A	Clostridia	Clostridiales	Clostridiaceae	Clostridium	unknown	9.31E-06 [7.69E-06-4.07E-05]	2.85E-06 [3.89E-07-4.67E-06]	-3.82±1.22	1.88E-02
Firmicutes_A	Clostridia	Lachnospirales	Lachnospiraceae	Anaerostipes	caccae	3.67E-04 [1.90E-04-6.80E-03]	3.50E-06 [7.48E-07-3.97E-05]	-3.81±1.38	4.68E-02
Firmicutes_A	Clostridia	Peptostreptococcales	Peptostreptococcaceae	Paeniclostridium	sordellii	1.71E-06 [5.34E-07-1.54E-05]	2.49E-07 [0.00E+00-3.94E-07]	-3.60±1.24	3.66E-02
Firmicutes_A	Clostridia	Lachnospirales	Cellulosilyticaceae	Cellulosilyticum	unknown	2.25E-05 [7.14E-06-6.19E-05]	1.18E-06 [2.43E-07-3.80E-06]	-3.31±1.18	4.37E-02
Firmicutes_A	Clostridia	Lachnospirales	Lachnospiraceae	Blautia_A	sp900540785	2.22E-04 [1.34E-05-1.19E-03]	1.13E-05 [1.70E-06-3.41E-05]	-3.20±1.05	2.30E-02
Firmicutes_A	Clostridia	Lachnospirales	Lachnospiraceae	Faecalimonas	sp900550235	2.03E-04 [7.10E-05-2.98E-04]	8.16E-06 [3.94E-06-1.32E-05]	-3.01±0.89	9.64E-03
Firmicutes_A	Clostridia	Lachnospirales	Lachnospiraceae	Anaerostipes	unknown	1.57E-05 [8.07E-06-4.15E-05]	9.65E-07 [2.43E-07-2.33E-06]	-2.66±0.95	4.52E-02
Firmicutes_A	Clostridia	Lachnospirales	Lachnospiraceae	Ruminococcus_B	gnavus	4.80E-02 [2.56E-02-7.39E-02]	1.43E-03 [2.62E-04-2.51E-03]	-2.44±0.84	3.56E-02

FC: fold change, SE: standard error

[1] p values were adjusted with false discovery rate for multiple comparisons

Supplemental Table S10. Species with differential abundances between baseline and week 10 in low responders in PBO (n=8, $\log_2$|fold change| ≥ 2 and FDR-adjusted p < 0.05)

Phylum	Class	Order	Family	Genus	Species	Relative abundance (%) Median (IQR)		Week 10 vs Baseline	
						Baseline	Week 10	Log 2 FC mean ± SE	Adjusted *P*-value[1]
**Increased at week 10 (21 species)**									
Firmicutes_A	Clostridia	Oscillospirales	Oscillospiraceae	Flavonifractor	unknown	0.00E+00 [0.00E+00-0.00E+00]	7.37E-07 [0.00E+00-1.36E-04]	11.61±3.70	3.70E-02
Firmicutes	Bacilli	Lactobacillales	Lactobacillaceae	Leuconostoc	mesenteroides	0.00E+00 [0.00E+00-0.00E+00]	2.34E-07 [0.00E+00-2.34E-06]	10.84±2.39	5.84E-04
Proteobacteria	Gammaproteobacteria	Enterobacterales	Enterobacteriaceae	Citrobacter	europaeus	9.87E-07 [0.00E+00-7.65E-06]	4.77E-05 [2.95E-05-8.01E-04]	8.13±1.58	1.04E-04
Proteobacteria	Gammaproteobacteria	Enterobacterales	Enterobacteriaceae	Citrobacter	youngae	7.60E-07 [0.00E+00-2.59E-06]	1.24E-05 [1.57E-06-1.05E-04]	6.91±1.60	1.34E-03
Bacteroidota	Bacteroidia	Bacteroidales	Bacteroidaceae	Bacteroides	fragilis	1.11E-05 [4.50E-07-5.70E-02]	4.37E-03 [4.18E-04-1.07E-02]	6.20±1.32	3.66E-04
Firmicutes	Bacilli	Lactobacillales	Streptococcaceae	Lactococcus	lactis	4.54E-06 [0.00E+00-6.74E-06]	4.13E-06 [2.79E-06-1.34E-03]	5.83±1.18	1.57E-04
Firmicutes	Bacilli	Lactobacillales	Enterococcaceae	Enterococcus_C	dispar	0.00E+00 [0.00E+00-3.36E-07]	2.96E-06 [1.75E-07-5.05E-06]	5.81±1.88	3.70E-02
Firmicutes_C	Negativicutes	Selenomonadales	Selenomonadaceae	Megamonas	funiformis	1.09E-07 [0.00E+00-2.54E-03]	1.05E-04 [4.20E-06-1.19E-02]	5.68±1.47	5.92E-03
Firmicutes_A	Clostridia	Oscillospirales	Oscillospiraceae	Flavonifractor	sp000508885	0.00E+00 [0.00E+00-3.92E-06]	4.17E-06 [7.84E-07-1.25E-05]	5.37±1.62	2.46E-02
Proteobacteria	Gammaproteobacteria	Enterobacterales	Enterobacteriaceae	Klebsiella	quasipneumoniae	9.39E-06 [8.40E-07-2.51E-05]	4.17E-05 [1.42E-05-1.09E-03]	5.09±1.26	3.63E-03
Proteobacteria	Gammaproteobacteria	Enterobacterales	Enterobacteriaceae	Enterobacter	ludwigii	1.80E-06 [4.50E-07-3.58E-06]	2.39E-05 [8.42E-06-5.04E-05]	4.78±1.53	3.70E-02
Proteobacteria	Gammaproteobacteria	Enterobacterales	Enterobacteriaceae	Klebsiella_A	oxytoca	1.94E-06 [2.74E-07-7.50E-06]	4.04E-05 [1.44E-05-9.05E-04]	4.77±1.54	3.70E-02
Proteobacteria	Gammaproteobacteria	Enterobacterales	Enterobacteriaceae	Raoultella	ornithinolytica	1.26E-06 [9.88E-07-3.46E-06]	9.12E-05 [1.31E-05-2.31E-03]	4.74±1.38	1.98E-02
Proteobacteria	Gammaproteobacteria	Enterobacterales	Enterobacteriaceae	Enterobacter	cloacae_M	1.01E-06 [1.47E-07-2.38E-06]	1.76E-05 [3.61E-06-1.05E-04]	4.68±1.41	2.46E-02
Firmicutes	Bacilli	Lactobacillales	Enterococcaceae	Enterococcus_D	sp002850555	7.15E-06 [1.47E-06-7.10E-05]	5.03E-04 [3.06E-05-1.40E-03]	4.46±1.45	3.71E-02
Proteobacteria	Gammaproteobacteria	Enterobacterales	Enterobacteriaceae	Enterobacter	unknown	6.74E-06 [3.29E-06-1.11E-05]	1.05E-04 [1.43E-05-3.11E-04]	4.37±1.10	4.15E-03
Proteobacteria	Gammaproteobacteria	Enterobacterales	Enterobacteriaceae	Citrobacter	unknown	1.16E-05 [8.63E-06-2.38E-05]	9.56E-04 [4.14E-04-1.88E-02]	4.32±1.29	2.42E-02
Proteobacteria	Gammaproteobacteria	Enterobacterales	Enterobacteriaceae	Enterobacter	kobei	8.94E-06 [1.46E-06-1.30E-05]	6.89E-05 [2.92E-05-2.11E-04]	4.31±1.15	7.82E-03
Firmicutes_A	Clostridia	Peptostreptococcales	Peptostreptococcaceae	Terrisporobacter	glycolicus	2.19E-06 [0.00E+00-1.94E-04]	7.92E-05 [2.25E-05-1.85E-04]	4.12±1.32	3.70E-02
Proteobacteria	Gammaproteobacteria	Enterobacterales	Enterobacteriaceae	Citrobacter	freundii	2.88E-05 [2.01E-05-3.83E-05]	6.42E-04 [1.23E-04-9.16E-03]	4.04±1.23	2.59E-02
Proteobacteria	Gammaproteobacteria	Enterobacterales	Enterobacteriaceae	Citrobacter	braakii	1.18E-04 [5.05E-05-1.78E-04]	6.92E-04 [2.05E-04-9.95E-03]	3.88±1.12	1.98E-02
**Decreased at week 10 (2 species)**									
Firmicutes	Bacilli	Lactobacillales	Enterococcaceae	Enterococcus_A	malodoratus	8.65E-06 [3.32E-06-7.13E-05]	1.09E-06 [0.00E+00-1.85E-05]	-5.10±1.43	1.35E-02
Firmicutes_A	Clostridia	Lachnospirales	Lachnospiraceae	Dorea	longicatena	2.01E-05 [7.57E-06-6.86E-05]	2.05E-06 [0.00E+00-7.52E-06]	-3.19±0.86	9.34E-03

FC: fold change, SE: standard error

[1] p values were adjusted with false discovery rate for multiple comparisons

Supplemental Table S11. Species with differential abundances between baseline and week 10 in high responders in PBO (n=8, $\log_2$|fold change| ≥ 2 and FDR-adjusted p < 0.05)

Phylum	Class	Order	Family	Genus	Species	Relative abundance (%) Median (IQR)		Week 10 vs Baseline	
						Baseline	Week 10	Log 2 FC mean ± SE	Adjusted *P*-value[1]
**Increased at week 10 (13 species)**									
Proteobacteria	Gammaproteobacteria	Enterobacterales	Enterobacteriaceae	Enterobacter	cancerogenus	0.00E+00 [0.00E+00-0.00E+00]	1.67E-07 [0.00E+00-8.48E-07]	22.35±3.38	1.75E-08
Proteobacteria	Gammaproteobacteria	Enterobacterales	Enterobacteriaceae	Lelliottia	amnigena_A	0.00E+00 [0.00E+00-0.00E+00]	0.00E+00 [0.00E+00-1.32E-06]	16.53±4.70	1.12E-02
Firmicutes_A	Clostridia	Oscillospirales	Oscillospiraceae	Flavonifractor	unknown	0.00E+00 [0.00E+00-0.00E+00]	6.20E-07 [0.00E+00-1.91E-04]	12.89±3.72	1.22E-02
Proteobacteria	Gammaproteobacteria	Enterobacterales	Enterobacteriaceae	Enterobacter	ludwigii	0.00E+00 [0.00E+00-3.81E-07]	0.00E+00 [0.00E+00-2.01E-05]	5.29±1.64	2.26E-02
Firmicutes_A	Clostridia	Lachnospirales	Lachnospiraceae	Anaerostipes	caccae	1.26E-05 [3.88E-06-3.52E-03]	1.24E-03 [1.65E-04-1.89E-03]	4.50±1.15	3.75E-03
Firmicutes_A	Clostridia	Lachnospirales	Lachnospiraceae	Ruminococcus_A	sp000432335	1.05E-05 [1.73E-07-2.85E-05]	1.65E-04 [3.93E-05-1.35E-03]	3.89±1.10	1.08E-02
Proteobacteria	Gammaproteobacteria	Enterobacterales	Enterobacteriaceae	Enterobacter	kobei	1.73E-07 [0.00E+00-3.48E-06]	1.27E-06 [4.76E-07-1.79E-05]	3.63±1.25	4.79E-02
Firmicutes	Bacilli	Erysipelotrichales	Erysipelotrichaceae	Longicatena	dolichum	1.11E-04 [3.73E-06-1.16E-03]	1.53E-03 [7.03E-04-3.85E-03]	3.45±1.19	4.93E-02
Firmicutes_A	Clostridia	Lachnospirales	Lachnospiraceae	Blautia	sp000432195	1.66E-04 [1.21E-05-2.23E-03]	2.24E-03 [5.35E-04-8.70E-03]	3.40±0.88	3.75E-03
Firmicutes_A	Clostridia	Lachnospirales	Lachnospiraceae	Faecalimonas	sp900551895	3.09E-04 [3.01E-05-2.03E-03]	3.64E-03 [1.73E-03-5.80E-03]	3.37±0.84	3.75E-03
Firmicutes_A	Clostridia	Lachnospirales	Lachnospiraceae	Blautia_A	sp900540785	2.50E-04 [4.41E-06-1.41E-03]	1.71E-03 [1.86E-04-1.96E-02]	3.00±0.76	3.75E-03
Firmicutes_A	Clostridia	Lachnospirales	Lachnospiraceae	Blautia_A	sp900541345	1.78E-04 [1.84E-06-5.78E-03]	8.94E-03 [2.92E-04-1.24E-02]	2.86±0.95	4.26E-02
Firmicutes_A	Clostridia	Lachnospirales	Lachnospiraceae	Schaedlerella	glycyrrhizinilyticum	2.52E-05 [1.21E-06-8.76E-04]	2.59E-04 [1.51E-04-6.18E-04]	2.79±0.85	1.95E-02
**Decreased at week 10 (20 species)**									
Actinobacteriota	Actinomycetia	Actinomycetales	Bifidobacteriaceae	Bifidobacterium	animalis	0.00E+00 [0.00E+00-2.67E-07]	0.00E+00 [0.00E+00-0.00E+00]	-30.00±5.16	1.33E-06
Bacteroidota	Bacteroidia	Bacteroidales	Bacteroidaceae	Prevotella	copri	0.00E+00 [0.00E+00-2.14E-06]	0.00E+00 [0.00E+00-0.00E+00]	-26.05±7.27	9.78E-03
Firmicutes	Bacilli	Lactobacillales	Lactobacillaceae	Latilactobacillus	unknown	2.81E-06 [0.00E+00-1.74E-04]	0.00E+00 [0.00E+00-2.05E-07]	-9.94±3.31	4.26E-02
Firmicutes	Bacilli	Lactobacillales	Lactobacillaceae	Latilactobacillus	sakei_A	1.04E-05 [9.14E-07-4.02E-04]	0.00E+00 [0.00E+00-3.64E-07]	-9.65±2.49	3.75E-03
Firmicutes	Bacilli	Lactobacillales	Lactobacillaceae	Leuconostoc	lactis	1.39E-05 [2.51E-06-5.02E-05]	2.51E-07 [0.00E+00-1.09E-06]	-7.87±2.18	9.56E-03
Firmicutes	Bacilli	Lactobacillales	Lactobacillaceae	Lacticaseibacillus	rhamnosus	1.45E-04 [4.75E-06-7.03E-04]	5.02E-07 [0.00E+00-3.32E-06]	-7.30±1.73	2.21E-03
Firmicutes	Bacilli	Lactobacillales	Lactobacillaceae	Leuconostoc	unknown	3.87E-06 [6.86E-07-7.19E-04]	8.12E-07 [0.00E+00-2.71E-06]	-6.65±1.96	1.47E-02
Firmicutes	Bacilli	Lactobacillales	Lactobacillaceae	Lacticaseibacillus	unknown	2.26E-06 [0.00E+00-4.07E-05]	3.80E-07 [0.00E+00-2.85E-06]	-6.48±1.94	1.73E-02
Firmicutes	Bacilli	Lactobacillales	Lactobacillaceae	Lactiplantibacillus	plantarum	1.29E-05 [2.63E-06-7.34E-05]	3.04E-07 [0.00E+00-1.01E-06]	-6.47±1.67	3.75E-03
Firmicutes	Bacilli	Lactobacillales	Lactobacillaceae	Leuconostoc	gelidum	1.36E-05 [6.39E-07-1.20E-03]	5.85E-07 [0.00E+00-8.61E-06]	-6.00±1.42	2.21E-03
Firmicutes	Bacilli	Lactobacillales	Lactobacillaceae	Lacticaseibacillus	paracasei	8.44E-06 [3.49E-06-8.44E-05]	4.89E-07 [2.44E-07-2.12E-05]	-5.59±1.45	3.75E-03
Firmicutes	Bacilli	Lactobacillales	Streptococcaceae	Lactococcus	lactis	3.77E-05 [2.18E-05-9.80E-04]	7.46E-06 [3.63E-06-1.32E-05]	-5.05±1.14	1.25E-03
Firmicutes	Bacilli	Lactobacillales	Enterococcaceae	Enterococcus_A	gilvus	5.69E-06 [1.50E-06-1.26E-05]	5.41E-07 [0.00E+00-5.17E-06]	-4.57±1.42	2.26E-02
Firmicutes	Bacilli	Lactobacillales	Enterococcaceae	Enterococcus_B	durans	2.86E-05 [4.39E-06-1.40E-04]	2.39E-06 [2.12E-07-8.24E-06]	-4.54±1.31	1.22E-02

Firmicutes	Bacilli	Lactobacillales	Enterococcaceae	Enterococcus_A	unknown	1.78E-05 [3.53E-06-1.55E-04]	2.27E-06 [0.00E+00-8.15E-06]	-4.53±1.16	3.75E-03
Firmicutes	Bacilli	Lactobacillales	Lactobacillaceae	Leuconostoc	citreum	3.70E-06 [2.20E-06-4.97E-05]	1.87E-06 [1.64E-07-1.26E-05]	-4.36±1.49	4.64E-02
Bacteroidota	Bacteroidia	Bacteroidales	Bacteroidaceae	Phocaeicola	sp900546645	3.35E-05 [1.24E-06-1.16E-03]	3.82E-05 [2.32E-07-9.16E-05]	-4.00±1.34	4.41E-02
Firmicutes	Bacilli	Lactobacillales	Enterococcaceae	Enterococcus_G	italicus	3.97E-06 [1.43E-06-1.01E-05]	8.12E-07 [0.00E+00-2.92E-06]	-3.66±1.24	4.51E-02
Firmicutes	Bacilli	Lactobacillales	Enterococcaceae	Enterococcus_C	canintestini	2.45E-05 [2.86E-06-2.93E-04]	7.36E-06 [2.70E-06-1.12E-05]	-3.27±1.11	4.64E-02
Firmicutes	Bacilli	Lactobacillales	Streptococcaceae	Streptococcus	thermophilus	7.99E-05 [5.06E-05-4.18E-04]	3.70E-05 [7.47E-06-2.04E-04]	-3.25±0.99	1.95E-02

FC: fold change, SE: standard error

[1] p values were adjusted with false discovery rate for multiple comparisons

Supplemental Table S12. Species with differential abundances between baseline and week 10 in high responders in PNB (n=9, $\log_2|\text{fold change}| \geq 2$ and FDR-adjusted $p < 0.05$)

Phylum	Class	Order	Family	Genus	Species	Relative abundance (%) Median (IQR)		Week 10 vs Baseline	
						Baseline	Week 10	Log 2 FC mean ± SE	Adjusted *P*-value[1]
**Increased at week 10 (5 species)**									
Firmicutes	Bacilli	Lactobacillales	Lactobacillaceae	Weissella	cibaria	3.38E-07 [8.63E-08-8.89E-05]	2.71E-07 [0.00E+00-4.28E-06]	11.30±2.37	2.53E-04
Firmicutes	Bacilli	Lactobacillales	Lactobacillaceae	Weissella	confusa	1.55E-06 [0.00E+00-7.14E-06]	0.00E+00 [0.00E+00-8.71E-07]	8.45±2.52	2.92E-02
Firmicutes	Bacilli	Lactobacillales	Lactobacillaceae	Lacticaseibacillus	rhamnosus	1.45E-04 [4.75E-06-7.03E-04]	5.02E-07 [0.00E+00-3.32E-06]	6.19±1.73	1.79E-02
Proteobacteria	Gammaproteobacteria	Enterobacterales	Enterobacteriaceae	Citrobacter_A	amalonaticus_C	0.00E+00 [0.00E+00-2.88E-08]	0.00E+00 [0.00E+00-2.67E-07]	5.98±1.74	2.59E-02
Firmicutes_A	Clostridia	Peptostreptococcales	Peptostreptococcaceae	Clostridium_U	sp900539645	8.50E-04 [2.29E-06-5.30E-03]	3.91E-03 [2.05E-05-9.61E-03]	2.40±0.71	2.92E-02
**Decreased at week 10 (14 species)**									
Firmicutes	Bacilli	Lactobacillales	Streptococcaceae	Streptococcus	orisratti	6.87E-07 [3.85E-07-2.31E-06]	7.35E-07 [2.33E-07-5.69E-06]	-18.43±2.15	3.40E-15
Firmicutes	Bacilli	Lactobacillales	Streptococcaceae	Streptococcus	equinus	8.30E-05 [0.00E+00-7.09E-04]	7.30E-06 [1.64E-07-2.77E-04]	-13.49±1.54	1.30E-15
Firmicutes	Bacilli	Lactobacillales	Streptococcaceae	Streptococcus	gallolyticus	5.62E-05 [3.03E-07-2.65E-04]	3.48E-06 [3.69E-07-1.29E-04]	-9.97±1.35	3.60E-11
Firmicutes	Bacilli	Lactobacillales	Streptococcaceae	Streptococcus	equi	0.00E+00 [0.00E+00-5.05E-07]	2.71E-07 [0.00E+00-1.27E-06]	-7.64±2.00	8.34E-03
Firmicutes	Bacilli	Lactobacillales	Enterococcaceae	Enterococcus_A	avium	1.18E-03 [5.18E-07-1.14E-02]	8.32E-05 [9.52E-07-5.80E-04]	-7.34±1.32	4.47E-06
Firmicutes	Bacilli	Lactobacillales	Streptococcaceae	Streptococcus	equinus_B	3.87E-05 [3.03E-07-1.81E-04]	1.01E-06 [2.32E-07-1.64E-04]	-7.14±1.53	3.43E-04
Firmicutes	Bacilli	Lactobacillales	Enterococcaceae	Enterococcus_C	dispar	5.14E-07 [0.00E+00-1.96E-06]	0.00E+00 [0.00E+00-1.17E-06]	-6.31±1.77	1.79E-02
Firmicutes	Bacilli	Lactobacillales	Lactobacillaceae	Lacticaseibacillus	paracasei	8.44E-06 [3.49E-06-8.44E-05]	4.89E-07 [2.44E-07-2.12E-05]	-5.46±1.43	8.34E-03
Firmicutes	Bacilli	Lactobacillales	Enterococcaceae	Enterococcus_D	unknown	4.02E-06 [6.70E-07-4.46E-05]	3.07E-06 [0.00E+00-1.05E-05]	-5.23±1.32	5.89E-03
Firmicutes	Bacilli	Lactobacillales	Enterococcaceae	Enterococcus_D	casseliflavus	1.24E-05 [5.03E-06-4.41E-05]	2.36E-06 [5.04E-07-1.47E-05]	-5.21±1.26	3.26E-03
Firmicutes	Bacilli	Lactobacillales	Streptococcaceae	Streptococcus	infantarius	8.17E-05 [5.99E-06-7.10E-04]	2.29E-05 [4.04E-06-3.52E-04]	-5.05±1.21	2.84E-03
Firmicutes	Bacilli	Lactobacillales	Streptococcaceae	Streptococcus	unknown	3.67E-03 [2.14E-05-2.51E-02]	3.24E-03 [2.13E-05-1.11E-02]	-3.71±0.98	8.68E-03
Firmicutes	Bacilli	Lactobacillales	Enterococcaceae	Enterococcus_A	unknown	1.78E-05 [3.53E-06-1.55E-04]	2.27E-06 [0.00E+00-8.15E-06]	-3.62±1.08	2.92E-02
Firmicutes	Bacilli	Lactobacillales	Enterococcaceae	unknown	unknown	7.89E-04 [3.74E-04-1.44E-03]	1.07E-04 [3.03E-05-9.46E-04]	-3.05±0.90	2.92E-02

FC: fold change, SE: standard error

[1] p values were adjusted with false discovery rate for multiple comparisons

Supplemental Table S13. Species with differential abundances between baseline and week 10 in low responders in PNB (n=9, $\log_2$|fold change| ≥ 2 and FDR-adjusted p < 0.05)

Phylum	Class	Order	Family	Genus	Species	Relative abundance (%) Median (IQR)		Week 10 vs Baseline	
						Baseline	Week 10	Log 2 FC mean ± SE	Adjusted *P*-value[1]
**Increased at week 10 (40 species)**									
Bacteroidota	Bacteroidia	unknown	unknown	unknown	unknown	0.00E+00 [0.00E+00-0.00E+00]	0.00E+00 [0.00E+00-0.00E+00]	21.67±6.86	2.20E-02
Firmicutes_A	Clostridia	Lachnospirales	Cellulosilyticaceae	Cellulosilyticum	lentocellum	0.00E+00 [0.00E+00-0.00E+00]	0.00E+00 [0.00E+00-1.76E-06]	21.57±6.85	2.20E-02
Actinobacteriota	Coriobacteriia	Coriobacteriales	Eggerthellaceae	Slackia_A	unknown	0.00E+00 [0.00E+00-0.00E+00]	0.00E+00 [0.00E+00-0.00E+00]	21.40±6.86	2.26E-02
Actinobacteriota	Actinomycetia	Actinomycetales	Bifidobacteriaceae	Bifidobacterium	animalis	0.00E+00 [0.00E+00-0.00E+00]	0.00E+00 [0.00E+00-3.26E-03]	14.07±4.85	3.42E-02
Actinobacteriota	Actinomycetia	Actinomycetales	Bifidobacteriaceae	Bifidobacterium	unknown	0.00E+00 [0.00E+00-0.00E+00]	0.00E+00 [0.00E+00-1.06E-04]	13.78±4.22	1.90E-02
Firmicutes	Bacilli	Lactobacillales	Lactobacillaceae	Lacticaseibacillus	casei	0.00E+00 [0.00E+00-0.00E+00]	8.09E-07 [5.16E-07-6.58E-04]	11.42±2.74	2.42E-03
Firmicutes	Bacilli	Lactobacillales	Lactobacillaceae	Lacticaseibacillus	rhamnosus	2.43E-07 [0.00E+00-9.98E-07]	4.38E-04 [6.00E-07-7.25E-03]	9.35±1.67	5.42E-06
Firmicutes_A	Clostridia	Oscillospirales	Ruminococcaceae	Ruthenibacterium	lactatiformans	0.00E+00 [0.00E+00-1.95E-07]	3.76E-06 [4.06E-07-6.62E-06]	7.55±1.61	4.50E-04
Firmicutes	Bacilli	Lactobacillales	Lactobacillaceae	Lactobacillus	acidophilus	4.49E-07 [0.00E+00-2.51E-06]	5.05E-06 [1.18E-06-1.29E-03]	7.36±1.22	7.06E-07
Firmicutes	Bacilli	Lactobacillales	Enterococcaceae	Enterococcus_D	sp002140915	0.00E+00 [0.00E+00-0.00E+00]	3.42E-07 [0.00E+00-1.03E-06]	7.04±2.50	4.00E-02
Firmicutes	Bacilli	Lactobacillales	Lactobacillaceae	Lactobacillus	unknown	0.00E+00 [0.00E+00-0.00E+00]	6.82E-07 [0.00E+00-1.82E-05]	6.87±2.40	3.75E-02
Bacteroidota	Bacteroidia	Bacteroidales	Bacteroidaceae	Phocaeicola	sartorii	0.00E+00 [0.00E+00-0.00E+00]	2.36E-07 [0.00E+00-1.64E-05]	6.51±1.66	3.40E-03
Firmicutes	Bacilli	Lactobacillales	Streptococcaceae	Streptococcus	equinus	4.94E-06 [0.00E+00-4.96E-04]	2.57E-04 [5.98E-05-2.50E-03]	5.42±1.39	3.40E-03
Firmicutes_A	Clostridia	Lachnospirales	Lachnospiraceae	Eisenbergiella	sp900539715	0.00E+00 [0.00E+00-3.89E-07]	2.40E-06 [1.36E-06-8.28E-06]	5.28±1.18	9.70E-04
Firmicutes	Bacilli	Lactobacillales	Lactobacillaceae	Limosilactobacillus	fermentum	0.00E+00 [0.00E+00-0.00E+00]	2.40E-06 [1.03E-06-5.47E-06]	5.19±1.71	2.55E-02
Bacteroidota	Bacteroidia	Bacteroidales	Bacteroidaceae	Bacteroides	eggerthii	0.00E+00 [0.00E+00-1.17E-06]	1.36E-06 [0.00E+00-7.08E-05]	4.83±1.56	2.35E-02
Bacteroidota	Bacteroidia	Bacteroidales	Bacteroidaceae	Bacteroides	faecis	0.00E+00 [0.00E+00-3.50E-06]	1.55E-06 [0.00E+00-9.50E-05]	4.70±1.34	9.11E-03
Firmicutes	Bacilli	Erysipelotrichales	Erysipelotrichaceae	Longicatena	dolichum	4.05E-07 [0.00E+00-5.55E-06]	6.07E-05 [6.06E-07-2.89E-04]	4.65±1.17	3.36E-03
Firmicutes	Bacilli	Lactobacillales	Streptococcaceae	Streptococcus	equinus_B	4.49E-07 [0.00E+00-8.27E-05]	1.95E-04 [9.08E-06-1.48E-03]	4.45±1.40	2.10E-02
Firmicutes_A	Clostridia	Oscillospirales	Oscillospiraceae	Flavonifractor	plautii	4.05E-07 [0.00E+00-3.11E-06]	1.14E-05 [4.71E-06-7.51E-05]	4.30±1.20	8.10E-03
Firmicutes_A	Clostridia	Lachnospirales	Lachnospiraceae	Blautia_A	sp000433815	1.46E-06 [2.38E-07-1.65E-05]	4.59E-05 [1.11E-05-1.42E-04]	4.27±1.08	3.39E-03
Firmicutes_A	Clostridia	Lachnospirales	Lachnospiraceae	Clostridium_Q	sp000435655	4.05E-07 [0.00E+00-9.73E-06]	3.90E-05 [2.79E-05-1.66E-04]	3.87±0.91	2.11E-03
Firmicutes	Bacilli	Lactobacillales	Streptococcaceae	Streptococcus	lutetiensis	7.51E-05 [1.78E-05-8.56E-02]	7.58E-02 [3.06E-02-2.26E-01]	3.75±1.00	6.14E-03
Firmicutes_A	Clostridia	Lachnospirales	Lachnospiraceae	Ruminococcus_A	sp000432335	1.75E-06 [2.38E-07-1.39E-05]	2.41E-05 [2.16E-05-1.92E-04]	3.71±1.03	7.81E-03
Firmicutes	Bacilli	Lactobacillales	Streptococcaceae	Streptococcus	infantarius	5.92E-06 [8.09E-07-2.33E-04]	3.56E-04 [3.51E-05-1.07E-03]	3.65±1.14	2.10E-02
Firmicutes_A	Clostridia	Lachnospirales	Lachnospiraceae	Blautia_A	sp900541345	8.79E-06 [9.70E-07-1.27E-05]	1.35E-04 [4.90E-05-6.86E-04]	3.61±0.90	3.36E-03
Firmicutes	Bacilli	Lactobacillales	Streptococcaceae	Streptococcus	gallolyticus	1.16E-04 [0.00E+00-1.02E-03]	9.07E-04 [7.82E-05-2.36E-03]	3.48±1.18	3.10E-02
Firmicutes	Bacilli	Lactobacillales	Streptococcaceae	Streptococcus	unknown	7.66E-04 [4.86E-06-2.88E-02]	2.77E-02 [1.23E-02-8.61E-02]	3.40±0.97	9.11E-03

Firmicutes_A	Clostridia	Lachnospirales	Lachnospiraceae	Coprococcus	eutactus_A	2.38E-07 [0.00E+00-1.25E-06]	4.71E-06 [4.04E-06-1.16E-05]	3.33±0.89	6.23E-03
Firmicutes_A	Clostridia	Lachnospirales	Lachnospiraceae	Blautia	sp000432195	7.41E-05 [5.22E-06-3.35E-04]	4.82E-04 [1.97E-04-1.35E-02]	3.28±0.83	3.36E-03
Firmicutes_A	Clostridia	Lachnospirales	Lachnospiraceae	Schaedlerella	glycyrrhizinilyticum	4.49E-06 [1.94E-06-6.62E-06]	1.26E-04 [6.20E-05-1.94E-04]	3.23±0.80	3.36E-03
Firmicutes_A	Clostridia	Lachnospirales	Lachnospiraceae	Blautia	coccoides	3.70E-07 [0.00E+00-2.51E-06]	9.46E-06 [2.42E-06-3.46E-05]	2.96±0.93	2.10E-02
Firmicutes_A	Clostridia	Lachnospirales	Lachnospiraceae	Blautia	unknown	3.28E-05 [2.24E-05-8.02E-04]	1.44E-03 [4.59E-04-1.93E-03]	2.85±0.78	7.59E-03
Firmicutes_A	Clostridia	Lachnospirales	Cellulosilyticaceae	Cellulosilyticum	unknown	3.11E-06 [2.43E-07-6.18E-05]	2.50E-05 [1.77E-05-1.77E-04]	2.75±0.95	3.42E-02
Firmicutes_A	Clostridia	Lachnospirales	Lachnospiraceae	Eisenbergiella	tayi	1.95E-07 [0.00E+00-1.11E-06]	4.45E-06 [1.50E-06-2.10E-05]	2.61±0.94	4.44E-02
Firmicutes_A	Clostridia	Lachnospirales	Lachnospiraceae	Blautia_A	unknown	1.73E-05 [1.60E-05-8.00E-05]	3.44E-04 [1.89E-04-1.28E-03]	2.47±0.80	2.35E-02
Firmicutes	Bacilli	Erysipelotrichales	Erysipelotrichaceae	Holdemanella	biformis	2.43E-07 [0.00E+00-1.62E-06]	1.11E-05 [2.13E-06-4.13E-05]	2.46±0.85	3.42E-02
Firmicutes_A	Clostridia	Lachnospirales	Lachnospiraceae	Roseburia	intestinalis	2.38E-07 [0.00E+00-2.70E-06]	1.07E-05 [4.26E-06-2.00E-05]	2.42±0.71	1.14E-02
Firmicutes_A	Clostridia	Lachnospirales	Lachnospiraceae	Fusicatenibacter	saccharivorans	1.21E-06 [0.00E+00-4.07E-06]	1.04E-05 [6.00E-06-4.45E-05]	2.18±0.65	1.56E-02
Firmicutes_A	Clostridia	Peptostreptococcales	Peptostreptococcaceae	Clostridium_U	sp900539645	5.39E-06 [3.89E-07-3.60E-04]	4.98E-05 [6.06E-06-5.13E-03]	2.12±0.74	3.78E-02
**Decreased at week 10 (20 species)**									
Bacteroidota	Bacteroidia	Bacteroidales	Bacteroidaceae	Prevotella	sp900290275	0.00E+00 [0.00E+00-0.00E+00]	0.00E+00 [0.00E+00-0.00E+00]	-24.50±6.86	8.10E-03
Bacteroidota	Bacteroidia	Bacteroidales	Marinifilaceae	Gabonibacter	massiliensis	0.00E+00 [0.00E+00-0.00E+00]	0.00E+00 [0.00E+00-0.00E+00]	-21.84±6.87	2.10E-02
Firmicutes	Bacilli	Lactobacillales	Enterococcaceae	Enterococcus	faecalis	1.80E-03 [4.12E-04-1.36E-02]	4.36E-04 [8.25E-06-8.20E-04]	-4.35±1.22	8.10E-03
Firmicutes_C	Negativicutes	Selenomonadales	Selenomonadaceae	Megamonas	funiformis	3.24E-06 [3.48E-07-2.23E-05]	0.00E+00 [0.00E+00-7.32E-05]	-4.25±1.40	2.55E-02
Proteobacteria	Gammaproteobacteria	Enterobacterales	Enterobacteriaceae	Citrobacter	unknown	7.46E-05 [4.86E-06-2.12E-03]	1.39E-05 [1.03E-06-9.08E-05]	-3.69±1.22	2.63E-02
Proteobacteria	unknown	unknown	unknown	unknown	unknown	7.77E-06 [4.87E-06-1.21E-05]	7.50E-06 [0.00E+00-1.33E-05]	-3.61±0.98	6.54E-03
Proteobacteria	Gammaproteobacteria	Enterobacterales	Enterobacteriaceae	Yersinia	pestis	4.92E-05 [7.84E-06-1.45E-04]	2.16E-05 [0.00E+00-1.14E-04]	-3.59±1.19	2.60E-02
Proteobacteria	Gammaproteobacteria	Enterobacterales	Enterobacteriaceae	Cronobacter	sakazakii	4.17E-05 [4.07E-06-5.00E-05]	4.84E-06 [4.50E-07-1.91E-05]	-3.58±1.14	2.23E-02
Proteobacteria	Gammaproteobacteria	Enterobacterales	Enterobacteriaceae	Klebsiella_A	grimontii	2.33E-05 [6.10E-06-1.26E-04]	3.63E-06 [1.13E-06-1.32E-05]	-3.57±1.19	2.80E-02
Proteobacteria	Gammaproteobacteria	unknown	unknown	unknown	unknown	7.68E-05 [3.78E-05-2.59E-04]	4.15E-05 [3.07E-05-1.07E-04]	-3.46±0.99	9.11E-03
Firmicutes_A	Clostridia	Clostridiales	Clostridiaceae	Clostridium	saudiense	6.74E-06 [5.22E-07-1.36E-05]	3.42E-07 [1.50E-07-3.07E-06]	-3.45±1.12	2.41E-02
Firmicutes	Bacilli	Haloplasmatales	Turicibacteraceae	Turicibacter	sp001543345	3.89E-06 [1.26E-06-4.74E-05]	2.57E-06 [4.04E-07-1.57E-05]	-3.39±1.09	2.26E-02
Proteobacteria	Gammaproteobacteria	Enterobacterales	Enterobacteriaceae	Salmonella	enterica	1.87E-04 [1.59E-04-2.26E-04]	7.76E-05 [4.16E-05-1.90E-04]	-3.23±1.00	1.95E-02
Proteobacteria	Gammaproteobacteria	Enterobacterales	unknown	unknown	unknown	5.14E-05 [1.74E-05-1.03E-04]	1.15E-05 [3.61E-06-1.31E-04]	-3.06±1.09	4.00E-02
Proteobacteria	Gammaproteobacteria	Enterobacterales	Enterobacteriaceae	unknown	unknown	2.34E-02 [1.57E-02-3.22E-02]	1.29E-02 [9.47E-03-1.69E-02]	-2.97±1.00	2.84E-02
Firmicutes	Bacilli	Lactobacillales	Enterococcaceae	Enterococcus_B	faecium_B	5.70E-04 [5.26E-05-2.53E-03]	8.17E-04 [7.64E-05-2.34E-03]	-2.86±1.02	4.07E-02
Proteobacteria	Gammaproteobacteria	Enterobacterales	Enterobacteriaceae	Citrobacter_A	amalonaticus	3.18E-04 [9.19E-05-5.50E-04]	2.32E-04 [9.13E-05-4.69E-04]	-2.82±1.03	4.86E-02
Proteobacteria	Gammaproteobacteria	Enterobacterales	Enterobacteriaceae	Escherichia	albertii	1.79E-03 [1.40E-03-3.12E-03]	1.60E-03 [8.30E-04-2.99E-03]	-2.78±0.99	4.00E-02
Proteobacteria	Gammaproteobacteria	Burkholderiales	Burkholderiaceae	Burkholderia	cenocepacia	7.76E-06 [1.75E-06-1.37E-05]	4.74E-06 [1.69E-06-7.27E-06]	-2.73±0.96	3.78E-02
Actinobacteriota	Actinomycetia	Actinomycetales	Actinomycetaceae	Buchananella	hordeovulneris	1.78E-05 [5.23E-06-2.83E-05]	3.31E-06 [3.01E-06-1.33E-05]	-2.49±0.78	2.10E-02

FC: fold change, SE: standard error

[1] p values were adjusted with false discovery rate for multiple comparisons

Supplemental Table S14. Species with differential abundances between low responders (LR, n=9) and high responders (HR, n=9) in PNB at baseline ($\log_2$|fold change| ≥ 2 and FDR-adjusted p < 0.05)

Phylum	Class	Order	Family	Genus	Species	Relative abundance (%) Median (IQR)		HR vs LR	
						LR	HR	Log 2 FC mean ± SE	Adjusted *P*-value[1]
**Higher in LR at baseline (29 species)**									
Firmicutes	Bacilli	Erysipelotrichales	Erysipelotrichaceae	Bulleidia	moorei	0.00E+00 [0.00E+00-4.49E-07]	0.00E+00 [0.00E+00-0.00E+00]	-19.28±3.24	2.30E-07
Proteobacteria	Gammaproteobacteria	Enterobacterales	Enterobacteriaceae	Providencia	rustigianii	0.00E+00 [0.00E+00-0.00E+00]	0.00E+00 [0.00E+00-0.00E+00]	-15.69±2.82	1.66E-06
Proteobacteria	Gammaproteobacteria	Enterobacterales	Enterobacteriaceae	Citrobacter	unknown	7.46E-05 [4.86E-06-2.12E-03]	6.81E-06 [2.13E-06-1.85E-05]	-9.40±1.60	2.87E-07
Proteobacteria	Gammaproteobacteria	Enterobacterales	Enterobacteriaceae	Citrobacter	portucalensis	1.80E-04 [1.01E-04-3.93E-04]	6.24E-05 [2.18E-05-1.14E-04]	-9.12±1.45	3.99E-08
Proteobacteria	Gammaproteobacteria	Pseudomonadales	Moraxellaceae	Acinetobacter	pittii	3.89E-07 [0.00E+00-6.31E-06]	0.00E+00 [0.00E+00-0.00E+00]	-7.97±2.12	3.37E-03
Proteobacteria	Gammaproteobacteria	Enterobacterales	Enterobacteriaceae	Yersinia	massiliensis	0.00E+00 [0.00E+00-8.97E-07]	0.00E+00 [0.00E+00-0.00E+00]	-7.97±2.24	5.56E-03
Firmicutes	Bacilli	Lactobacillales	Streptococcaceae	Streptococcus	anginosus	3.89E-07 [0.00E+00-1.11E-06]	2.51E-07 [0.00E+00-9.65E-07]	-7.41±1.56	8.83E-05
Proteobacteria	Gammaproteobacteria	Enterobacterales	Enterobacteriaceae	Mangrovibacter	phragmitis	0.00E+00 [0.00E+00-1.21E-06]	0.00E+00 [0.00E+00-0.00E+00]	-7.24±2.20	1.21E-02
Proteobacteria	Gammaproteobacteria	Enterobacterales	Enterobacteriaceae	Edwardsiella	piscicida	0.00E+00 [0.00E+00-2.38E-07]	0.00E+00 [0.00E+00-1.89E-07]	-6.95±1.95	5.56E-03
Proteobacteria	Gammaproteobacteria	Enterobacterales	Enterobacteriaceae	Klebsiella_A	unknown	2.14E-06 [4.05E-07-5.84E-06]	1.61E-07 [0.00E+00-3.19E-06]	-6.45±1.56	9.05E-04
Actinobacteriota	Actinomycetia	Actinomycetales	Micrococcaceae	unknown	unknown	3.70E-07 [0.00E+00-1.75E-06]	0.00E+00 [0.00E+00-2.76E-07]	-5.80±1.94	2.70E-02
Proteobacteria	Gammaproteobacteria	Enterobacterales	Enterobacteriaceae	Kosakonia	radicincitans	1.95E-07 [0.00E+00-5.58E-06]	0.00E+00 [0.00E+00-5.52E-07]	-5.79±1.77	1.25E-02
Proteobacteria	Gammaproteobacteria	Enterobacterales	Enterobacteriaceae	Citrobacter	braakii	3.67E-04 [1.06E-04-1.42E-03]	6.55E-05 [1.55E-05-1.15E-04]	-5.70±1.31	4.58E-04
Proteobacteria	Gammaproteobacteria	Enterobacterales	Enterobacteriaceae	Raoultella	ornithinolytica	1.94E-06 [5.84E-07-7.00E-06]	1.89E-07 [0.00E+00-6.37E-06]	-5.69±1.60	5.56E-03
Proteobacteria	Gammaproteobacteria	Enterobacterales	Enterobacteriaceae	Cronobacter	dublinensis	8.97E-07 [4.75E-07-3.89E-06]	0.00E+00 [0.00E+00-3.31E-06]	-5.67±1.56	4.73E-03
Proteobacteria	Gammaproteobacteria	Enterobacterales	Enterobacteriaceae	Salmonella	bongori	7.13E-07 [0.00E+00-2.49E-06]	0.00E+00 [0.00E+00-1.61E-07]	-5.31±1.90	4.09E-02
Firmicutes	Bacilli	Mycoplasmatales	Metamycoplasmataceae	Metamycoplasma	hominis	2.43E-07 [0.00E+00-2.34E-06]	0.00E+00 [0.00E+00-0.00E+00]	-5.09±1.79	3.67E-02
Proteobacteria	Gammaproteobacteria	Enterobacterales	Enterobacteriaceae	Cronobacter	malonaticus	4.07E-06 [7.79E-07-1.65E-05]	1.06E-06 [0.00E+00-5.48E-06]	-4.99±1.60	1.81E-02
Firmicutes_A	Clostridia	Peptostreptococcales	Anaerovoracaceae	Emergencia	timonensis	3.59E-06 [1.85E-06-2.70E-05]	3.22E-07 [2.85E-07-1.74E-06]	-4.97±0.96	1.06E-05
Proteobacteria	Gammaproteobacteria	Enterobacterales	Enterobacteriaceae	Citrobacter	europaeus	6.23E-06 [1.74E-07-6.34E-05]	5.70E-07 [0.00E+00-1.30E-05]	-4.77±1.70	4.09E-02
Firmicutes_A	Clostridia	Oscillospirales	Oscillospiraceae	Flavonifractor	sp000508885	8.99E-07 [0.00E+00-3.24E-06]	2.66E-07 [0.00E+00-6.20E-07]	-4.75±1.66	3.67E-02
Actinobacteriota	Actinomycetia	Propionibacteriales	Propionibacteriaceae	Propionibacterium	freudenreichii	7.28E-07 [0.00E+00-2.02E-06]	1.61E-07 [0.00E+00-2.66E-07]	-4.46±1.45	2.16E-02
Firmicutes	Bacilli	Lactobacillales	Enterococcaceae	Enterococcus_B	faecium_B	5.70E-04 [5.26E-05-2.53E-03]	4.76E-04 [1.72E-04-3.09E-03]	-4.35±1.37	1.61E-02
Proteobacteria	Gammaproteobacteria	Enterobacterales	Enterobacteriaceae	Enterobacter	roggenkampii	8.73E-06 [1.43E-06-9.89E-06]	5.01E-07 [1.61E-07-1.38E-05]	-4.26±1.46	3.18E-02
Firmicutes	Bacilli	Lactobacillales	Enterococcaceae	Enterococcus_B	unknown	1.24E-04 [4.45E-06-2.82E-03]	3.00E-04 [4.43E-05-7.92E-04]	-3.88±1.33	3.18E-02
Firmicutes	Bacilli	Lactobacillales	Streptococcaceae	Streptococcus	pasteurianus	1.10E-04 [3.24E-06-6.52E-04]	1.05E-05 [6.20E-06-3.80E-05]	-3.61±1.01	5.36E-03
Actinobacteriota	Actinomycetia	Actinomycetales	Actinomycetaceae	Buchananella	hordeovulneris	1.78E-05 [5.23E-06-2.83E-05]	4.56E-06 [1.45E-06-7.52E-06]	-3.24±0.96	9.20E-03
Actinobacteriota	Actinomycetia	Mycobacteriales	Mycobacteriaceae	Corynebacterium	mustelae	2.44E-05 [9.34E-06-1.13E-04]	5.01E-06 [1.17E-06-1.04E-05]	-3.17±1.14	4.25E-02

Firmicutes	Bacilli	Lactobacillales	Streptococcaceae	Streptococcus	thermophilus	1.63E-05 [5.58E-06-4.63E-05]	7.01E-06 [5.22E-06-1.37E-05]	-2.91±1.01	3.52E-02
**Higher in HR at baseline (59 species)**									
Bacteroidota	Bacteroidia	Bacteroidales	Marinifilaceae	Butyricimonas	virosa	0.00E+00 [0.00E+00-0.00E+00]	0.00E+00 [0.00E+00-5.22E-06]	21.06±2.73	3.96E-12
Firmicutes_A	Clostridia_A	Christensenellales	Christensenellaceae	Christensenella	minuta	0.00E+00 [0.00E+00-0.00E+00]	0.00E+00 [0.00E+00-0.00E+00]	20.91±3.11	2.83E-09
Bacteroidota	Bacteroidia	Bacteroidales	Bacteroidaceae	Prevotellamassilia	timonensis	0.00E+00 [0.00E+00-0.00E+00]	0.00E+00 [0.00E+00-1.00E-06]	17.99±2.86	3.99E-08
Bacteroidota	Bacteroidia	Bacteroidales	Bacteroidaceae	Prevotella	unknown	0.00E+00 [0.00E+00-0.00E+00]	0.00E+00 [0.00E+00-3.78E-07]	17.17±2.79	7.00E-08
Actinobacteriota	Actinomycetia	Mycobacteriales	Mycobacteriaceae	Mycobacterium	unknown	0.00E+00 [0.00E+00-0.00E+00]	2.76E-07 [0.00E+00-1.00E-06]	17.05±1.95	1.55E-15
Bacteroidota	Bacteroidia	unknown	unknown	unknown	unknown	0.00E+00 [0.00E+00-0.00E+00]	0.00E+00 [0.00E+00-1.17E-06]	16.84±3.00	1.28E-06
Actinobacteriota	Actinomycetia	Actinomycetales	Bifidobacteriaceae	Bifidobacterium	longum	0.00E+00 [0.00E+00-0.00E+00]	0.00E+00 [0.00E+00-0.00E+00]	15.70±2.29	1.67E-09
Actinobacteriota	Actinomycetia	Actinomycetales	Bifidobacteriaceae	Bifidobacterium	animalis	0.00E+00 [0.00E+00-0.00E+00]	0.00E+00 [0.00E+00-0.00E+00]	14.92±3.38	3.83E-04
Desulfobacterota	Desulfovibrionia	Desulfovibrionales	Desulfovibrionaceae	Bilophila	wadsworthia	0.00E+00 [0.00E+00-0.00E+00]	0.00E+00 [0.00E+00-5.58E-06]	12.93±3.69	6.10E-03
Actinobacteriota	Coriobacteriia	Coriobacteriales	Eggerthellaceae	Slackia_A	piriformis	0.00E+00 [0.00E+00-0.00E+00]	0.00E+00 [0.00E+00-0.00E+00]	11.54±3.64	1.68E-02
Firmicutes	Erysipelotrichia	Erysipelotrichales	Erysipelotrichaceae	Catenibacterium	mitsuokai	0.00E+00 [0.00E+00-0.00E+00]	0.00E+00 [0.00E+00-4.82E-07]	11.10±2.72	1.08E-03
Bacteroidota	Bacteroidia	Bacteroidales	Bacteroidaceae	Bacteroides	stercoris	8.37E-06 [7.64E-06-1.78E-04]	8.09E-04 [4.50E-06-1.62E-02]	8.72±1.60	2.92E-06
Bacteroidota	Bacteroidia	Bacteroidales	Bacteroidaceae	Phocaeicola	mediterraneensis	0.00E+00 [0.00E+00-1.95E-07]	1.66E-06 [0.00E+00-8.11E-06]	8.57±2.10	1.11E-03
Bacteroidota	Bacteroidia	Bacteroidales	Bacteroidaceae	Phocaeicola	salanitronis	0.00E+00 [0.00E+00-0.00E+00]	2.21E-06 [0.00E+00-1.14E-05]	7.91±1.95	1.12E-03
Actinobacteriota	Actinomycetia	Actinomycetales	Bifidobacteriaceae	Bifidobacterium	infantis	0.00E+00 [0.00E+00-4.05E-07]	0.00E+00 [0.00E+00-1.00E-06]	7.71±2.28	9.20E-03
Bacteroidota	Bacteroidia	Bacteroidales	Bacteroidaceae	Bacteroides	xylanisolvens	2.38E-07 [0.00E+00-1.60E-05]	1.87E-05 [4.25E-06-5.47E-04]	7.70±1.58	4.77E-05
Bacteroidota	Bacteroidia	Bacteroidales	Tannerellaceae	Parabacteroides	unknown	0.00E+00 [0.00E+00-3.59E-07]	0.00E+00 [0.00E+00-2.29E-05]	7.61±1.86	1.08E-03
Firmicutes	Bacilli	Haloplasmatales	Turicibacteraceae	Turicibacter	sanguinis	0.00E+00 [0.00E+00-5.22E-07]	1.16E-05 [0.00E+00-3.81E-04]	7.40±1.73	5.74E-04
Bacteroidota	Bacteroidia	Bacteroidales	Bacteroidaceae	Bacteroides	finegoldii	0.00E+00 [0.00E+00-2.34E-06]	5.52E-07 [0.00E+00-3.45E-04]	6.82±1.74	2.06E-03
Firmicutes_A	Clostridia	Lachnospirales	Lachnospiraceae	Enterocloster	unknown	1.95E-07 [0.00E+00-8.09E-07]	4.96E-06 [8.55E-07-9.52E-06]	6.60±1.31	2.18E-05
Bacteroidota	Bacteroidia	Bacteroidales	Bacteroidaceae	Bacteroides	bouchesdurhonensis	0.00E+00 [0.00E+00-1.95E-07]	0.00E+00 [0.00E+00-4.67E-06]	6.58±1.86	5.65E-03
Bacteroidota	Bacteroidia	Bacteroidales	Tannerellaceae	Parabacteroides	johnsonii	0.00E+00 [0.00E+00-7.79E-07]	8.69E-06 [0.00E+00-4.16E-05]	6.50±1.77	4.60E-03
Firmicutes	Bacilli	Lactobacillales	Enterococcaceae	Enterococcus_C	dispar	0.00E+00 [0.00E+00-9.51E-07]	1.06E-06 [0.00E+00-1.87E-05]	6.40±1.67	2.69E-03
Firmicutes_A	Clostridia	Lachnospirales	Lachnospiraceae	Blautia_A	sp000433815	1.46E-06 [2.38E-07-1.65E-05]	6.70E-05 [2.65E-06-2.09E-03]	6.37±1.46	4.26E-04
Firmicutes_A	Clostridia	Lachnospirales	Lachnospiraceae	Clostridium_Q	symbiosum	0.00E+00 [0.00E+00-3.70E-07]	3.04E-06 [1.55E-06-1.04E-05]	6.34±1.52	8.44E-04
Bacteroidota	Bacteroidia	Bacteroidales	Bacteroidaceae	Bacteroides	togonis	0.00E+00 [0.00E+00-0.00E+00]	2.51E-07 [0.00E+00-8.26E-06]	6.26±2.15	3.18E-02
Bacteroidota	Bacteroidia	Bacteroidales	Bacteroidaceae	Paraprevotella	xylaniphila	0.00E+00 [0.00E+00-4.85E-07]	2.90E-07 [0.00E+00-7.54E-05]	6.18±2.07	2.70E-02
Bacteroidota	Bacteroidia	Bacteroidales	Tannerellaceae	Parabacteroides	merdae	3.89E-07 [0.00E+00-7.28E-07]	1.29E-05 [3.22E-07-2.31E-05]	6.00±1.69	5.56E-03
Bacteroidota	Bacteroidia	Bacteroidales	Bacteroidaceae	Phocaeicola	sartorii	0.00E+00 [0.00E+00-0.00E+00]	1.42E-05 [0.00E+00-7.98E-05]	5.80±1.85	1.81E-02
Firmicutes_A	Clostridia	Oscillospirales	Ruminococcaceae	Angelakisella	massiliensis	0.00E+00 [0.00E+00-1.79E-07]	2.66E-07 [0.00E+00-1.38E-06]	5.77±1.51	2.69E-03
Bacteroidota	Bacteroidia	Bacteroidales	Bacteroidaceae	Phocaeicola	plebeius	2.92E-06 [0.00E+00-1.25E-05]	1.54E-04 [9.65E-07-6.80E-04]	5.67±1.60	5.56E-03
Firmicutes_A	Clostridia	Lachnospirales	Lachnospiraceae	Enterocloster	aldenensis	0.00E+00 [0.00E+00-0.00E+00]	1.61E-07 [0.00E+00-1.71E-06]	5.54±1.93	3.52E-02
Bacteroidota	Bacteroidia	Bacteroidales	Coprobacteraceae	Coprobacter	fastidiosus	0.00E+00 [0.00E+00-0.00E+00]	3.50E-06 [0.00E+00-1.10E-05]	5.46±1.87	3.18E-02
Firmicutes	Bacilli	Erysipelotrichales	Erysipelotrichaceae	Longicatena	sp900411325	3.59E-07 [0.00E+00-3.64E-06]	1.88E-04 [3.22E-07-1.60E-03]	5.38±1.62	1.11E-02

Actinobacteriota	Actinomycetia	Actinomycetales	Bifidobacteriaceae	Bifidobacterium	globosum	1.74E-07 [0.00E+00-2.43E-07]	8.55E-07 [0.00E+00-1.45E-06]	5.37±1.71	1.75E-02
Bacteroidota	Bacteroidia	Bacteroidales	Bacteroidaceae	Bacteroides	sp900066265	0.00E+00 [0.00E+00-0.00E+00]	9.35E-06 [0.00E+00-3.79E-05]	5.35±1.75	2.17E-02
Bacteroidota	Bacteroidia	Bacteroidales	Bacteroidaceae	Bacteroides	nordii	0.00E+00 [0.00E+00-3.64E-06]	0.00E+00 [0.00E+00-2.65E-05]	5.28±1.89	4.13E-02
Firmicutes_A	Clostridia	Oscillospirales	Oscillospiraceae	Flavonifractor	plautii	4.05E-07 [0.00E+00-3.11E-06]	3.48E-06 [1.99E-06-7.01E-06]	5.28±1.49	5.56E-03
Bacteroidota	Bacteroidia	Bacteroidales	Bacteroidaceae	Phocaeicola	massiliensis	0.00E+00 [0.00E+00-1.95E-07]	5.48E-06 [0.00E+00-1.40E-05]	5.22±1.78	3.15E-02
Firmicutes_A	Clostridia	Lachnospirales	Lachnospiraceae	Blautia_A	sp900541345	8.79E-06 [9.70E-07-1.27E-05]	1.23E-04 [1.65E-05-6.28E-04]	5.13±1.41	4.73E-03
Bacteroidota	Bacteroidia	Bacteroidales	Bacteroidaceae	unknown	unknown	5.51E-05 [0.00E+00-1.69E-04]	1.07E-03 [2.41E-06-3.87E-03]	4.90±1.50	1.26E-02
Firmicutes_A	Clostridia	Lachnospirales	Lachnospiraceae	Blautia_A	sp900540785	1.20E-05 [1.70E-06-3.37E-05]	2.22E-04 [3.54E-05-1.19E-03]	4.89±1.15	5.74E-04
Firmicutes_A	Clostridia	Lachnospirales	Lachnospiraceae	Schaedlerella	glycyrrhizinilyticum	4.49E-06 [1.94E-06-6.62E-06]	7.91E-05 [4.03E-05-1.39E-04]	4.85±1.07	2.07E-04
Firmicutes_A	Clostridia	Oscillospirales	Oscillospiraceae	unknown	unknown	0.00E+00 [0.00E+00-0.00E+00]	5.70E-07 [4.82E-07-2.13E-06]	4.79±1.72	4.23E-02
Actinobacteriota	Actinomycetia	Mycobacteriales	Mycobacteriaceae	Corynebacterium	unknown	2.49E-07 [0.00E+00-2.85E-06]	2.66E-07 [1.89E-07-6.20E-07]	4.73±1.58	2.69E-02
Firmicutes	Bacilli	Lactobacillales	Enterococcaceae	Enterococcus_D	casseliflavus	1.79E-06 [1.11E-06-7.76E-06]	1.59E-04 [5.14E-06-9.96E-04]	4.63±1.66	4.09E-02
Firmicutes	Bacilli	Lactobacillales	Enterococcaceae	Enterococcus_D	gallinarum	1.35E-06 [0.00E+00-4.03E-05]	2.48E-05 [6.27E-06-2.32E-03]	4.38±1.31	1.06E-02
Firmicutes_A	Clostridia	Lachnospirales	Lachnospiraceae	Agathobacter	faecis	0.00E+00 [0.00E+00-0.00E+00]	1.55E-06 [8.04E-07-9.83E-06]	4.33±1.27	8.45E-03
Firmicutes_A	Clostridia	Lachnospirales	Lachnospiraceae	Blautia_A	unknown	1.73E-05 [1.60E-05-8.00E-05]	2.57E-04 [3.81E-05-6.42E-04]	4.15±1.13	4.47E-03
Firmicutes	Bacilli	Lactobacillales	Streptococcaceae	Streptococcus	parauberis	7.18E-07 [0.00E+00-2.99E-06]	2.85E-06 [2.27E-06-7.23E-06]	4.15±1.31	1.74E-02
Firmicutes_A	Clostridia	Lachnospirales	Lachnospiraceae	Blautia	sp003287895	7.54E-06 [1.21E-06-1.62E-05]	8.64E-05 [4.27E-06-2.84E-04]	4.11±1.13	4.73E-03
Firmicutes_A	Clostridia	Oscillospirales	Ruminococcaceae	unknown	unknown	0.00E+00 [0.00E+00-3.70E-07]	8.28E-07 [2.51E-07-9.65E-07]	4.04±1.10	4.60E-03
Firmicutes_A	Clostridia	Lachnospirales	Lachnospiraceae	Dorea	sp900240315	2.49E-06 [4.05E-07-4.85E-06]	2.84E-05 [1.08E-05-5.86E-05]	3.68±1.02	4.87E-03
Firmicutes_A	Clostridia	Lachnospirales	Lachnospiraceae	Enterocloster	bolteae	2.83E-06 [2.43E-07-8.98E-06]	2.09E-05 [1.68E-05-4.81E-05]	3.52±1.09	1.44E-02
Firmicutes_A	Clostridia	Lachnospirales	Lachnospiraceae	Faecalimonas	nexilis	8.23E-05 [1.74E-05-1.04E-04]	2.76E-04 [1.45E-04-6.54E-04]	3.46±0.98	5.65E-03
Firmicutes_A	Clostridia	Oscillospirales	unknown	unknown	unknown	3.31E-06 [1.08E-06-6.42E-06]	1.35E-05 [6.43E-06-1.52E-05]	3.10±0.71	3.86E-04
Firmicutes	Bacilli	Lactobacillales	Streptococcaceae	Streptococcus	suis	1.22E-06 [0.00E+00-2.25E-06]	3.46E-06 [1.16E-06-1.16E-05]	2.77±0.98	3.79E-02
Firmicutes_A	Clostridia	Lachnospirales	Lachnospiraceae	Faecalimonas	sp900550235	1.32E-05 [4.18E-06-1.94E-05]	2.47E-04 [8.76E-05-2.98E-04]	2.71±0.99	4.73E-02
Firmicutes_A	Clostridia	Lachnospirales	Lachnospiraceae	Roseburia	inulinivorans	2.38E-07 [0.00E+00-2.34E-06]	6.52E-06 [3.54E-06-9.27E-06]	2.50±0.89	4.01E-02

FC: fold change, SE: standard error

[1] p values were adjusted with false discovery rate for multiple comparisons

Supplemental Table S15. Species with differential abundances between low responders (LR, n=8) and high responders (HR, n=8) in PBO at baseline (log_2|fold change| ≥ 2 and FDR-adjusted p < 0.05)

Phylum	Class	Order	Family	Genus	Species	Relative abundance (%) Median (IQR)		HR vs LR	
						LR	HR	Log 2 FC mean ± SE	Adjusted *P*-value[1]
**Higher in LR at baseline (55 species)**									
Firmicutes_A	Clostridia_A	Christensenellales	Christensenellaceae	unknown	unknown	0.00E+00 [0.00E+00-8.60E-06]	0.00E+00 [0.00E+00-0.00E+00]	-24.35±5.51	2.98E-04
Firmicutes_A	Clostridia	Eubacteriales	Eubacteriaceae	Eubacterium	limosum	4.77E-07 [0.00E+00-7.59E-06]	0.00E+00 [0.00E+00-0.00E+00]	-20.37±3.36	4.63E-07
Firmicutes_A	Clostridia	Oscillospirales	Oscillospiraceae	Flavonifractor	unknown	0.00E+00 [0.00E+00-0.00E+00]	0.00E+00 [0.00E+00-0.00E+00]	-15.54±3.24	6.08E-05
Firmicutes_A	Clostridia_A	Christensenellales	Christensenellaceae	Christensenella	minuta	0.00E+00 [0.00E+00-2.82E-04]	0.00E+00 [0.00E+00-1.78E-07]	-12.49±3.27	2.50E-03
Firmicutes	Bacilli	Lactobacillales	Lactobacillaceae	Pediococcus	acidilactici	1.85E-07 [0.00E+00-6.70E-03]	0.00E+00 [0.00E+00-4.04E-07]	-12.22±2.12	1.90E-06
Proteobacteria	Gammaproteobacteria	Enterobacterales	Enterobacteriaceae	Phytobacter	massiliensis	1.23E-07 [0.00E+00-4.27E-06]	0.00E+00 [0.00E+00-0.00E+00]	-12.15±2.62	1.21E-04
Actinobacteriota	Coriobacteriia	Coriobacteriales	Eggerthellaceae	Eggerthella	lenta	2.07E-07 [0.00E+00-4.65E-04]	0.00E+00 [0.00E+00-1.70E-06]	-12.11±2.45	4.77E-05
Firmicutes	Bacilli	Lactobacillales	Lactobacillaceae	Limosilactobacillus	unknown	2.32E-07 [0.00E+00-3.87E-06]	0.00E+00 [0.00E+00-0.00E+00]	-9.71±2.35	8.76E-04
Bacteroidota	Bacteroidia	Bacteroidales	Bacteroidaceae	Paraprevotella	xylaniphila	2.35E-06 [0.00E+00-2.19E-05]	5.44E-07 [0.00E+00-1.03E-06]	-9.64±2.21	3.33E-04
Proteobacteria	Gammaproteobacteria	Enterobacterales	Enterobacteriaceae	Enterobacter	ludwigii	1.80E-06 [4.50E-07-3.58E-06]	0.00E+00 [0.00E+00-3.81E-07]	-9.39±1.75	6.60E-06
Proteobacteria	Gammaproteobacteria	Enterobacterales	Enterobacteriaceae	Atlantibacter	hermannii	1.50E-07 [0.00E+00-1.40E-06]	0.00E+00 [0.00E+00-0.00E+00]	-9.24±2.47	3.03E-03
Firmicutes	Bacilli	Lactobacillales	Lactobacillaceae	Limosilactobacillus	reuteri	1.96E-07 [0.00E+00-1.93E-04]	0.00E+00 [0.00E+00-2.02E-07]	-8.63±2.25	2.38E-03
Firmicutes_A	Clostridia	Oscillospirales	Oscillospiraceae	Flavonifractor	plautii	1.79E-06 [0.00E+00-6.56E-06]	1.11E-06 [0.00E+00-1.97E-06]	-7.79±1.60	5.61E-05
Bacteroidota	Bacteroidia	Bacteroidales	Bacteroidaceae	Bacteroides	xylanisolvens	3.52E-04 [1.54E-06-4.19E-03]	6.38E-06 [2.59E-07-2.98E-05]	-7.59±1.68	1.86E-04
Proteobacteria	Gammaproteobacteria	Enterobacterales	Enterobacteriaceae	Leclercia	adecarboxylata	1.57E-07 [0.00E+00-3.88E-07]	0.00E+00 [0.00E+00-0.00E+00]	-7.28±2.17	1.11E-02
Bacteroidota	Bacteroidia	Bacteroidales	Bacteroidaceae	Bacteroides	helcogenes	0.00E+00 [0.00E+00-5.29E-05]	0.00E+00 [0.00E+00-8.02E-07]	-7.23±2.05	6.55E-03
Proteobacteria	Gammaproteobacteria	Enterobacterales	Enterobacteriaceae	Franconibacter	helveticus	0.00E+00 [0.00E+00-0.00E+00]	0.00E+00 [0.00E+00-0.00E+00]	-7.23±2.53	3.55E-02
Firmicutes	Bacilli	Lactobacillales	Enterococcaceae	Enterococcus_D	casseliflavus	3.78E-05 [1.40E-05-4.01E-04]	1.24E-05 [5.03E-06-4.41E-05]	-7.14±1.76	1.15E-03
Proteobacteria	Gammaproteobacteria	Enterobacterales	Enterobacteriaceae	Escherichia	coli_D	8.58E-03 [5.15E-03-5.70E-02]	1.76E-03 [8.71E-04-3.37E-03]	-6.54±1.28	2.27E-05
Firmicutes	Bacilli	Paenibacillales	Paenibacillaceae	Paenibacillus	sp000499205	2.61E-06 [1.75E-06-8.77E-06]	0.00E+00 [0.00E+00-4.71E-07]	-6.47±1.84	6.55E-03
Firmicutes	Bacilli	Lactobacillales	Enterococcaceae	Enterococcus_D	unknown	3.87E-05 [1.48E-05-2.38E-04]	4.02E-06 [6.70E-07-4.46E-05]	-6.44±1.78	4.89E-03
Bacteroidota	Bacteroidia	Bacteroidales	Bacteroidaceae	Bacteroides	ovatus	1.09E-03 [0.00E+00-9.26E-03]	4.35E-05 [7.00E-06-1.50E-04]	-6.43±1.63	1.63E-03
Firmicutes_A	Clostridia	Clostridiales	Clostridiaceae	Clostridium	thermobutyricum	1.91E-07 [0.00E+00-6.59E-06]	1.08E-07 [0.00E+00-1.33E-06]	-6.35±2.18	3.08E-02
Proteobacteria	Gammaproteobacteria	Enterobacterales	Enterobacteriaceae	Citrobacter_A	amalonaticus_C	2.42E-07 [0.00E+00-6.24E-07]	0.00E+00 [0.00E+00-2.88E-08]	-6.32±1.91	1.19E-02
Proteobacteria	Gammaproteobacteria	Enterobacterales	Enterobacteriaceae	Raoultella	unknown	7.48E-07 [9.23E-08-2.05E-06]	0.00E+00 [0.00E+00-2.88E-08]	-6.05±1.98	2.27E-02
Bacteroidota	Bacteroidia	Bacteroidales	Bacteroidaceae	Phocaeicola	sartorii	4.32E-05 [0.00E+00-1.53E-04]	1.78E-07 [0.00E+00-1.15E-05]	-6.01±1.95	2.22E-02
Proteobacteria	Gammaproteobacteria	Enterobacterales	Enterobacteriaceae	Raoultella	ornithinolytica	1.26E-06 [9.88E-07-3.46E-06]	0.00E+00 [0.00E+00-1.93E-06]	-5.92±1.73	8.76E-03
Bacteroidota	Bacteroidia	Bacteroidales	Coprobacteraceae	Coprobacter	fastidiosus	0.00E+00 [0.00E+00-2.16E-06]	2.37E-07 [0.00E+00-6.81E-07]	-5.92±1.94	2.30E-02

Firmicutes	Bacilli	Lactobacillales	Enterococcaceae	Enterococcus_A	gilvus	1.84E-05 [1.68E-06-6.44E-04]	5.69E-06 [1.50E-06-1.26E-05]	-5.88±1.34	3.06E-04
Firmicutes	Bacilli	Bacillales	Planococcaceae	Rummeliibacillus	unknown	1.29E-05 [2.63E-06-2.10E-03]	6.10E-06 [3.43E-06-2.97E-05]	-5.80±1.02	2.11E-06
Firmicutes_A	Clostridia	Lachnospirales	Lachnospiraceae	Eisenbergiella	unknown	0.00E+00 [0.00E+00-2.39E-07]	0.00E+00 [0.00E+00-1.08E-07]	-5.73±1.91	2.63E-02
Proteobacteria	Gammaproteobacteria	Enterobacterales	Enterobacteriaceae	Enterobacter	sesami	2.18E-06 [1.54E-06-4.92E-06]	5.75E-08 [0.00E+00-4.19E-06]	-5.66±1.47	2.33E-03
Firmicutes	Bacilli	Lactobacillales	Lactobacillaceae	Ligilactobacillus	salivarius	6.52E-06 [9.23E-08-3.51E-05]	0.00E+00 [0.00E+00-2.69E-07]	-5.63±1.89	2.77E-02
Bacteroidota	Bacteroidia	Bacteroidales	Rikenellaceae	Alistipes_A	sp900240235	3.31E-06 [0.00E+00-3.63E-05]	0.00E+00 [0.00E+00-8.63E-08]	-5.63±2.04	4.59E-02
Firmicutes	Bacilli	Lactobacillales	Enterococcaceae	Enterococcus_A	hermanniensis	2.08E-06 [9.26E-07-3.95E-06]	0.00E+00 [0.00E+00-4.15E-06]	-5.37±1.75	2.24E-02
Proteobacteria	Gammaproteobacteria	Enterobacterales	Enterobacteriaceae	Pantoea	ananatis	6.15E-08 [0.00E+00-4.50E-07]	0.00E+00 [0.00E+00-1.46E-06]	-5.36±1.88	3.55E-02
Proteobacteria	Gammaproteobacteria	Enterobacterales	Enterobacteriaceae	Citrobacter_B	koseri	7.50E-06 [3.46E-06-2.60E-05]	5.43E-06 [0.00E+00-1.72E-05]	-4.96±1.34	3.48E-03
Proteobacteria	Gammaproteobacteria	Enterobacterales	Enterobacteriaceae	Enterobacter	kobei	8.94E-06 [1.46E-06-1.30E-05]	1.73E-07 [0.00E+00-3.48E-06]	-4.94±1.46	9.80E-03
Firmicutes	Bacilli	Lactobacillales	Enterococcaceae	Enterococcus_D	sp002850555	7.15E-06 [1.47E-06-7.10E-05]	4.14E-07 [0.00E+00-3.99E-06]	-4.89±1.81	4.94E-02
Bacteroidota	Bacteroidia	Bacteroidales	Bacteroidaceae	unknown	unknown	1.10E-03 [2.10E-06-2.13E-03]	5.20E-04 [7.89E-05-1.68E-03]	-4.87±1.59	2.27E-02
Proteobacteria	Gammaproteobacteria	Enterobacterales	Enterobacteriaceae	Enterobacter	unknown	6.74E-06 [3.29E-06-1.11E-05]	1.21E-06 [0.00E+00-2.50E-06]	-4.81±1.49	1.53E-02
Firmicutes_A	Clostridia	Lachnospirales	Lachnospiraceae	Enterocloster	sp000155435	0.00E+00 [0.00E+00-1.29E-06]	0.00E+00 [0.00E+00-8.96E-07]	-4.52±1.67	4.94E-02
Actinobacteriota	Actinomycetia	Propionibacteriales	Propionibacteriaceae	Propionibacterium	freudenreichii	1.72E-06 [0.00E+00-5.24E-06]	1.08E-07 [0.00E+00-3.68E-07]	-4.49±1.55	3.27E-02
Proteobacteria	Gammaproteobacteria	Enterobacterales	Enterobacteriaceae	Escherichia	sp000208585	2.76E-04 [1.46E-04-4.55E-03]	4.53E-05 [2.17E-05-4.55E-04]	-4.42±1.27	7.41E-03
Firmicutes_A	Clostridia	Lachnospirales	Cellulosilyticaceae	Niameybacter	sp900549765	2.97E-05 [6.00E-07-3.00E-04]	3.95E-06 [1.48E-06-6.72E-06]	-4.33±1.20	5.03E-03
Proteobacteria	Gammaproteobacteria	Enterobacterales	Enterobacteriaceae	Citrobacter	murliniae	6.02E-06 [2.70E-06-1.41E-05]	1.09E-06 [0.00E+00-4.74E-06]	-4.19±1.48	3.64E-02
Proteobacteria	Gammaproteobacteria	Enterobacterales	Enterobacteriaceae	Escherichia	fergusonii	7.94E-04 [4.13E-04-2.50E-03]	1.42E-04 [5.12E-05-5.27E-04]	-4.18±1.25	1.14E-02
Proteobacteria	unknown	unknown	unknown	unknown	unknown	4.64E-06 [7.98E-07-8.30E-05]	3.43E-06 [1.26E-06-1.70E-05]	-3.90±1.30	2.63E-02
Proteobacteria	Gammaproteobacteria	Enterobacterales	Enterobacteriaceae	Klebsiella_A	michiganensis	4.44E-06 [9.95E-07-2.49E-05]	1.49E-06 [0.00E+00-1.03E-05]	-3.83±1.37	3.99E-02
Proteobacteria	Gammaproteobacteria	Enterobacterales	Enterobacteriaceae	Escherichia	dysenteriae	4.25E-04 [2.80E-04-1.56E-03]	1.38E-04 [3.49E-05-6.26E-04]	-3.77±1.29	3.08E-02
Firmicutes	Bacilli	Erysipelotrichales	Erysipelotrichaceae	Longicatena	caecimuris	1.79E-06 [0.00E+00-2.64E-06]	1.31E-06 [5.27E-07-1.96E-06]	-3.50±1.10	1.71E-02
Proteobacteria	Gammaproteobacteria	Enterobacterales	Enterobacteriaceae	Escherichia	coli	1.07E-01 [3.56E-02-1.94E-01]	5.31E-03 [1.91E-03-7.65E-03]	-3.48±1.28	4.91E-02
Firmicutes_A	Clostridia	Peptostreptococcales	Peptostreptococcaceae	Clostridioides	difficile	2.29E-05 [6.95E-06-5.58E-05]	3.88E-05 [2.74E-05-6.74E-05]	-3.19±1.11	3.45E-02
Firmicutes_A	Clostridia	Lachnospirales	Lachnospiraceae	Hungatella	unknown	6.98E-06 [3.01E-06-1.15E-05]	5.29E-06 [1.93E-06-1.38E-05]	-3.15±1.01	2.07E-02
Firmicutes_A	Clostridia	Lachnospirales	Lachnospiraceae	Blautia_A	wexlerae	7.67E-05 [5.77E-05-1.54E-04]	1.71E-04 [3.98E-05-2.35E-04]	-2.47±0.71	7.13E-03
**Higher in HR at baseline (38 species)**									
Firmicutes_A	Clostridia	Oscillospirales	Ruminococcaceae	Ruminococcus_C	callidus	0.00E+00 [0.00E+00-0.00E+00]	4.19E-07 [0.00E+00-2.03E-06]	19.46±2.83	4.09E-09
Actinobacteriota	Coriobacteriia	Coriobacteriales	Eggerthellaceae	Slackia_A	unknown	0.00E+00 [0.00E+00-0.00E+00]	0.00E+00 [0.00E+00-1.02E-05]	19.30±7.09	4.91E-02
Actinobacteriota	Coriobacteriia	Coriobacteriales	Eggerthellaceae	Slackia_A	piriformis	0.00E+00 [0.00E+00-0.00E+00]	0.00E+00 [0.00E+00-7.73E-07]	18.30±3.81	6.08E-05
Firmicutes	Bacilli	Erysipelotrichales	Erysipelatoclostridiaceae	Catenibacterium	mitsuokai	0.00E+00 [0.00E+00-0.00E+00]	0.00E+00 [0.00E+00-1.02E-06]	16.58±2.95	2.29E-06
Actinobacteriota	Coriobacteriia	Coriobacteriales	Atopobiaceae	Olsenella_E	sp900119915	0.00E+00 [0.00E+00-0.00E+00]	0.00E+00 [0.00E+00-1.52E-06]	16.40±3.72	3.00E-04
Bacteroidota	Bacteroidia	Bacteroidales	Bacteroidaceae	Prevotellamassilia	timonensis	0.00E+00 [0.00E+00-0.00E+00]	0.00E+00 [0.00E+00-3.68E-07]	14.86±3.09	6.08E-05
Proteobacteria	Gammaproteobacteria	Enterobacterales	Enterobacteriaceae	Yersinia	massiliensis	0.00E+00 [0.00E+00-0.00E+00]	0.00E+00 [0.00E+00-0.00E+00]	13.93±2.48	2.29E-06
Firmicutes	Bacilli	Lactobacillales	Lactobacillaceae	Leuconostoc	carnosum	0.00E+00 [0.00E+00-1.47E-07]	6.04E-07 [0.00E+00-1.21E-04]	11.44±2.83	1.17E-03

Firmicutes	Bacilli	Lactobacillales	Lactobacillaceae	Leuconostoc	kimchii	0.00E+00 [0.00E+00-4.77E-08]	6.71E-07 [0.00E+00-4.74E-05]	11.08±3.63	2.27E-02
Firmicutes	Bacilli	Lactobacillales	Lactobacillaceae	Leuconostoc	mesenteroides	0.00E+00 [0.00E+00-0.00E+00]	8.87E-07 [8.63E-08-1.34E-04]	9.85±2.03	5.61E-05
Actinobacteriota	Coriobacteriia	Coriobacteriales	Coriobacteriaceae	unknown	unknown	0.00E+00 [0.00E+00-5.60E-08]	1.40E-06 [0.00E+00-9.33E-05]	9.61±2.89	1.14E-02
Fusobacteriota	Fusobacteriia	Fusobacteriales	Fusobacteriaceae	Fusobacterium_A	sp900543175	4.50E-07 [0.00E+00-2.74E-06]	6.24E-05 [5.03E-07-6.39E-04]	9.51±2.10	1.86E-04
Firmicutes	Bacilli	Lactobacillales	Lactobacillaceae	Latilactobacillus	unknown	0.00E+00 [0.00E+00-7.50E-08]	2.81E-06 [0.00E+00-1.74E-04]	9.35±2.36	1.59E-03
Proteobacteria	Gammaproteobacteria	Burkholderiales	Burkholderiaceae	Sutterella	wadsworthensis_A	0.00E+00 [0.00E+00-1.06E-06]	4.65E-06 [0.00E+00-2.92E-05]	9.19±2.42	2.62E-03
Firmicutes	Bacilli	Lactobacillales	Lactobacillaceae	Latilactobacillus	sakei	2.10E-07 [0.00E+00-1.00E-06]	1.85E-05 [1.05E-05-1.20E-03]	8.49±1.82	1.09E-04
Firmicutes	Bacilli	Lactobacillales	Lactobacillaceae	Leuconostoc	unknown	9.54E-08 [0.00E+00-2.31E-06]	3.87E-06 [6.86E-07-7.19E-04]	8.18±1.67	5.61E-05
Firmicutes	Bacilli	Lactobacillales	Lactobacillaceae	Leuconostoc	inhae	4.50E-07 [0.00E+00-1.11E-06]	2.23E-06 [4.31E-07-5.64E-04]	8.12±2.05	1.59E-03
Firmicutes	Bacilli	Lactobacillales	Lactobacillaceae	Latilactobacillus	sakei_A	2.86E-07 [0.00E+00-6.04E-07]	1.04E-05 [9.14E-07-4.02E-04]	7.83±1.80	3.33E-04
Firmicutes	Bacilli	Lactobacillales	Lactobacillaceae	Leuconostoc	gelidum	3.43E-06 [1.00E-06-1.24E-05]	1.36E-05 [6.39E-07-1.20E-03]	7.57±1.39	4.62E-06
Firmicutes	Bacilli	Lactobacillales	Streptococcaceae	Lactococcus	lactis	4.54E-06 [0.00E+00-6.74E-06]	3.77E-05 [2.18E-05-9.80E-04]	7.00±1.27	3.13E-06
Bacteroidota	Bacteroidia	Bacteroidales	Bacteroidaceae	Phocaeicola	coprocola	1.22E-06 [0.00E+00-7.95E-06]	4.70E-05 [1.73E-07-3.99E-04]	6.73±2.30	3.08E-02
Firmicutes	Bacilli	Lactobacillales	Brochotrichaceae	Brochothrix	thermosphacta	0.00E+00 [0.00E+00-1.11E-06]	1.01E-06 [0.00E+00-2.28E-06]	6.72±2.11	1.68E-02
Actinobacteriota	Coriobacteriia	Coriobacteriales	Coriobacteriaceae	Collinsella	intestinalis	2.60E-06 [9.52E-07-2.53E-05]	4.30E-02 [2.41E-06-1.05E-01]	6.51±2.17	2.63E-02
Actinobacteriota	Actinomycetia	Actinomycetales	Micrococcaceae	unknown	unknown	0.00E+00 [0.00E+00-1.09E-07]	0.00E+00 [0.00E+00-1.79E-06]	6.42±2.07	2.17E-02
Firmicutes_A	Clostridia	Oscillospirales	Ruminococcaceae	Faecalibacterium	sp900540455	1.91E-07 [0.00E+00-2.29E-06]	6.67E-06 [0.00E+00-3.17E-04]	5.97±1.58	2.71E-03
Firmicutes	Bacilli	Haloplasmatales	Turicibacteraceae	Turicibacter	sanguinis	9.92E-07 [1.43E-07-2.64E-06]	2.16E-07 [0.00E+00-7.61E-05]	5.79±1.85	1.98E-02
Firmicutes	Bacilli	Lactobacillales	Streptococcaceae	Lactococcus	piscium	0.00E+00 [0.00E+00-7.50E-08]	0.00E+00 [0.00E+00-7.73E-07]	5.74±2.04	3.96E-02
Firmicutes	Bacilli	Lactobacillales	Streptococcaceae	unknown	unknown	6.91E-06 [7.27E-07-5.79E-05]	5.40E-05 [2.07E-05-1.50E-04]	5.30±1.38	2.33E-03
Firmicutes	Bacilli	Lactobacillales	Lactobacillaceae	Weissella	cibaria	2.42E-07 [0.00E+00-1.02E-06]	3.38E-07 [8.63E-08-8.89E-05]	5.24±1.85	3.73E-02
Firmicutes	Bacilli	Lactobacillales	Streptococcaceae	Streptococcus	thermophilus	7.05E-06 [1.04E-06-1.88E-05]	7.99E-05 [5.06E-05-4.18E-04]	5.22±1.08	6.08E-05
Firmicutes	Bacilli	Lactobacillales	Lactobacillaceae	Lactiplantibacillus	plantarum	1.75E-06 [7.47E-07-4.03E-06]	1.29E-05 [2.63E-06-7.34E-05]	4.84±1.47	1.23E-02
Firmicutes	Bacilli	Haloplasmatales	Turicibacteraceae	Turicibacter	sp001543345	1.00E-05 [0.00E+00-4.22E-05]	4.09E-06 [3.24E-07-4.49E-05]	4.65±1.71	4.91E-02
Firmicutes	Bacilli	Lactobacillales	Lactobacillaceae	Leuconostoc	citreum	5.28E-07 [1.68E-07-1.07E-06]	3.70E-06 [2.20E-06-4.97E-05]	4.61±1.50	2.23E-02
Bacteroidota	Bacteroidia	Bacteroidales	Bacteroidaceae	Bacteroides	uniformis	9.69E-05 [1.16E-05-1.85E-04]	6.21E-05 [2.83E-05-2.47E-03]	4.13±1.40	2.98E-02
Firmicutes_A	Clostridia	Lachnospirales	CAG-274	UMGS1441	sp900543365	1.71E-06 [5.04E-07-2.52E-06]	8.63E-07 [0.00E+00-4.10E-06]	4.01±1.23	1.37E-02
Firmicutes_A	Clostridia	Lachnospirales	Lachnospiraceae	Blautia_A	sp900540785	8.33E-05 [3.27E-05-1.86E-04]	2.50E-04 [4.41E-06-1.41E-03]	3.95±1.21	1.37E-02
Firmicutes_A	Clostridia	Lachnospirales	Lachnospiraceae	Schaedlerella	glycyrrhizinilyticum	5.52E-05 [1.97E-05-1.23E-04]	2.52E-05 [1.21E-06-8.76E-04]	3.29±1.13	3.08E-02
Firmicutes_A	Clostridia	Lachnospirales	Lachnospiraceae	Robinsoniella	sp900555455	1.43E-05 [6.06E-06-1.77E-05]	4.10E-05 [1.73E-06-8.91E-05]	2.51±0.86	3.22E-02

FC: fold change, SE: standard error

[1] p values were adjusted with false discovery rate for multiple comparisons
